# A comparative genomic study across 396 liver biopsies provides deep insight into FGF21 mode of action as a therapeutic agent in metabolic dysfunction‐associated steatotic liver disease

**DOI:** 10.1002/ctm2.70218

**Published:** 2025-02-17

**Authors:** Shifang Tang, Jürgen Borlak

**Affiliations:** ^1^ Centre for Pharmacology and Toxicology Hannover Medical School Hannover Germany

**Keywords:** fibroblast growth factor‐21, fibrosis, inflammation, metabolic dysfunction‐associated steatotic liver disease, metabolism

## Abstract

**Background:**

Metabolic dysfunction‐associated steatotic liver disease (MASLD) is a systemic disease with insulin resistance at its core. It affects one‐third of the world population. Fibroblast growth factor (FGF21)‐based therapies are effective in lowering hepatic fat content and fibrosis resolution; yet, its molecular functions remain uncertain. To gain insight into FGF21 mode of action (MoA), we investigated the transcriptomes of MASLD liver biopsies in relation to FGF21 expression.

**Methods:**

We compared *N* = 66 healthy controls with 396 MASLD patients and considered clinical characteristics relative to NAS disease activity scores (steatosis, lobular inflammation and ballooning), fibrosis grades and sex. We performed comparative genomics to identify FGF21‐responsive DEGs, utilised information from FGF21‐transgenic and FGF21‐knockout mice and evaluated DEGs following FGF21 treatment of MASLD animal models. Eventually, we explored 188 validated FGF21 targets, and for ≥10 patients showing the same changes, we constructed MASLD‐associated networks to determine the effects of FGF21 in reverting metabolic dysfunction.

**Results:**

We identified patients with increased 30% (*N* = 117), decreased 40% (*N* = 159) or unchanged 30% (*N* = 120) FGF21 expression, and the differences are caused by changes in FGF21 transcriptional control with ATF4 functioning as a key regulator. Based on comparative genomics, we discovered molecular circuitries of FGF21 in MASLD, notably FGF21‐dependent induction of autophagy and oxidative phosphorylation/mitochondrial respiration. Conversely, FGF21 repressed hepatic glycogen‐storage, its glucose release and gluconeogenesis, and therefore reduced glucose flux in conditions of insulin resistance. Furthermore, FGF21 repressed lipid transporters, and acetyl‐CoA carboxylase‐β to attenuate hepatic lipid overload and lipogenesis. Strikingly, FGF21 dampened immune response by repressing complement factors, MARCO, CD163, MRC1/CD206, CD4, CD45 and pro‐inflammatory cytokine receptors. It also reverted procoagulant imbalance in MASLD, stimulated extracellular matrix degradation, repressed TGFβ‐ and integrin‐signalling and lessened liver sinusoidal endothelial cell defenestration in support of fibrosis resolution.

**Conclusions:**

We gained deep insight into FGF21‐MoA in MASLD. However, heterogeneity in FGF21 expression calls for molecular stratifications as to identify patients which likely benefit from FGF21‐based therapies.

**Key points:**

- Performed comprehensive genomics across liver biopsies of 396 MASLD patients and identified patients with increased, decreased and unchanged FGF21 expression.
- Used genomic data from FGF21 transgenic, knock‐out and animal MASLD models treated with synthetic FGF21 analogues to identify FGF21‐mode‐of‐action and metabolic networks in human MASLD.
- Given the significant heterogeneity in FGF21 expression, not all patients will benefit from FGF21‐based therapies.

## Graphical abstract

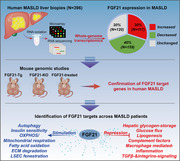

## 1 INTRODUCTION

Metabolic dysfunction‐associated steatotic liver disease (MASLD) is a major health burden with an estimated global prevalence of 30%.^1^ Recently, we reported the regulation of 18 drug targets across 418 MASLD liver biopsies of which 278 were metabolic dysfunction‐associated steatohepatitis (MASH) cases.^2^ Fibroblast growth factor 21 (FGF21) is a promising therapeutic agent for MASLD, and in 2024, Efruxifermin and Pegozafermin entered Phase 3 clinical trials. Importantly, both FGF21 analogues reduced hepatic fat content and supported fibrosis resolution. The biological functions of FGF21 and the current status of FGF21 based therapies in clinical trials was the subject of recent reviews.^3^, ^4^ Briefly, FGF21 codes for a peptide hormone which is synthesised in the liver, adipose and other extrahepatic tissue in response to metabolic stress. It signals through FGF‐receptors with β‐Klotho functioning as co‐receptor. We and others demonstrated marked FGF21 induction in fat‐laden primary human hepatocyte cultures,^5^ and in MASLD patients, the FGF21 protein in the circulation is significantly increased.^6^ Despite intense research, the molecular functions of FGF21 in MASLD remain uncertain, and a systematic study of hepatic FGF21 expression in a large cohort of patients has not been performed.

Strikingly, hepatic FGF21 expression varied considerably among patients. We identified patients with increased (*N* = 117, 30%), decreased (*N* = 159, 40%) or unchanged FGF21 expression (*N* = 120, 30%), and by comparing the genomes of the three cohorts, we decoded molecular circuitries of FGF21 and identified potentially new therapeutic targets in MASLD.

Overall, we investigated hepatic expression of FGF21 in regards to gender and clinical characteristics, and considered histological scores of NAS, steatosis, ballooning, lobular inflammation and fibrosis. Second, through comparative genomics, we searched for molecular therapeutic targets of FGF21 in relation to metabolic dysfunction of the liver. We focused on autophagy and energy sensing, glucose metabolism and mitochondrial respiration, lipid metabolism, inflammation and fibrosis, and therefore, we gained deep insight into FGF21 mode of action (MoA). Together, our study provides a molecular rationale of why some patients may not benefit from FGF21 based therapies.

## 2 MATERIALS AND METHODS

### 2.1 Human liver biopsy genomic data

As outlined in our recent publication^2^ (Tang and Borlak, 2024), we retrieved data sets from the public repository (https://www.ncbi.nlm.nih.gov/geo/) for MIAME‐compliant data submissions, and next to our own data of 22 cases, we considered GSE48452, GSE89632, GSE135251 and GSE130970 data sets totalling 328 patients. These were classified according to the NAS disease activity score and fibrosis grades. The NAS score measures disease activity by considering grades of steatosis (score 0–3), lobular inflammation (0–3) and hepatocyte ballooning (0–2) and ranges from 0 to 8. Further details are described in Ref. 7. Additionally, 15 patients were scored by Brunt grade 2 and 3 (GSE37031 and GSE63067), and 53 patients according to the SAF and Matteoni classification (GSE126848 and GSE33814, see Tables S1 and S2).

Finally, 396 MASLD patients entered the study which we compared with 66 healthy controls (Table S3). The methods of data processing and an identification of differential expressed genes (DEGs) are the same as described previously.^2^ We only considered DEGs with a false discovery rate corrected *p* value < .05 and a fold change (FC) ≥1.5 of DEGs.

### 2.2 Functional enrichment analysis

We performed Gene Set Enrichment Analysis (GSEA) analysis by R computing to search for unbiased enriched GO terms and KEGG pathways. We evaluated enriched terms with a *p* value < .05 and an absolute value of normalised enrichment score (NES) > 1 for DEGs (Table S8).

### 2.3 Major differences in MASLD‐dependent hepatic FGF21 expression

**FIGURE 1:**
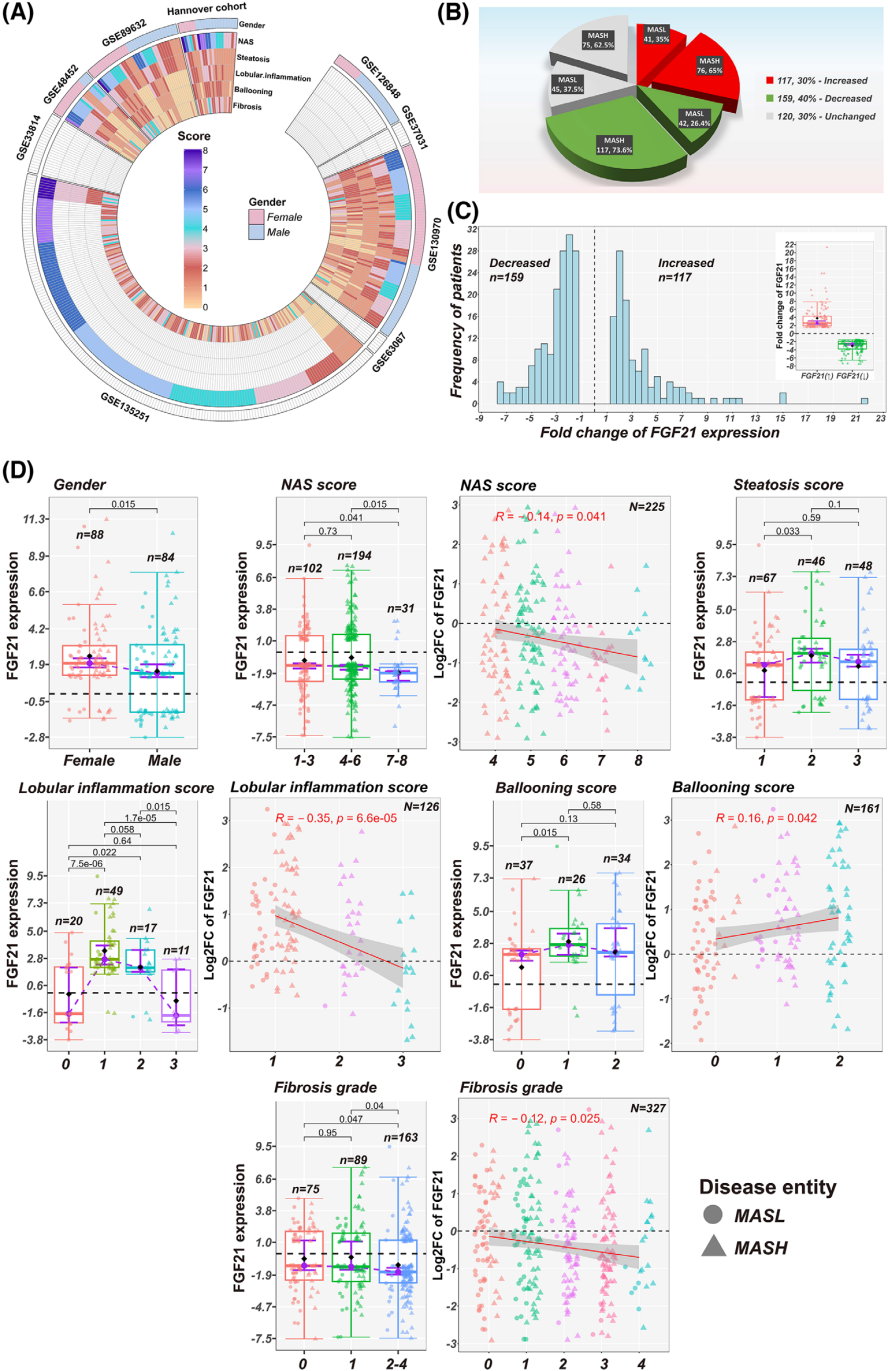
Summary of MASLD‐patient characteristics. (A) The circle heat map shows the NAS disease activity scores, fibrosis grades and sex of MASLD patients across nine independent studies. (B) Distribution of MASL and MASH cases in patients with increased, decreased or unchanged FGF21 expression across 396 MASLD patients. (C) The frequency of MASLD patients in relation to hepatic FGF21 expression status. (D) Box‐plots representation of (1) sex‐specific changes in FGF21 expression, (2) FGF21 expression changes in relation to NAS activity, (3) steatosis, (4) lobular inflammation, (5) ballooning scores and (6) fibrosis grades. The data are fold‐changes relative to healthy controls. *p* Values were calculated with the Wilcoxon rank‐sum test in pair‐wise comparisons. Spearman correlations were computed in R. MASLD, metabolic dysfunction‐associated steatotic liver disease; MASL, metabolic dysfunction‐associated steatotic liver; MASH, metabolic dysfunction‐associated steatohepatitis; NAS, NAFLD/MASLD activity score; FGF21, fibroblast growth factor 21.

We evaluated 396 MASLD patients and grouped them according to hepatic FGF21 expression into increased (FGF21‐up) or decreased (FGF21‐down) cases, and performed comparative genomic analysis by comparing it with healthy controls (Figure 1). This revealed significant DEGs in patients with either increased or decreased FGF21‐expression. Subsequently, we performed pair‐wise comparison between these cases and also evaluated DEGs among cases with no change in FGF21 expression. To identify significant differences, we applied a Wilcoxon–Mann–Whitney test.

**FIGURE 2:**
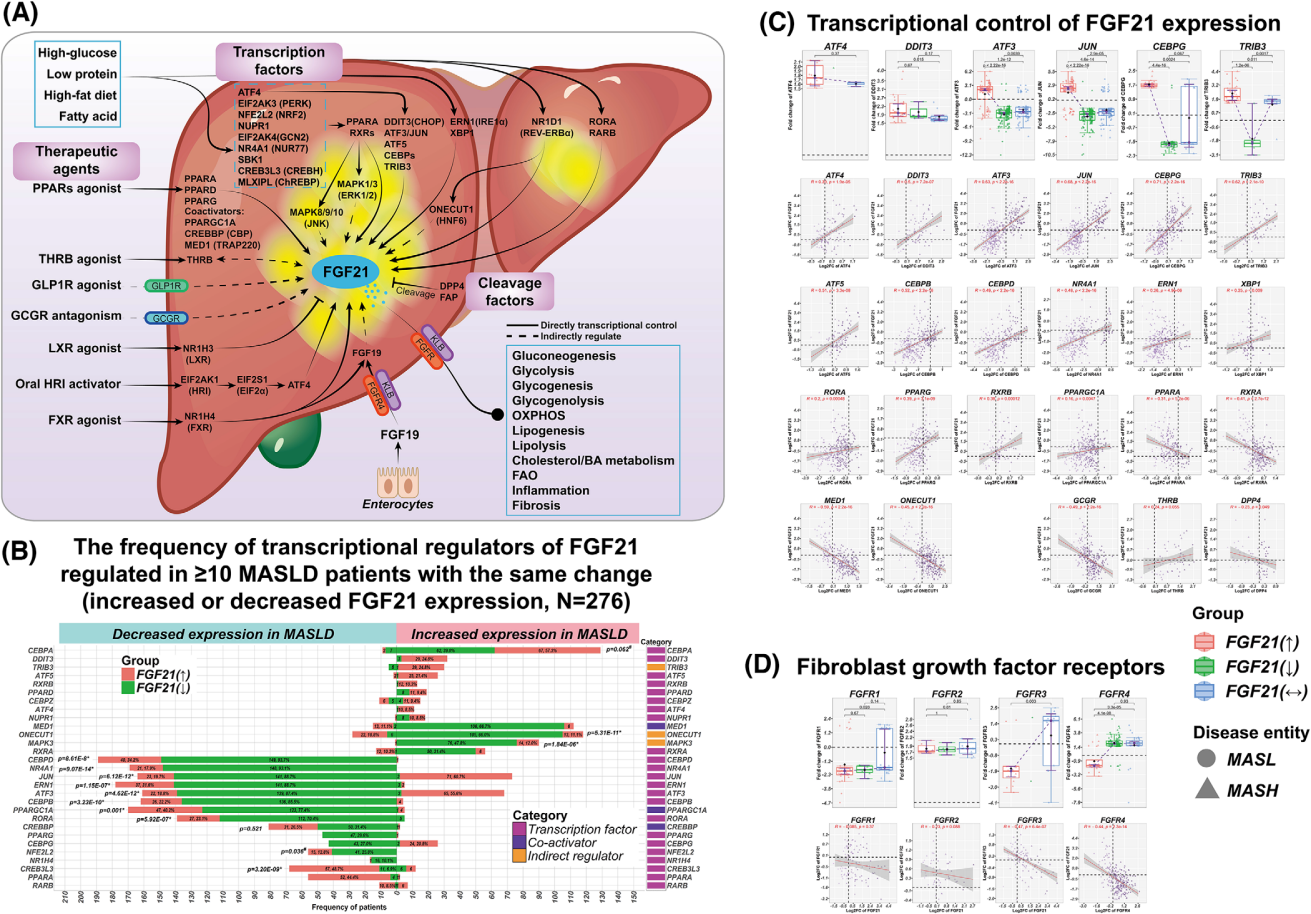
Transcriptional control of FGF21 and its receptors in MASLD patients. (A) Summary of the various effectors in the control of FGF21 expression. (B) The bar‐charts represent the frequency of MASLD patients with either increased or decreased transcriptional regulators of FGF21. Only regulators where regulation occurred in ≥10 MASLD patients with increased or decreased FGF21 expression were considered. The proportions of TFs that differed between patients with increased and decreased FGF21 expression were analysed using the G‐test with William's correction, with significance set at *α* = .025 (corrected *p* value for a two‐way comparison, *p* = .05/2 = .025). *p < .025, #p as a significant borderline. (C) Box‐plots representation of changes in transcription factors expression according to the FGF21‐expression status, that is, increased, decreased or unchanged. Spearman correlations between TFs and FGF21 expression. (D) Box‐plot representation of changes in FGF‐receptor expression according to the FGF21‐expression status. The data are fold‐changes relative to healthy controls. *p* Values were calculated in pair‐wise comparisons with the Wilcoxon rank‐sum test. Spearman correlations were computed in R. TFs, transcription factors.

Apart from statistical considerations, we examined the plausibility for DEGs to be FGF21 targets. First, we analysed the regulation of transcription factors (TFs) which control FGF21 expression among cases with increased or decreased FGF21 expression, and requested the same regulation in ≥10 MASLD patients for further consideration (Figure 2B). Second, to define FGF21 targets, we considered published data. Thus, we examined a large set of experimentally validated DEGs based on MASLD‐animal models on a high‐fat diet (HFD) following treatment with FGF21 synthetic analogues. Third, we evaluated FGF21 targets in FGF21 transgenic as well as FGF21‐knockout (KO) mice given a HFD. Fourth, we analysed original microarray and RNAseq data and searched for commonly regulated genes between MASLD patients and animal models to independently validate FGF21 target genes. Note, Figure S1 shows the workflow and Figure S2D shows the comparison between MASLD patient and animal data. Eventually, we compiled a list of 188 validated FGF21 targets of which 146 were regulated in MASLD patients (see Table S4).

### 2.4 Cytokines and cell markers

We interrogated KEGG pathways (https://www.genome.jp/kegg/pathway.html) to define genes coding for cytokines/chemokines and their receptors and salvaged information reported in Refs. ^8^ and ^9^ (Table S5). Moreover, we retrieved information from the CellMarker 2.0 (http://bio‐bigdata.hrbmu.edu.cn/CellMarker), the sctype (https://sctype.app/database.php) and the GepLiver database (www.gepliver.org), and used single cell RNAseq data^10^ to investigate MASLD‐related changes of resident liver cells (Table S6). Furthermore, we considered immune cell marker genes from LM22^11^ (Table S6), and performed GSEA analysis by R computing to search for enriched immune cell types. We evaluated enriched immune cell marker genes with a *p* value < .05 and a NES > 1 for DEGs (Table S9).

### 2.5 Extracellular matrix coding genes/matrisome

We retrieved information from the public domain^12^, ^13^ and the Matrisome Project database (http://matrisomeproject.mit.edu; accessed on 07032024), and compile information for 1027 extracellular matrix (ECM) coding genes in Table S7. These were categorised into core matrisome, that is, glycoproteins, proteoglycans (PGs) and collagens (COLs) and matrisome‐associated genes, that is, ECM‐affiliated, ECM regulators and secreted factors.

### 2.6 Statistical testing

We used R‐language for statistical computations and applied the “Shapiro–Wilk” test to examine normal distribution. We used the G‐test with William's correction for categorical variables and considered statistical significance at *α* = .017. Specifically, we corrected the *p* value for the three‐way comparison (*p* = .05/3 = .017), and for non‐normal distributed data, we used the “Kruskal–Wallis rank sum” and “Wilcoxon rank sum” test (Mann–Whitney *U* test). Conversely, we applied ANOVA and *t*‐test with equal variances for normal distributed data and computed Spearman correlations and robust fitting of linear models in R.

The data were visualised as box‐plots with interquartile range and 95% confidence interval for a population median between the *j*th and *k*th observation in ordered sample data.^14^, ^15^ A *p* value of  <.05 is considered significant.

## 3 RESULTS

### 3.1 | Patient demographics

Table 1 summarises the basic characteristics of 396 liver biopsy proven MASLD cases, and Figure 1A lists the clinical information of nine independent studies with an emphasis on NAS disease activity scores, fibrosis grades and gender. The average age is 47.6 years and the study is balanced for gender, that is, 89 females and 86 males. However, for 221 patients the gender was not reported. The NAS score for 328 patients ranged between 1 to 8, and included 102 metabolic dysfunction‐associated steatotic liver (MASL) (NAS 1–3) and 226 MASH (NAS 4–8) cases. Among MASH, 37.6% were scored NAS ≥ 6. The paradigm of NAS is based on the sum of scores related to steatosis (score 0–3), inflammation (score 0–3) and hepatocyte ballooning (score 0–2). The summary score for 328 patients was reported in Table S2, and we obtained individual scores related to steatosis, inflammation and hepatocyte ballooning for 162 cases. Most patients presented higher steatosis scores, and for lobular inflammation, 50 and 28%, respectively were scored 1 or 2–3. In regards to hepatocyte ballooning about 32 and 30% of cases were scored 1 and 2 and >82% (84 out of 102) of MASL cases were graded *F* = 0 and *F* = 1. Notwithstanding, there were 7, 6 and 5 MASL cases, respectively with fibrosis grades 2, 3 and 4 (Table S2). Equally, 65% of MASH patients presented histological grades *F* = 2 to *F* = 4, yet 25 and 55 cases were graded *F* = 0 and *F* = 1.

**TABLE 1:** The basic patient characteristics of 396 liver biopsy proven MASLD cases.

			MASLD patients (*N* = 396)					*p* Value (Wilcoxon‐/‐test for pair‐wise comparison)		
Characteristics	Overall (*N* = 396)		FGF21 (↑) (*N* = 117)	FGF21 (↓) (*N* = 159)	FGF21(↔) (*N* = 120)	*p* Value (*G*‐test and William's correction for 3‐groups)	*p* Value (Kruskal–Wallis–/ANOVA‐test for 3‐groups)	FGF21(↑) vs. FGF21(↓)	FGF21(↑) vs. FGF21(↔)	FGF21(↓) vs. FGF21(↔)
**Age; year (*N* = 123)**										
Mean ± SD	47.60 ± 11.55 (*n* = 123)		48.68 ± 12.06 (*n* = 68)	44 ± 10.57 (*n* = 10)	46.78 ± 10.95 (*n* = 45)		*p* = .412	*p* = .223	*p* = .388	*p* = .468
Median (CI95)	48 (45–51)		48.5 (44.5–53)	48 (36–53)	47 (44–49)					
**BMI; kg/m^2^ (*N* = 71)**										
Mean ± SD	33.85 ± 11.21 (*n* = 71)		34.61 ± 10.74 (*n* = 22)	30.76 ± 5.74 (*n* = 9)	34.12 ± 12.41 (*n* = 40)					
Median (CI95)	30.5 (28.86–32.32)		31.995 (28.86–33.43)	29.64 (23.54–35.59)	29.825 (27.385–32.365)		*p* = .712	*p* = .528	*p* = .457	*p* = .99
**Gender: cases with gender information (*N* = 175)**										
F/M	89/86					*p* = .821^a^				
Female	51% (*n* = 89)		59% (*n* = 60)	23% (*n* = 3)	43% (*n* = 26)	*p* = 2.84E−04*				
Male	49% (*n* = 86)		41% (*n* = 41)	77% (*n* = 10)	57% (*n* = 35)	*p* = .004*				
**NAS; score (*N* = 328)**										
1–3	31% (*n* = 102)		31% (*n* = 25)	27% (*n* = 41)	37% (*n* = 36)	*p* = .454				
4–6	59% (*n* = 195)		65% (*n* = 53)	57% (*n* = 86)	58% (*n* = 56)	*p* = .732				
7–8	10% (*n* = 31)		4% (*n* = 3)	16% (*n* = 23)	5% (*n* = 5)	*p* = .008*				
Mean ± SD	4.34 ± 1.73 (*n* = 328)		4.22 ± 1.47 (*n* = 81)	4.59 ± 1.78 (*n* = 150)	4.06 ± 1.82 (*n* = 97)					
Median (CI95)	4 (4–5)		4 (4–5)	5 (4–5)	4 (4–5)		*p* = .071^#^	*p* = .092^#^	*p* = .741	*p* = .039*
**Steatosis; score (*N* = 162)**										
1	41% (*n* = 67)		31% (*n* = 23)	39% (*n* = 9)	55% (*n* = 35)	*p* = .03^#^				
2	29% (*n* = 47)		38% (*n* = 29)	22% (*n* = 5)	20% (*n* = 13)	*p* = .032^#^				
3	30% (*n* = 48)		31% (*n* = 23)	39% (*n* = 9)	25% (*n* = 16)	*p* = .214				
Mean ± SD	1.88 ± 0.84 (*n* = 162)		2 ± 0.79 (*n* = 75)	2 ± 0.9 (*n* = 23)	1.7 ± 0.85 (*n* = 64)					
Median (CI95)	2 (2–2)		2 (2–2)	2 (1–3)	1 (1–2)		*p* = .072^#^	*p* = 1	*p* = .026*	*p* = .164
**Lobular inflammation; score (*N* = 162)**										
0	22% (*n* = 35)		12% (*n* = 9)	48% (*n* = 11)	24% (*n* = 15)	*p* = 6.72E−06*				
1	50% (*n* = 81)		63% (*n* = 47)	9% (*n* = 2)	50% (*n* = 32)	*p* = .222^&^				
2	18% (*n* = 29)		20% (*n* = 15)	13 (*n* = 3)	17% (*n* = 11)	*p* = .476				
3	10% (*n* = 17)		5% (*n* = 4)	30% (*n* = 7)	9% (*n* = 6)	*p* = 9.95E−06*				
Mean ± SD	1.17 ± 0.89 (*n* = 162)		1.19 ± 0.71 (*n* = 75)	1.26 ± 1.36 (*n* = 23)	1.13 ± 0.88 (*n* = 64)					
Median (CI95)	1 (1–1)		1 (1–1)	1 (0–2)	1 (1–1)		*p* = .764	*p* = .677	*p* = .464	*p* = .948
**Ballooning; score (*N* = 162)**										
0	38% (*n* = 62)		33% (*n* = 25)	52% (*n* = 12)	39% (*n* = 25)	*p* = .108				
1	32% (*n* = 52)		32% (*n* = 24)	9% (*n* = 2)	41% (*n* = 26)	*p* = .293^&^				
2	30% (*n* = 48)		35% (*n* = 26)	39% (*n* = 9)	20% (*n* = 13)	*p* = .033^#^				
Mean ± SD	0.91 ± 0.82 (*n* = 162)		1.01 ± 0.83 (*n* = 75)	0.87 ± 0.97 (*n* = 23)	0.81 ± 0.75 (*n* = 64)					
Median (CI95)	1 (1–1)		1 (1–1)	0 (0–2)	1 (0–1)		*p* = .355	*p* = .475	*p* = .152	*p* = .943
**Fibrosis; grade (*N* = 328)**										
0	23% (*n* = 75)		28% (*n* = 23)	19% (*n* = 29)	24% (*n* = 23)	*p* = .422				
1	27% (*n* = 89)		32% (*n* = 26)	21% (*n* = 31)	33% (*n* = 32)	*p* = .198				
2–4	50% (*n* = 164)		40% (*n* = 32)	60% (*n* = 90)	43% (*n* = 42)	*p* = .095^#^				
Mean ± SD	1.64 ± 1.25 (*n* = 328)		1.4 ± 1.21 (*n* = 81)	1.85 ± 1.25 (*n* = 150)	1.51 ± 1.23 (*n* = 97)					
Median (CI95)	1.5 (1–2)		1 (1–2)	2 (2–2)	1 (1–2)		*p* = .012*	*p* = .008*	*p* = .544	*p* = .027*

Abbreviations: BMI, body mass index; CI95, confidence interval at 95%; FGF21, fibroblast growth factor 21; MASLD, metabolic dysfunction‐associated steatotic liver disease; NAS, NAFLD/MASLD activity score; SD, standard deviation.

^a^ The distribution of cases based on sex is balanced.

* The *p* value refers to a comparison between the three groups. We used the *G*‐test with William's correction for categorical variables and considered significance at *α* = .017, that is, we corrected the *p* value for the three‐way comparison (*p *= .05/3 = .017). For continuous variables, we used the Kruska–Wallis rank sum test and Wilcoxon rank sum test for non‐normal distribution data, ANOVA and *t* test for normal distribution data with equal variances, and a *p* value < .05 was considered statistically significant. ^#^: border‐line significant *p* value. ^&^: *p* value for FGF21(↑) vs. FGF21(↔).

### 3.2 Major differences in MASLD‐dependent hepatic FGF21 expression

We examined hepatic FGF21 expression across 396 patients (Figure 1A) and compared the results with *N* = 66 healthy controls. This defined *N* = 117, *N* = 159 and *N* = 120 cases, respectively with increased (range 1.5 to 21.3‐fold‐change), decreased (range −1.5 to −7.5‐fold‐change) or unchanged FGF21 (Figure 1B,C). We grouped patients according to the FGF21 status, and in MASLD patients with increased FGF21 expression 65% were MASH cases as compared with 73.6% in patients with decreased FGF21 expression. Although the proportion of MASH cases is greater among patients with decreased FGF21 expression it did not reach statistical significance (*p* = .606 based on the *G*‐test). We obtained similar results for patients with unchanged FGF21 expression, that is, 62.5% are MASH cases.

FGF21 expression is sex dependent and was significantly increased among females (Table 1). Furthermore, FGF21 expression was inversely related to NAS 4–8 and significant lower in NAS 7–8 (Figure 1D). FGF21 was loosely related to steatosis grades, and although for lobular inflammation scores 1 and 2 FGF21 expression increased (Figure 1D, box‐plots), we computed and inverse relationship. Therefore, lower FGF21 is associated with higher scores of inflammation. The relationship between FGF21 and hepatocyte ballooning was positive, and although FGF21 expression did not differ between *F* = 0 and *F* = 1, it was significant lower in *F* = 2–4. The negative relationship between FGF21 and fibrosis grades was significant (Figure 1D).

Next, we addressed the question whether the frequency of a histological score differed between these cohorts (Table 1) and used the *G*‐test with William's correction for statistical testing. Strikingly, the proportion of cases with NAS score 7–8 was four times greater in patients with decreased FGF21 expression. Thus, lower FGF21 expression was more frequent among severe MASLD cases. In regards to steatosis, the frequency of score 1 was about 1.8‐fold higher in patients with unchanged FGF21 expression. Similar, the proportion of score 2 was nearly twofold higher in cases with increased FGF21 expression. Regarding lobular inflammation and patients with increased FGF21 expression, the frequency of score 3 was highly significantly reduced, that is, sixfold and twofold, respectively when compared with cases with decreased or unchanged FGF21.

Furthermore, an inverse relationship exists between FGF21 and fibrosis grades (Figure 1D). However, the three‐way comparison (Table 1) yielded borderline significance for fibrosis grades *F* = 2–4 only. We performed the Kruskal–Wallis test, and based on median scores, the results are borderline significant for NAS and steatosis; however, the data were truly significant for fibrosis (Table 1). Finally, we performed a Wilcoxon rank sum‐test, and in the comparison between increased versus decreased FGF21 expression, NAS is borderline significant but highly significant for fibrosis. NAS and fibrosis grades differed significantly when decreased versus unchanged FGF21 cases were compared.

### 3.3 Transcriptional control of FGF21 in MASLD

Figure 2A summarises the various effectors in the control of FGF21 expression. Next to nutritional factors (starvation/overnutrition) and hormones, we highlight TFs, therapeutic agents and emphasise the functions of FGF21 in metabolism, inflammation and fibrosis. The control of FGF21 gene expression depends on TFs interacting with its regulatory sequence elements. Therefore, we evaluated regulation of 64 TFs and coactivators known to control FGF21 transcription. Shown in Figures 2B and S2A is the frequency of patients with increased or decreased transcriptional regulators of FGF21. Specifically, ATF4 and CHOP/DDIT3 stimulate FGF21 transcription and were mostly up‐regulated in patients with increased FGF21 expression.^16^ Outstandingly, ATF3 and c‐Jun/JUN were specifically up‐regulated in patients with increased FGF21 expression (Figure 2C). Accordingly, ATF3 binds to an amino acid response element, whereas heterodimeric ATF3/JUN binds to the cyclic AMP response element (CRE) site of the FGF21 promoter.^17^, ^18^ ATF3, CHOP/DDIT3, TRIB3 and CEBPG are targets of ATF4, and all were up‐regulated in patients with increased FGF21 expression^19^ (Figure 2C). In fact, ATF4 is a master regulator of FGF21.

To define significance, we set a threshold of ≥10 MASLD cases that must show the same change in TF regulation, and in this way, we identified 29 TFs and co‐activators which directly influenced FGF21 expression (Figure 2B). Expression of the TFs ATF3, JUN, CEBPB, CEBPD, CEBPG, ERN1, NRF2/NFE2L2, NR1H4, NR4A1, RORA, PPARγ/ PPARG and PPARGC1A was commonly down‐regulated in patients with decreased FGF21 expression. Conversely, ATF4, DDIT3, ATF3, JUN, ATF5, CEBPA, CEBPG, CEBPZ, NUPR1, PPARδ/PPARD and RXRB was mostly up‐regulated in patients with increased FGF21 expression (Figure 2B). Collectively, we obtained mechanistic insights as to why patients show increased or decreased FGF21 expression. Unexpectedly, CREBH/CREB3L3, PPARα/PPARA and RARβ/RARB, which also stimulate FGF21‐transcription, were mainly down‐regulated in patients with increased FGF21 expression. Moreover, RXRA, TRAP220/MED1 and HNF6/ONECUT1 were primarily up‐regulated in MASLD cases with decreased FGF21 expression (Figure 2B). Note, the tethering of Rev‐erbα/NR1D1 to HNF6/ONECUT1 binding sites blocks expression of lipid metabolism genes^20^ and was mainly decreased in MASLD patients (Figure S2A).

Furthermore, we observed positive correlations between FGF21 and the expression of ATF3, ATF4, ATF5, JUN, CHOP/DDIT3, CEBPG, CEBPB, CEBPD, NR4A1, ERN1, XBP1, RORA, PPARG and RXRB (Figure 2C). Conversely, the TFs PPARA, RXRA, MED1 and ONECUT1 were inversely related to FGF21 expression. Additionally, expression of the glucagon receptor (GCGR) was inversely related to FGF21 expression but thyroid hormone receptor β was positively correlated with FGF21 expression. DPP4, which inactivates FGF21 through cleavage, was inversely related to FGF21 expression (Figure 2C). Last, we observed negative correlations between FGF21 and its receptors FGFR3 and FGFR4 while the correlations for FGFR1 and FGFR2 did not reach statistical significance (Figure 2D). Together, we observed clear differences in the control of FGF21 transcription in MASLD cases with increased or decreased FGF21 expression.

Apart from transcriptional regulators of FGF21, we searched for FGF21 target genes. First, we collected published data and summarise the information in Table S4. This defined a master list of 188 experimentally validated targets, and the majority are derived from MASLD animal models treated with FGF21 synthetic analogues. Second, we independently validated FGF21 target genes by analysing original genomic data sets of FGF21‐transgenic (*N* = 3, GSE39313) and FGF21‐KO mice (*N* = 14, GSE58062; GSE158653; GSE122167) in addition to mice on a HFD treated with FGF21 analogues (*N* = 20, GSE210683, see workflow Figure S1). Finally, 87 genes or 46.3% were in common, and therefore nearly half of the FGF21 targets were independently validated (Figure S2D). Third, we assessed the regulation of FGF21 targets in 276 MASLD patients whose FGF21 expression was significantly changed. This defined 146 out of 188 DEGs or >77% as regulated in MASLD (Figure S2D). Therefore, we obtained solid evidence for FGF21 target/responsive DEGs regulated in MASLD patients and provide more detailed information in Figure S2D. Here, we compared DEGs of MASLD patients with increased FGF21 expression to DEGs identified in transgenic and DIO mouse models treated with FGF21 analogues. We also compared MASLD patients with decreased FGF21 expression to DEGs in FGF21‐KO mice.

### 3.4 FGF21 signalling in energy sensing and autophagy

**FIGURE 3:**
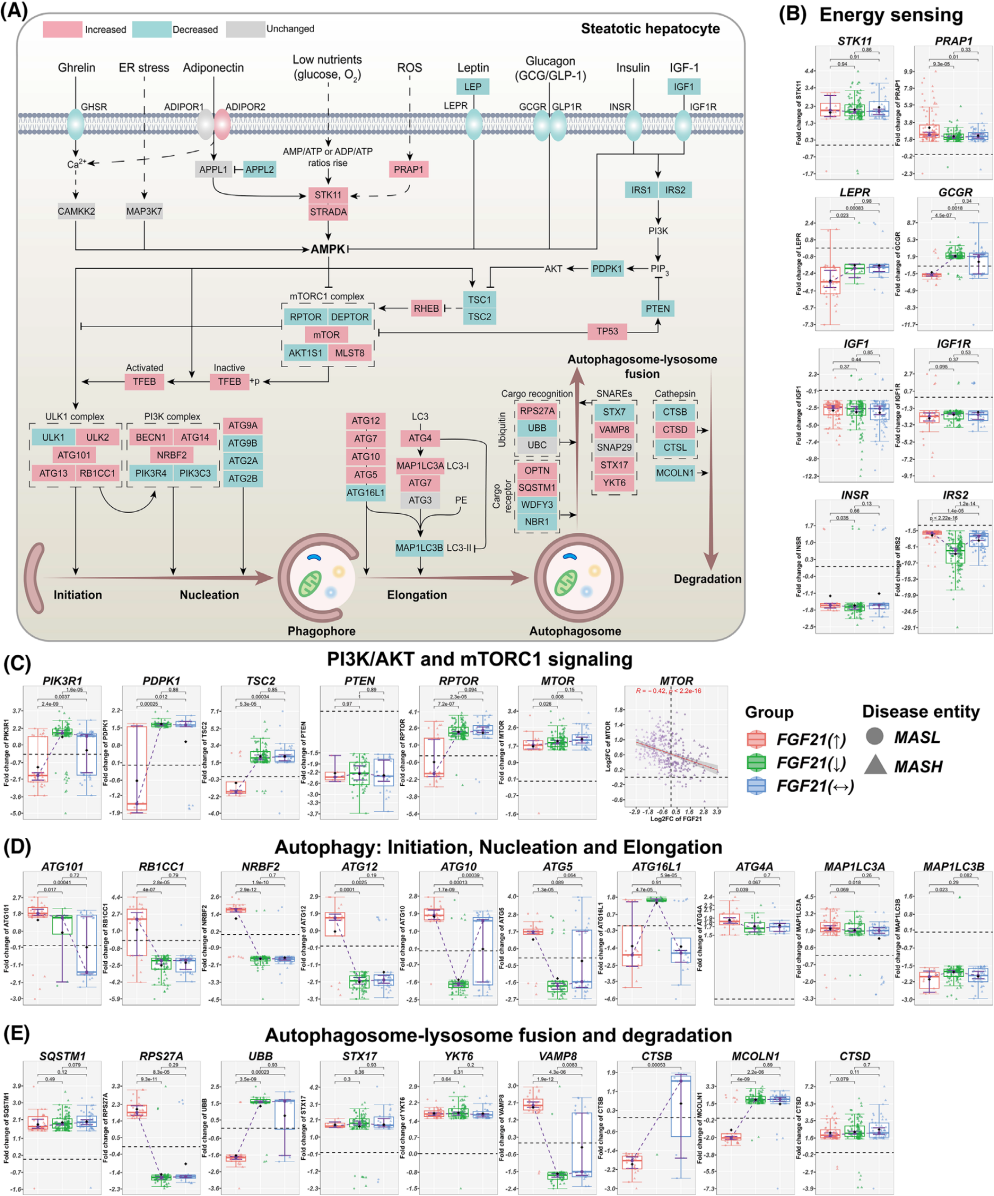
Energy sensing and autophagy signalling in MASLD patients according to the hepatic FGF21 expression status. (A) DEGs coding for energy sensing and autophagy in MASLD patients with increased FGF21 expression. Red colour represent increased, green decreased and grey unchanged DEGs expression in MASLD patients with increased FGF21. (B) DEGs coding for energy sensing. (C) DEGs encoding PI3K/AKT and mTORC1 signalling. (D) DEGs coding for initiation, nucleation and elongation of autophagy. (E) DEGs encoding autophagosome–lysosome fusion and degradation. The box‐plots represent changes in DEGs according to the patients FGF21‐expression status, that is, increased, decreased or unchanged. Red coloured box‐plots represent increased, green decreased and grey unchanged FGF21 expression. *p* Values were calculated in pair‐wise comparisons with the Wilcoxon rank‐sum test. Spearman correlations were computed in R. ER, endoplasmic reticulum; ROS, reactive oxygen species; AMP, ADP and ATP, adenosine monophosphate, diphosphate and triphosphate, respectively; AMPK, adenosine monophosphate‐activated protein kinase; LC3, microtubule‐associated protein 1A/1B‐light chain 3; LC3‐I, a cytosolic form of LC3; LC3‐II, LC3‐phosphatidylethanolamine conjugate; PE, phosphatidylethanolamine; SNAREs, soluble N‐ethylmaleimide‐sensitive factor attachment protein receptors.

Figure 3 summarises DEGs linked to energy sensing and autophagy. We focused on key molecules in PI3K/AKT signalling, autophagy/cell death, autophagosome–lysosome fusions and its degradation. The box‐plots represent FC of DEGs, and we compared patients with increased, decreased or unchanged FGF21 expression. Except for adiponectin receptor 2, which was significantly up‐regulated, the ghrelin‐, leptin‐, glucagon‐, insulin‐ and IGF1‐receptors were repressed, as were insulin receptor substrates 1 and 2 (Figures 3B and S3A). The relationship between FGF21 and expression of LEPR, GCGR and INS was negative but positive for INSR and IRS2 (Figure S4). Given that leptin levels are increased in MASLD patients,^21^ and leptin promotes insulin resistance,^22^ we consider the negative relationship between FGF21 and LEPR as highly suggestive for FGF21 to improve insulin sensitivity by dampening leptin signalling. In animal studies, chronic administration of FGF21 reduced insulin plasma levels by >90% in mice on an HFD^23^ and the negative correlation between FGF21 and INS provided confirmatory evidence. Moreover, GCGR was specifically repressed in cases with increased FGF21 expression, and the relationship between FGF21 and GCGR was negative (Figure S4A). Conceivably, FGF21 dampened glucagon signalling to attenuate blood glucose levels.

Among energy sensors, we highlight STK11(= LKB1) and STRADA. Both stimulate AMPK activity while PRAP1 prompted lipid uptake and microsomal triglyceride transfer.^24^ Although these effectors were up‐regulated (Figure 3B), we noted an opposite relationship between FGF21 and PRAP1 and STK11 expression (Figure S4A). Conversely, the correlation between FGF21 and STRADA as well as AMPK isoforms was positive. Importantly, AMPK activates autophagy through direct phosphorylation of the ULK1 complex and inhibits mTORC1 signalling^25^ (Figure 3A).

The specific down‐regulation of PIK3R1 in patients with increased FGF21 expression will improve insulin sensitivity as shown in PIK3R1 knock‐down MASLD animal models.^26^ Furthermore, PDPK1, which phosphorylates AKT, was down‐regulated in patients with increased FGF21 expression. Importantly, phospho‐AKT inhibits autophagy through phosphorylation of beclin‐1/BECN1.^27^ We observed a negative correlation between FGF21 and beclin‐1 expression (Figure S4B). Additionally, AKT phosphorylation of the TSC2/tuberin and TSC1/hamartin complex inhibits mTORC1 signalling and therefore stimulates autophagy.^28^ In MASLD cases with increased FGF21 expression TSC2 was markedly down‐regulated, and the negative correlation between FGF21 and TSC1 or TSC2 was significant.

Depicted in Figure 3A are the core components of the mTORC1 complex, that is, the regulatory‐associated protein (RPTOR) and AKT1S1 which were specifically repressed in cases with increased FGF21 expression. Nonetheless, DEPTOR was repressed independent of FGF21. mTORC1 signalling inhibits autophagy.^29^ Importantly, we observed a negative correlation between FGF21 and mTOR expression (Figure 3C), and several lines of evidence support the notion of FGF21 to stimulate autophagy. This includes members of the ULK1 and PI3K complex, that is, ATG101, RB1CC1 and NRBF2 which stimulate autophagy and were markedly up‐regulated in patients with increased FGF21 expression (Figure 3D). However, ATG16L1 and MAP1LC3B/LC3‐II were repressed; yet, MAP1LC3A/LC3‐I was up‐regulated and functions as a critical effector of autophagosome biogenesis (Figure 3D). The positive correlations between FGF21 and molecules of an initiation, nucleation and elongation phase of the phagophore were impressive (Figure S4C). The final step in autophagy involves lysosome fusion and degradation. Strikingly, RPS27A was highly up‐regulated in patients with increased FGF21 expression (Figure 3E), and independent of its ribosomal function, RPS27A inhibits MDM2‐mediated p53 ubiquitination/degradation which leads to p53‐dependent cell cycle arrest.^30^ Given the link between MASLD and hepatocellular carcinoma (HCC), this is an important finding.

Irrespective of FGF21 expression, the cargo receptor optineurin and the scaffolding/adaptor protein sequestosome were up‐regulated (Figure 3E). The fusion of the autophagosome with lysosomes is facilitated by soluble N‐ethylmaleimide‐sensitive factor attachment protein receptor (SNARE) proteins, and we observed marked up‐regulation of VAMP8 in patients with increased FGF21 expression. VAMP8 is essential for autophagosome–lysosome fusion. Furthermore, we observed up‐regulation of syntaxin 17 and the V‐SNARE homolog YKT6 but their expression appeared to be independent of FGF21 (Figure 3E). Notwithstanding, cathepsin B and the lysosomal calcium channel MCOLN1 were markedly repressed in MASLD cases with increased FGF21 expression. Together, FGF21 stimulated different phases of autophagy^31^ (Figures 3E and S4D).

### 3.5 Glucose homeostasis and mitochondrial respiration

**FIGURE 4:**
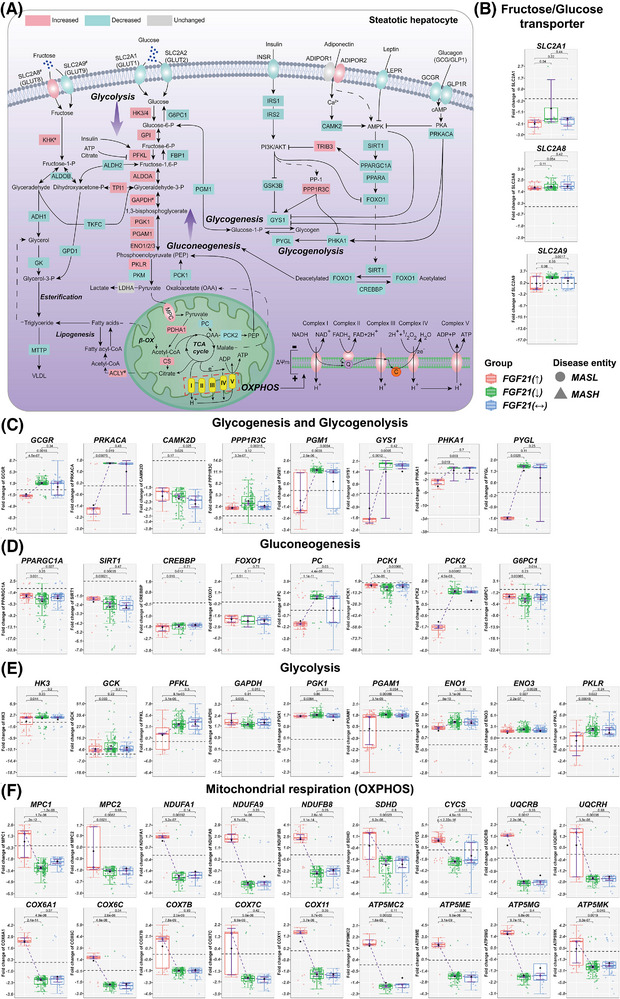
Glucose metabolism and mitochondrial respiration in MASLD patients according to the hepatic FGF21 expression status. (A) DEGs coding for glycogenesis, glycogenolysis, glycolysis/OXPHOS and gluconeogenesis in MASLD patients with increased FGF21 expression. Red colour represent increased, green decreased and grey unchanged DEGs expression in MASLD patients with increased FGF21. (B) DEGs coding for fructose and glucose transporters. (C) DEGs encoding key enzymes in glycogenesis and glycogenolysis. (D) DEGs coding for gluconeogenesis (E) DEGs coding for glycolysis (F) DEGs of mitochondrial respiration. The box‐plots represent changes in DEGs according to the patients FGF21‐expression status, that is, increased, decreased or unchanged. Red coloured box‐plots represent increased, green decreased and grey unchanged FGF21 expression. *p* Values were calculated in pair‐wise comparisons with the Wilcoxon rank‐sum test. β‐OX, β‐oxidation; TCA, citrate cycle; OAA, oxaloacetate; PEP, phosphoenolpyruvate; OXPHOS, oxidative phosphorylation; ΔΨm, the mitochondrial membrane potential.

FGF21 maintains glucose homeostasis in the fasting state, enhances glucose uptake during feeding and functions as an insulin sensitiser.^32^ To understand FGF21 benefits in energy balance, we investigated key enzymes in glycogenesis/glycogenolysis, glycolysis/gluconeogenesis and mitochondrial respiration (Figure 4A) and searched for differences based on the FGF21 expression status (panels 4B–F, Figure S5). Common to all MASLD patients is the repression of the glucose transporters SLC2A1 and SLC2A2 while the fructose transporters SLC2A8 and SLC2A9 were significantly up‐regulated (Figures 4B and S5A).

In cases with increased FGF21 expression, key members of the glycogenesis and glycogenolysis pathway were repressed, and the correlations between FGF21 and members of this pathway were strictly negative (Figure S6A). For instance, PRKACA was repressed, and we determined a negative correlation between FGF21 and PRKACA. PRKACA inactivates glycogen synthase (GYS1) but activates glycogen phosphorylase L (PYGL); and both genes were specifically repressed in patients with increased FGF21 expression (Figure 4C), and their expression is negatively influenced by FGF21 (Figure S6A). Correspondingly, FGF21 repressed glycogen storage and its glucose release which is of therapeutic benefit given the low intracellular but high extracellular glucose levels.

The GCGR was specifically repressed in cases with increased FGF21 expression, and the relationship between FGF21 and GCGR was negative (Figure S4A). Conversely, we observed positive correlation between FGF21 and the insulin receptor (INSR) as well as IRS2 across 365 and 329 MASLD patients (Figure S4A). Together, FGF21 influenced glycogenolysis by modulating GCGR expression.

Regardless of FGF21, CAMK2D expression was repressed in MASLD patients (Figure 4C). This kinase regulates hepatic glucose production by inhibiting FOXO1 nuclear translocation.^33^ FOXO1 is a key regulator of glucose metabolism. Its activity is modified through post‐translational modifications. FOXO1 and its acetylase/deacetylase (CREBBP and SIRT1) were repressed in all MASLD patients (Figure 4D). Notwithstanding, the expression of FOXO1 and SIRT1 were positively influenced by FGF21 (Figure S6B). Remarkably, pyruvate carboxylase (PC) and the mitochondrial phosphoenolpyruvate (PEP) carboxykinase‐2 (PCK2) were specifically repressed in patients with increased FGF21 expression. PC catalyses the conversion of pyruvate to oxaloacetate (OAA), whereas PCK2 converts OAA to PEP. PEP is transported out of mitochondria, and following its dephosphorylation by fructose‐1,6‐bisphosphatase 1 (FBP1), it fuels gluconeogenesis.^34^ However, DEGs coding for gluconeogenesis were repressed, especially PCK1, glucose‐6‐phosphatase (G6PC1) (Figure 4D) and FBP1 (Figure S5C); yet, expression of PCK1 and G6PC1 were positively influenced by FGF21 (Figure S6B). In animals, deficiency of PCK1 promotes MASLD^35^ while deficiency of G6PC1 causes glycogen accumulation.^36^ Collectively, gluconeogenesis is repressed in MASLD, and with the exception of SIRT1, PCK1 and G6PC1, the correlations between FGF21 and PC or PCK2 were strictly negative (Figure S6B). Conversely, DEGs of the glycolytic pathway were up‐regulated (Figure 4E) but their expression was negatively influenced by FGF21 (Figure S6C). Therefore, FGF21 dampened glucose flux in conditions of insulin resistance.

Exceptionally, the mitochondrial pyruvate transporters MPC1 and MPC2 were specifically up‐regulated in patients with increased FGF21 expression (Figure 4F), and the correlation between FGF21 and MPC1/MPC2 was positive (Figure S6D). Pyruvate is metabolised to acetyl‐CoA by the pyruvate dehydrogenase complex (PDH) which was up‐regulated in all MASLD patients (Figure S5D). To initiate lipogenesis, acetyl‐CoA is converted to malonyl‐CoA by acetyl‐CoA carboxylases. However, we determined a negative relationship between FGF21 and acetyl‐CoA carboxylases (Figure S8D). Together, and based on transcriptomic data, FGF21 appears to stimulate pyruvate import into mitochondria but represses its metabolism by PC and PCK2. It therefore attenuates gluconeogenesis and lipogenesis.

Strikingly, FGF21 had profound effects on mitochondrial respiration with oxidative phosphorylation (OXPHOS) almost exclusively up‐regulated in cases with increased FGF21 expression (Figure 4F), and the relationship between FGF21 and key members of the OXPHOS pathway were strictly positive (Figure S6D). Therefore, the data are highly suggestive for FGF21 based therapies to improve adenosine triphosphate (ATP) yield and energy balance.

### 3.6 FGF21 stimulates fatty acid oxidation

**FIGURE 5:**
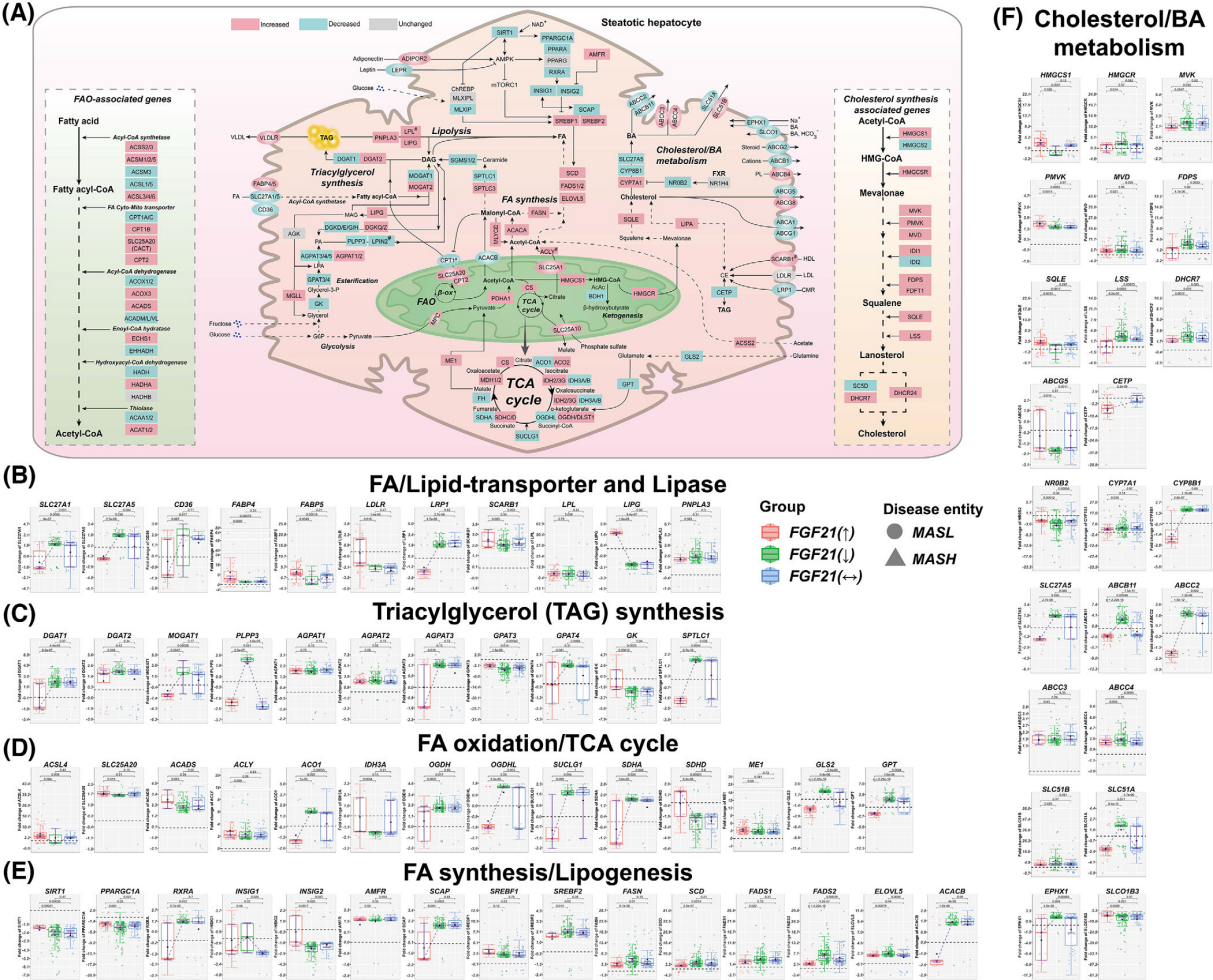
Lipid and cholesterol/bile acid metabolism in MASLD patients according to the hepatic FGF21 expression status. (A) DEGs coding for lipid transport, triacylglyceride synthesis, fatty acid oxidation, TCA cycle, lipogenesis and cholesterol/bile acid metabolism in MASLD patients with increased FGF21 expression. Red colour represent increased, green decreased and grey unchanged DEGs expression in MASLD patients with increased FGF21. (B) DEGs encoding fatty acid/lipid transporters and lipase. (C) DEGs coding for triacylglycerol synthesis. (D) DEGs coding for fatty acid oxidation and TCA cycle. (E) DEGs coding for fatty acid synthesis and lipogenesis. (F) DEGs coding for cholesterol and bile acid synthesis and metabolism. The box‐plots represent changes in DEGs according to the patients FGF21‐expression status, that is, increased, decreased or unchanged. Red coloured box‐plots represent increased, green decreased and grey unchanged FGF21 expression. *p* Values were calculated in pair‐wise comparisons with the Wilcoxon rank‐sum test. FA, fatty acid; FAO, fatty acid β‐oxidation; TAG, triacylglycerol; DAG, diacylglycerol; MAG, monoacylglycerol; PA, phosphatidic acid; LPA, lysophosphatidic acid; G6P, glucose 6‐phosphate; VLDL, very low‐density lipoprotein; HDL, high density lipoprotein; LDL, low density lipoprotein; CMR, chylomicron remnant; CE, cholesterol ester; BA, bile acid; FXR, farnesoid X receptor; PL, phospholipids; AcAc, acetoacetate.

We investigated DEGs coding for key enzymes in fatty acid (FA) transport, its β‐oxidation, triacylglycerol (TAG)/lipid synthesis, and cholesterol metabolism (Figure 5A) and searched for differences according to the FGF21‐expression status (panels 5B–F, Figure S7). Outstandingly, the long‐chain FA transporters SLC27A1, SLC27A5, CD36 as well as the LDL‐receptor related protein (LRP1) were exclusively repressed in patients with increased FGF21 expression, and the relationship between FGF21 and the expression of lipid transporters was negative (Figure S8A). Consequently, the data are highly suggestive for FGF21 to protect from hepatic lipid overload. Conversely, FGF21 and the LDL receptor were positively correlated (Figure S8A). Meanwhile, endothelial lipase was specifically up‐regulated in patients with increased FGF21 expression. It facilitates ApoE acquisition from remnants to stimulate its metabolism,^37^ and this is important, given its highly atherogenic nature. In regards to triglyceride synthesis, especially DGAT1, MOGAT1, AGPAT3 were specifically repressed in patients with increased FGF21 expression while PLPP3 is also repressed in cases with unchanged FGF21 expression (Figure 5C). Together, the data are highly suggestive for FGF21 to suppress hepatic lipid synthesis and except for GPAT3, an inverse relationship between FGF21 and lipogenesis exists (Figure S8B). Furthermore, all MASLD patients showed increased expression of the lipase PNPLA3 to support lipid catabolism (Figure 5B).

FGF21 stimulated FA oxidation (FAO; Figure 5A). We observed mainly positive correlations between FGF21 and several acyl‐CoA synthetases (ACS), that is, ACSM2B, ACSM5, ACSL3‐6 (Figure S8C), whereas the relationship for the short‐chain acyl‐CoA synthetase ACSS2 was negative. Although ACSM3 and ACSL1 support FAO, the relationship with FGF21 was negative. Importantly, the mitochondrial carnitine/acylcarnitine carrier SLC25A20 was significantly up‐regulated, and we observed a positive correlation between FGF21 and SLC25A20 (Figures 5D and S8C). Consequently, more FAs undergo FAO. Additionally, in patients with increased FGF21 expression, the expression of the long‐chain‐fatty‐acid‐CoA ligase 4 (ACSL4) was increased, and independent research showed enhanced arachidonoyl‐CoA synthesis to protect hepatocytes from arachidonic acid stimulated apoptosis.^38^ Note, the expression of the short‐chain acyl‐CoA dehydrogenase (ACADS) was significantly increased in patients with increased FGF21 expression, and ACADS catalyses the first step in FA‐β‐oxidation. While up‐regulation of the ATP‐citrate lyase supports the supply of acetyl‐CoA, its expression was not influenced by FGF21.

In all MASLD patients, PPARα was repressed, and given that PPARα is a key regulator of FGF21, the negative correlation is perplexing (Figure 2C). Additional transcriptional regulators of FAO, that is, PPARGC1A, insulin induced genes INSIG1&INSIG2 and SIRT1 were likewise repressed (Figure 5E). However, their expression was positively correlated with FGF21 (Figure S8D). Therefore, FGF21 stimulated their expression in support of FAO. Among all MASLD patients, expression of key regulators of lipogenesis, that is, SREBF1&2 were increased. In fact, most of the lipogenic genes were up‐regulated in MASLD patients such as FASN, SCD, FADS1&2, ELOVL5 (Figure 5E) in addition to MLYCD, ACACA (Figure S7C). However, the correlations between FGF21 and lipogenic genes were strictly negative (Figure S8D). Of critical importance is the unique repression of acetyl‐CoA carboxylase‐β (ACACB) in patients with increased FGF21 expression. This enzyme catalyses the rate‐limiting step in FA synthesis. Furthermore, the correlations between FGF21 and inhibitors of SREBF1, that is, SIRT1, PPARγ, PPARGC1A and INSIG2 were positive, whereas the relationship between FGF21 and SREBF2 as well as its cleavage‐activating protein SCAP was negative. Together, the data are highly suggestive for FGF21 to repress key enzymes in lipogenesis though different means.

Acetyl‐CoA derived from FAO enters the TCA cycle where it is oxidised to CO2. During this process coenzymes (nicotinamide adenine dinucleotide, flavin adenine dinucleotide) are regenerated for their use in the respiratory chain (Figure 4A). As shown in Figure 5A, six out of eight enzymes were either increased or repressed (additional data are shown in Figure S7). Outstandingly, most TCA coding DEGs were specifically repressed in patients with increased FGF21 expression (Figure 5D), and the correlations with FGF21 were mostly negative (Figure S8C). Since glycolysis was up‐regulated in MASLD patients (Figure 4E), acetyl‐CoA flux derived from pyruvate and FAO is increased. In MASLD mice, insulin resistance promoted hepatic TCA cycle flux which led to mitochondrial dysfunction.^39^ Therefore, repression of TCA coding DEGs by FGF21 is likely beneficial. Note, succinate dehydrogenase A was repressed (Figure 5D) but SDHD was up‐regulated in patients with increased FGF21 expression, and this is of critical importance for the transfer of electrons from the TCA cycle to the respiratory chain. Furthermore, FGF21 and SDHD expression were positively correlated (Figure S8C). There is evidence for glutamine oxidation to maintain the TCA cycle^40^; however, this pathway was specifically repressed in patients with increased FGF21 expression, that is, glutaminase 2 and glutamic‐pyruvic transaminase (Figure 5D). Furthermore, malic enzyme 1 (ME1) was significantly up‐regulated in MASLD patients which catalyses the conversion of malate to pyruvate and generates NADPH, and pyruvate is transported into mitochondria via MPC1 and MPC2 which were up‐regulated in patients with increased FGF21 expression. Together, the data are highly suggestive for FGF21 to stabilise mitochondrial function and to stimulate OXPHOS for an improved energy supply.

Next, we evaluated cholesterol/bile acid (BA) homeostasis (Figure 5A) and except for HMGCS2, IDI2 and SC5D, all genes of the cholesterol biosynthetic pathway were up‐regulated, however, the correlations with FGF21 were mostly negative (Figures 5F and S8E). Furthermore, the cholesterol ester transfer protein (CETP) was significantly down‐regulated in patients with increased FGF21 expression, and the correlation between FGF21 and CETP is negative. Interestingly, CETP inhibition protected animals from fatty liver.^41^ A key enzyme in BA synthesis is CYP7A1 which was mostly up‐regulated in MASLD patients (*N* = 205). This monooxygenase catalyses cholesterol oxidation and BA‐synthesis while its repressor NR0B2/SHP was down‐regulated (*N* = 213). A negative correlation between CYP7A1 and NR0B2/SHP was computed. Strikingly, CYP8B1 was specifically repressed in patients with increased FGF21 expression: It catalyses the production of cholic acid. Given that cholic acid facilitates cholesterol absorption,^42^ its repression is beneficial, and the relationship between FGF21 and CYP8B1 was negative, however, positive for the repressor NR0B2. Remarkably, the BA transporter SLC27A5 was specifically repressed in patients with increased FGF21 expression (Figure 5F) and its cargo, that is, ursodeoxy‐ and deoxycholic acid inhibits cellular uptake of long‐chain FAs to possibly protect hepatocytes from lipid overload.^43^

Finally, we investigated the regulation of BA‐transporters, and irrespective of FGF21, observed up‐regulation of the basolateral exporters, that is, ABCC3, ABCC4 and SLC51B but repression of BA‐uptake into hepatocytes via SLCO1B3. Furthermore, the canalicular export of BAs through ABCB11 and ABCC2, the basolateral export by SLC51A and the Na‐dependent uptake of BAs via EPHX1 were specifically repressed in patients with increased FGF21 expression. Except for SLCO1B3, all transporters were negatively correlated with FGF21 (Figure S8F). The data provide a molecular rationale for increased BA‐plasma levels seen in MASLD patients.^44^

### 3.7 FGF21 and hepatic inflammation

We focused on the regulation of immune cell markers, pro‐inflammatory‐chemokines, ‐cytokines, their receptors and anti‐inflammatory cytokines. First, we evaluated the CIBERSORT gene set termed “LM22” which contains normalised expression values of 547 genes that distinguish 22 different immune cell populations including various T‐cells, B/plasma cells, NK cells and myeloid subsets^11^ (Table S6). Second, we considered immune cell specific markers of the human liver and retrieved marker genes from different databases (see Table S6 for details). Moreover, we selected the top 10 significantly regulated genes based on single‐cell RNA sequencing data of individual cell populations as reported in the human liver atlas project.^10^ Together, we compiled 456 marker genes for different immune cells and subpopulations of T‐cells, B cells, plasma and NK cells and myeloid subsets (see Table S6 for details). Third, we used the LM22 and the human liver atlas gene marker sets independently to interrogate the transcriptomic data of 396 MASLD patients. Based on the GSEA algorithm, we observed enrichment of various marker genes and by implication immune cells (see Table S9 for details).

**FIGURE 6:**
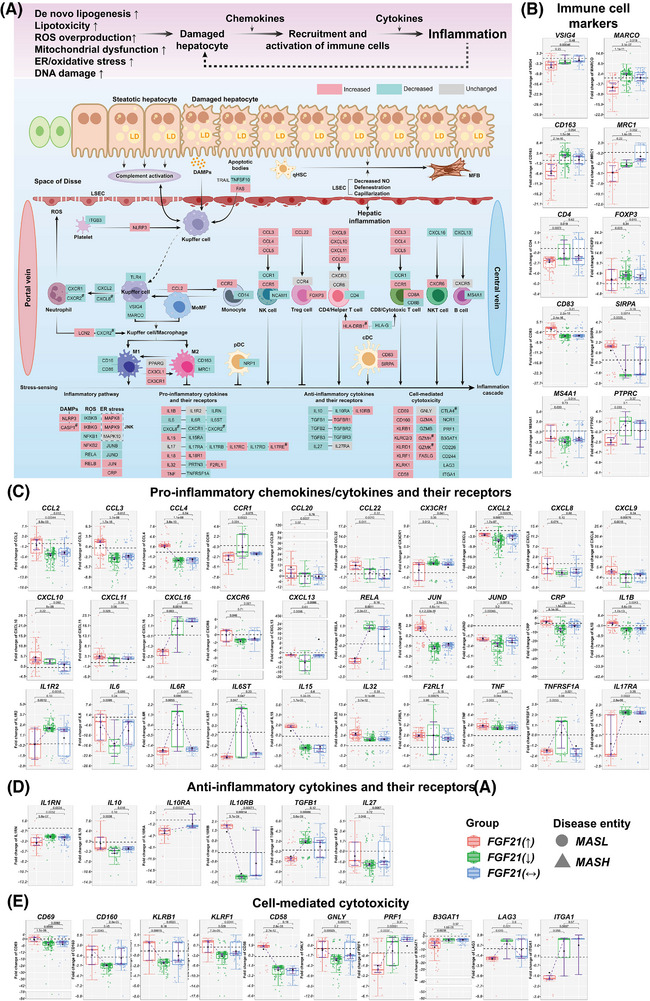
Pro‐ and anti‐inflammatory responses in MASLD patients according to the hepatic FGF21 expression status. (A) DEGs coding for pro‐ and anti‐inflammatory molecules in MASLD patients with increased FGF21 expression. Red colour represent increased, green decreased and grey unchanged DEGs expression in MASLD patients with increased FGF21. (B) DEGs encoding immune cell markers. (C) DEGs coding for pro‐inflammatory chemokines, cytokines and their receptors. (D) DEGs encoding anti‐inflammatory cytokines and their receptors. (E) DEGs coding for cell‐mediated cytotoxicity. The box‐plots represent changes in DEGs according to the patients FGF21‐expression status, that is, increased, decreased or unchanged. Red coloured box‐plots represent increased, green decreased and grey unchanged FGF21 expression. *p* Values were calculated in pair‐wise comparisons with the Wilcoxon rank‐sum test. LSEC, liver sinusoidal endothelial cell; DAMPs, damage‐associated molecular patterns; TRAIL, tumour necrosis factor (TNF)‐related apoptosis‐inducing ligand; MoMF, monocyte‐derived macrophages; M1, M1‐type (classically activated macrophage); M2, M2‐type (alternatively activated macrophage); pDC, plasmacytoid dendritic cell; cDC, conventional dendritic cell (previously called myeloid dendritic cell (mDC)); qHSC, liver resident quiescent hepatic stellate cell; MFB, myofibroblast; NO, nitric oxide.

Figure 6A depicts a simplified scheme of the liver acinus with hepatocytes interacting with various immune cells through either direct/indirect means or specific immune pathways, for example, damage‐associated molecular patterns. Importantly, CD4 was down‐regulated in all MASLD patients (Figure 6B), and we observed a negative correlation between CD4+ and FGF21 (Figure S9A). In regards to CD45+CD3+CD4+ cells, we observed repression of CD4 and CD45(= PTPRC) in patients with increased FGF21 expression (Figure 6B), whereas CD3 is up‐regulated in all MASLD patients (see Figure S10A). Furthermore, the expression of CD4 and CD45 was negatively correlated with FGF21 (Figure S9A). Moreover, the correlation between CD4+ and the inflammation score was negative (Figure S11A), and the results agree with a landmark study which reported the specific loss of CD4+ but not CD8+ T‐cells in MASH animal models.^45^ In fact, CD8α was up‐regulated in cases with increased or unchanged FGF21 expression. Conversely, CD8β was down‐regulated irrespective of FGF21 to possibly impair T‐cell receptor interactions (Figure S10A). Amazingly, in patients with increased FGF21 expression, the major histocompatibility complex class I molecule isotypes HLA‐G and HLA‐E were repressed, and their down‐regulation indicated an impaired presentation of antigens to CD8+ and NK cells (Figure S10A). HLA‐G supports immune tolerance,^46^ and HLA‐E interacts with the natural killer inhibitory receptor KLRD1/NKG2A, which was up‐regulated in all MASLD cases^47^ (Figure S10C). Moreover, markers for mature dendritic cells (DC), that is, CD83 and SIRPA were specifically up‐regulated in patients with increased FGF21 expression. Their expression is positively correlated with FGF21 (Figure S9A), and CD83 plays major roles in T‐cell activation, Treg differentiation and DC function^48^, ^49^ while the SIRPA–CD47 axis controls phagocytosis. CD47 is expressed on all cells and signals “don't eat me.” It therefore blocks phagocytosis; however, was down‐regulated in all MASLD patients^50^ (Figure S10A). Together, the data are highly suggestive for FGF21 to dampen T‐cell mediated inflammation and sensitivity to antigen presentation.

CD163 is primarily expressed on monocytes/macrophages and functions as an immunosensor and scavenger receptor. CD163 was specifically down‐regulated in patients with increased FGF21 expression (Figure 6B). Furthermore, MARCO was repressed in patients with increased FGF21 expression and together, this underscores an anti‐inflammatory action. Conversely, CD5L, FOLR2 and SLC40A1 were repressed, and the data support the notion of FGF21 to repress M2‐polarisation. The correlations between FGF21 and CD163, MARCO, VSIG4 and MRC1/CD206 were negative (Figures S9A and S10A). Importantly, ficolin 1 and complement CFB, C3AR1, C8G were up‐regulated in MASLD and function as recognition molecules in the lectin and alternative pathway to stimulate complement activation and the formation of the membrane attack complex (MAC; Figure S12B). Following interaction with its ligand C3a, the complement receptor C3AR1 becomes pro‐inflammatory. Strikingly, C3a was specifically down‐regulated in patients with increased FGF21 expression, and therefore dampens the secretion of cytokines and chemokines by macrophages. Similarly, the factors C1QC, MASP1, FCN2, COLEC10, C3, C4A and C4B were specifically down‐regulated in patients with increased FGF21 expression, whereas C4BPB was up‐regulated, and the data highlight the intricate regulation of complement factors in MASLD (Figure S12A). Additionally, the catalytic complexes C4b and C3 of the C5 convertase were specifically down‐regulated in patients with increased FGF21 expression, whereas CFB, which function in an activation loop, was up‐regulated. This suggests FGF21 to dampen osmolysis mediated by MAC. Furthermore, in all MASLD patients complement 2 was repressed (Figure S12A,B), and except of C2, COLEC10 and C4BPB, the correlation between FGF21 and the expression of complement factors was strictly negative. Together, the data are highly suggestive for FGF21 to attenuate the complement system.

Next, we considered chemokines and cytokines secreted by Kupffer cells, monocytes, hepatic stellate cell (HSC) and neutrophils (Figure 6A). In patients with increased FGF21 expression, we observed up‐regulation of CCL2, CCL3, CCL4, CCL20, CCL22 (Figure 6C). These chemokines bind to the receptors CCR2, CCR1, CCR5, CCR6 and CCR4. Furthermore, in all MASLD patients, CCR2 and CCR5 were up‐regulated (Figure S10B). Together, the data are suggestive for monocyte mobilisation from the bone marrow and recruitment of NK, Th1 and CD8+ T‐cells.

In regards to neutrophilic infiltrates, we examined CXCR1, CXCR2 and the chemokine ligands CXCL1, 2, 5 and 8. All were repressed in MASLD patients and therefore, no obvious role of FGF21 can be ascertained. Yet, the correlations between FGF21 and most chemokines were positive, whereas the correlations between FGF21 and the receptors CCR1 and CCR2 were negative (Figure S9B). Furthermore, CXCL16 stimulates NKT cell trafficking and was specifically down‐regulated in patients with increased FGF21 expression (Figure 6C). Conversely, CXCL9‐11 were up‐regulated to stimulate NK, Th1 and Th17‐cell trafficking, and FGF21 positively influenced expression of these chemokine ligands. Together, a complex picture merges whereby FGF21 attenuated but also stimulated pro‐inflammatory responses.

NF‐κB signalling is an essential pathway in inflammation and requires RELA/p65 phosphorylation by IKBKB. Both were specifically down‐regulated in patients with increased FGF21 expression (Figures 6C and S10B). Furthermore, NFKB1 is repressed and the correlation between FGF21 and NFKB1 was strictly negative (Figure S9B). Although, NFKB2, RELB and IKBKG/NEMO were increased in all MASLD cases, the correlation between FGF21 and IKBKG/NEMO was strictly negative (Figure S9B). Together, we obtained evidence for FGF21 to diminish NFKB signalling, and this agrees with findings from a murine monocyte‐macrophage cell culture study.^51^ Additionally, JUN was specifically up‐regulated in patients with increased FGF21 expression, whereas JUNB (Figure S10B) and JUND were down‐regulated (Figure 6C). Therefore, members of the JNK signalling pathway were regulated in MASLD, and the correlations between FGF21 and JUNB, JUND were positive (Figure S9B). Although pro‐inflammatory cytokines, especially CRP, IL1B and IL15 were up‐regulated in patients with increased FGF21 expression (Figure 6C), and the correlations with FGF21 were positive (Figure S9B), the expression of the corresponding receptors IL1R2, IL6R, IL6ST and TNFRSF1A were down‐regulated. Here, the correlations with FGF21 were negative (Figure S9B). Furthermore, IL17RA and the anti‐inflammatory TGFβ1 were specifically down‐regulated in patients with increased FGF21 expression, whereas IL10RB was up‐regulated (Figure 6D). Together, we observed up‐regulation of certain pro‐inflammatory cytokines but repression of the corresponding receptors, and FGF21 positively influenced expression of anti‐inflammatory cytokines, that is, IL 10, IL10RB and IL27.

Finally, in patients with increased FGF21 expression, we noticed up‐regulation of the T‐cell and NK‐cell markers CD69, CD160, KLRB1/CD161, KLRF1/NKp80 and CD58, whereas PRF1/Perforin, B3GAT1/CD57, LAG3 and ITGA1/CD49a were repressed (Figure 6E). The data are suggestive for a complex interplay with an activation of lymphocytes and NK cells, proliferation of T‐cells and a subset of memory cells committed to a distinct pathway of differentiation.^52^ Additionally, NKp80 supports development of functionally mature NK while the costimulatory receptor CD58 induced activation of T‐ and NK cell. Notwithstanding, down‐regulation of CD57 and CD49a reduced sensitivity of NK cells to cytokines^53^ and interferon‐γ‐signalling by tissue memory T‐cell.^54^

Together, we obtained evidence for FGF21 to repress activation of the complement system and to inhibit, at least in part, monocyte/macrophage mediated inflammation.

### 3.8 FGF21 and hepatic fibrosis

**FIGURE 7:**
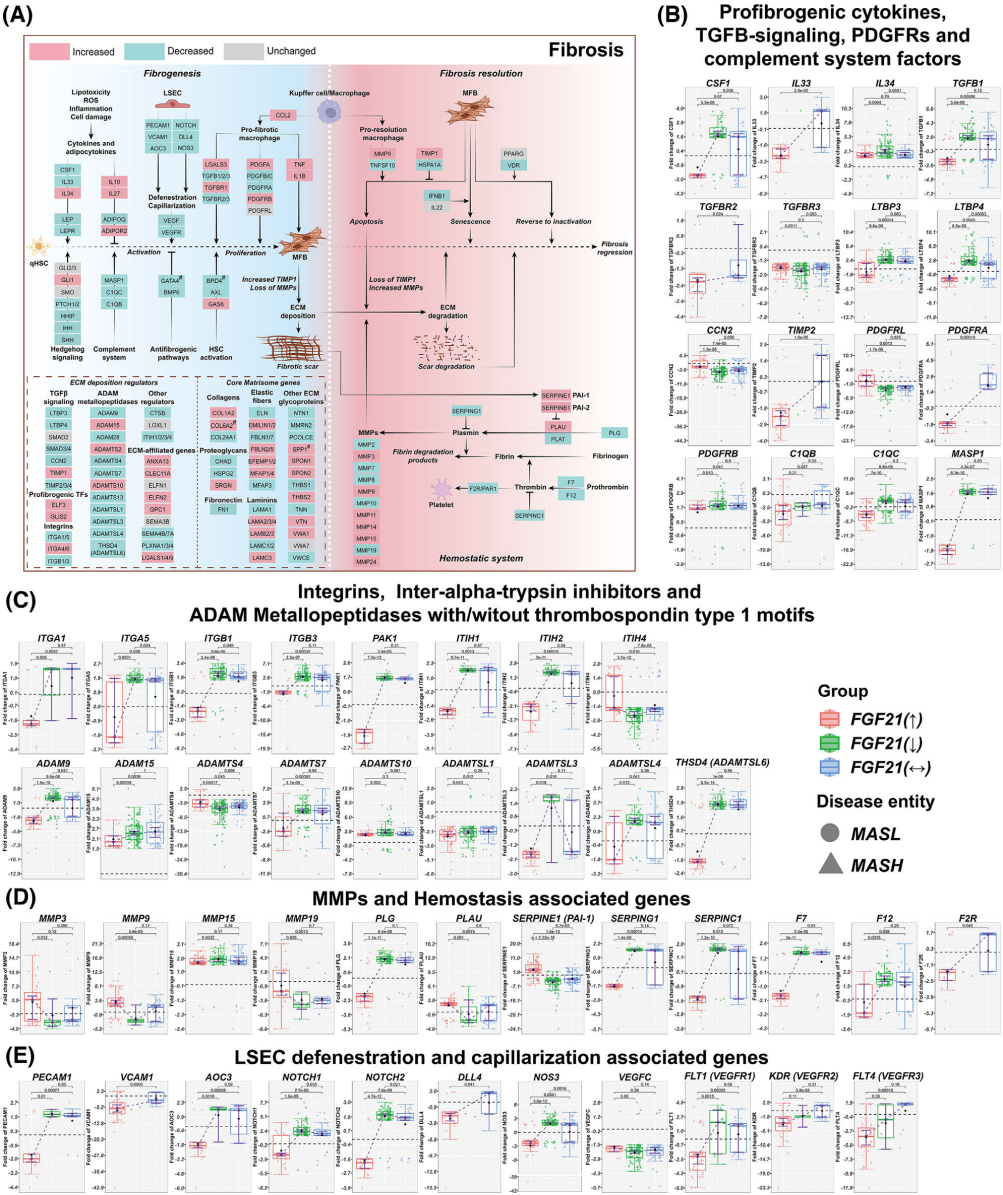
Fibrogenesis and fibrosis resolution in MASLD patients according to the hepatic FGF21 expression status. (A) DEGs coding for profibrogenic and fibrosis resolution in MASLD patients with increased FGF21 expression. Red colour represent increased, green decreased and grey unchanged DEGs expression in MASLD patients with increased FGF21. (B) DEGs coding for profibrogenic cytokines, TGFβ‐, PDGF‐signalling and complement factors. (C) DEGs coding for integrins, inter alpha trypsin inhibitors and ADAM metallopeptidases with/without thrombospondin type 1 motifs. (D) DEGs coding for MMPs and haemostasis. (E) DEGs coding for LSEC defenestration and capillarisation. The box‐plots represent changes in DEGs according to the patients FGF21‐expression status, that is, increased, decreased or unchanged. Red coloured box‐plots represent increased, green decreased and grey unchanged FGF21 expression. *p* Values were calculated in pair‐wise comparisons with the Wilcoxon rank‐sum test. ECM, extracellular matrix; MMPs, matrix metallopeptidase.

Recently, the ENLIVEN Phase 2b‐trial reported improvements in fibrosis following treatment of MASH patients with the FGF21‐analogue Pegozafermin.^55^ Here, we also report significant differences in fibrosis grades in relation to the FGF21 expression status (Table 1). The median fibrosis score is significantly lower in patients with increased FGF21 expression (Table 1, *p* = .008), and although the data are highly scattered, the correlation between FGF21 and fibrosis grades is significant (*p* = .025) and negative (Figure 1D). In fact, the proportion of *F* = 2—4 was the lowest in MASLD cases with increased FGF21 expression. To gain insight into FGF21's anti‐fibrotic effects, we considered various cellular and molecular mechanisms. Figure 7A depicts DEGs in patients with increased FGF21 expression, and we report the strength of the relationship between FGF21 and fibrogenic genes in Figure S13‐15.

Remarkably, the profibrogenic cytokines CSF1, IL‐33 and TGFβ were uniquely repressed in patients with increased FGF21 expression (Figure 7B), with major implications for macrophage polarisation and fibrosis.^56^ Furthermore, the TGFβ binding proteins LTBP3 and LTBP4 were repressed in patients with increased FGF21 expression (Figure 7B). These function as critical mediators of TGFβ stimulated ECM deposition and through binding to specific integrins stimulate latent TGFβ activity.^57^ Outstandingly, integrins up‐regulated in liver fibrosis, that is, ITGA1, ITGA5, ITGB1 and ITGB3 were specifically repressed in patients with increased FGF21 expression (Figure 7C).^58^, ^59^ Typically, these integrins stimulate profibrogenic cytokine release by immune cells. We observed strict negative correlations between FGF21 and fibrosis‐related gene regulations (Figure S13A,B). Moreover, the p21‐activated kinase was specifically repressed in patients with increased FGF21 expression, and its inhibition inactivates profibrotic myofibroblasts.^60^ The specific repression of intra‐alpha‐trypsin‐inhibitors ITIH1 and ITIH2 in patients with increased FGF21 expression leads to unstable ECM, and this supports fibrosis resolution^61^ (Figure 7C) while the negative relationship between FGF21 and profibrogenic genes (Figure S13B) underscores its therapeutic potential.

ADAM proteases are critical effectors of fibrosis^62^ and their regulation in MASLD is complex. ADAM9, ADAMTSL3, THSD4/ADAMTSL6 and ADAMTS7 were specifically down‐regulated in patients with increased FGF21 expression. Note, ADAM9 promotes liver injury and is up‐regulated in myofibroblasts,^63^ whereas ADAMTSL‐6 stimulates fibrillin‐1 matrix assembly and HSC activation.^64^ Their repression in patients with increased FGF21 expression is beneficial. However, down‐regulation of ADAMTS7 is disadvantageous, given its role in the degradation α2‐macroglobulin. In fact, we obtained a positive correlation between α2‐macroglobulin expression and fibrosis grades (Figure S15A).

Tissue repair requires matrix metalloproteases which degrade ECM, and we observed up‐regulation of several MMPs in relation to fibrosis grades. This included MMP2&3, MMP7, MMP9&10, MMP14&15 and MMP24. Except for MMP2 and MMP15, the correlations between MMPs and fibrosis grades were positive (Figure S15A). Importantly, MMP9 activation via the plasmin/MMP‐3 cascade enhances ECM degradation,^65^ and MMP3 and MMP9 were specifically up‐regulated in patients with increased FGF21 expression. Furthermore, plasminogen activator (PLAU) was specifically up‐regulated in patients with increased FGF21 expression, however, SERPING1 was down‐regulated. Next to its function as an inhibitor of complement C1, it inhibits plasmin. Together, this leads to an increase in plasmin levels to support fibrosis resolution (Figure 7D). Although, the plasmin inhibitor PAI‐1/SERPINE1 was up‐regulated in patients with increased FGF21 expression, a meta‐analysis showed PAI‐1 blood levels in MASH not to differ from healthy controls.^66^ Therefore, the results are inconclusive. Considering the relationship between FGF21 and MMPs, most correlations were positive and included the plasmin inhibitor PAI‐1. Conversely, the relationship between FGF21 and SERPING1 and the thrombin inhibitor SERPINC1 were negative (Figure S13C).

Because of the known haemostasis disorders in MASLD,^67^ we investigated the regulation of coagulation factors and markers of fenestration of liver sinusoidal endothelial cells (LSEC). Factor 7, which is activated by thrombin, F12 that participates in thrombin generation and the thrombin receptor F2R/PAR1 were specifically repressed in patients with increased FGF21 expression (Figure 7D). Therefore, FGF21 repressed procoagulants to revert procoagulant imbalance in MASLD, and given the interplay between inflammation and thrombosis, FGF21 protected from micro‐thrombosis through its anti‐inflammatory and anticoagulation properties. In fact, the correlation between FGF21 and the coagulation factors was strictly negative (Figure S13C). Moreover, ballooned hepatocytes impair proper perfusion of sinusoids which results in circulation disorders.^68^

Several adhesion molecules of LSEC were specifically down‐regulated in patients with increased FGF21 expression, for example, PECAM1/CD31 and AOC3/VAP‐1. Moreover, VCAM1 was repressed in cases with no change in FGF21 expression (Figure 7E). Since their overexpression leads to fenestration loss, their repression in patients with increased FGF21 expression is beneficial. Note, the VAP‐1 inhibitor TERN‐201 entered clinical trials for MASH. Additionally, MASLD‐related NOTCH signalling causes defenestration, and its ligand DLL4 triggers NOTCH1&4 signalling. Strikingly, NOTCH1&2, NOTCH4 and DLL4 were specifically repressed in patients with increased FGF21 expression, and preclinical research demonstrated DLL4 inhibition to be of therapeutic benefit in fibrosis^69^ (Figures 7E and S14D). Together, FGF21 protected against defenestration/capillarisation of LSEC. Although repression of eNOS/NOS3 in patients with increased FGF21 expression is a perplexing finding, it may also protect against excessive nitrosative stress.

Vascular endothelial growth factors (VEGFs) and their receptors are important regulators of LSEC fenestration. Their repression contributed to defenestration (Figures 7E and S14D). Notwithstanding, repression of VEGFC might be beneficial since its overexpression caused insulin resistance and weight gain in transgenic mice. The correlations between FGF21 and the growth factors VEGFB&C were positive but negative for the VEGF‐receptors, that is FLT1/VEGFR1, KDR/VEGFR2 and FLT4/VEGFR3 (Figure S13C). Together, the results underscore the fine‐tuning of VEGF signalling by FGF21.

In regards to ECM remodelling, we evaluated >1000 genes. The expression of COLs, laminins and ADAMs/ADAMTS increased with fibrosis grades (Figure S15A). However, the predominant negative correlations between FGF21 and ECM coding genes demonstrated the crucial anti‐fibrotic action of FGF21. For instance, MFAP3 was specifically down‐regulated in patients with increased FGF21 expression and is known to stabilise ECM and elastic fibre formation. Among the PGs, chondroadherin and HSPG2 were predominantly repressed in patients with increased FGF21 expression (Figure S15B). This supports fibrosis resolution.

Finally, FGF2 was specifically up‐regulated in patients with increased FGF21 expression, and independent research demonstrated low‐molecular‐weight FGF2 to attenuate hepatic fibrosis through epigenetic down‐regulation of NOTCH‐ligand DLL1.^70^ We obtained evidence for FGF13 and FGF14 to be up‐regulated in patients with increased FGF21 expression and observed positive correlations between FGF21 and FGF2, FGF13, FGF14 (Figure S15C). Therefore, we obtained evidence for the complex interplay of several FGFs in MASLD.

## 4 DISCUSSION

Clinical evidence supports a unique role of FGF21 as a therapeutic agent for MASLD, and in a perspective, the autocrine/paracrine and endocrine actions of FGF21 have been summarised.^71^ Furthermore, the current status of FGF21 analogues in clinical trials was the subject of two recent reviews.^3^, ^4^ Although the knowledge gain is substantial, the molecular actions of FGF21 in MASLD on a genome‐wide scale remain uncertain. To the best of our knowledge only very few studies reported hepatic FGF21 expression in MASLD clinical cases. Through comprehensive genomics of 396 liver biopsies, we identified patients with either increased (30%), decreased (40%) or unchanged (30%) hepatic FGF21 expression. Among cases with decreased FGF21, the levels might have dropped to 10% of healthy controls. Conversely, among patients with increased FGF21 expression, we observed up to 21‐fold increases. The genomic study enabled mechanistic insight as to why patients differed in the FGF21 expression which can be attributed to differences in the transcriptional control of the FGF21 gene. Given the differences in FGF21 expression, however, not all patients might equally benefit from treatment with FGF21 analogues. In fact, there is clear evidence for patients with decreased FGF21 expression to present higher disease scores. Specifically, patients with decreased FGF21 expression are characterised by more cases with higher, that is, 7–8 NAS scores (*p* = .008, Table 1). Moreover, based on median NAS scores, we observed borderline statistical significance for patients with decreased FGF21 expression (*p* = .071). In regards to lobular inflammation, patients with decreased FGF21 expression are overrepresented for cases scored 3, and similar results were obtained for hepatocyte ballooning (Table 1). Together, disease activities differed between patients with increased and decreased FGF21 expression, and recent clinical data for Efruxifermin showed that 58% of patients had either improved NAS scores or resolved from MASH. Although direct comparisons between patients with endogenously increased FGF21 expression cannot directly be compared with patients treated with FGF21 analogues, there is clear evidence for patients with increased FGF21 expression to be better equipped in alleviating the detrimental effects of MASLD. In regards to clinical trials, not all patients benefitted from FGF21 treatment, and we speculate that non‐responders might be patients with FGF21 resistance or endogenously increased FGF21 expression. Notwithstanding, the numbers of patients in Phase 2B clinical trials remain small, and similar results were obtained for Pegozafermin.

By comparing genomic data of the three cohorts with either increased, decreased or unchanged FGF21 expression, we gained deep insights into FGF21 MoA and discovered new molecular therapeutic targets. Remarkably, most FGF21 targets are consistently regulated, even though the magnitude of change differed between patients. In cases with increased FGF21 expression >88.6% of DEGs are regulated in the same direction, whereas for the remaining 11.4%, we noted opposite regulation among some patients. Similar results were obtained for patients with decreased FGF21 expression, that is, 89.6% (Figure S16). Therefore, the results are robust and consistent among patients. The fact that FGF21 transcription differed between patients is an important finding and raises the question whether stimulation of endogenous FGF21 transcription would be therapeutically advantageous for instance by targeting ATF3, ATF4 and/or CEBPG. This would be a different approach when compared with treatment of MASLD patients with synthetic FGF21 analogues. Strikingly, patients with increased FGF21 expression presented lower NAS disease activity scores, showed lower scores in lobular inflammation and fibrosis grades (Figure 1D), and therefore the data are suggestive for some patients to effectively ameliorate metabolic dysfunction through increased expression of endogenous FGF21. The clinical data are also suggestive for patients with increased “endogenous” FGF21 expression not to develop FGF21 resistance. However, in regards to hepatocyte ballooning the effects of FGF21 were less obvious.

In the following, we highlight some mechanistic aspects of FGF21 MoA. Specifically, we considered the recent study of Byun et al.^72^ The authors proposed a mechanism whereby FGF21 signalling activates hepatic autophagy and lipid degradation via the JMJD3 histone demethylase. Essentially, through demethylation of histone H3K27‐me3, the demethylase JMJD3 (alias KDM6B) up‐regulates global autophagy‐network genes. Furthermore, the investigators reported repressed hepatic expression of JMJD3, ATG7, LC3 and ULK1 in MASLD patients, and with the exception of ATG7, our data for MASLD patients agreed with their findings.

Another mechanism relates to mTORC1 which plays a pivotal role in FGF21‐mediated autophagy.^73^ mTORC1 suppresses autophagy, however, is sensitive to rapamycin which blocks its activity and stimulates autophagy.^74^, ^75^ We observed marked regulation of components of the mTORC1 complex consisting of RAPTOR/RPTOR and PRAS40/AKT1S1.^76^ RPTOR impairs mTOR kinase activity,^77^ and AKT1S1 directly inhibits mTORC1 activity by blocking the MTOR substrate‐recruitment site.^78^ Strikingly, RPTOR and AKT1S1 expression is repressed in patients with increased FGF21 expression (Figures 3C and S3B). A further example relates to SIRT1. Its deacetylation of FOXOs, ATGs and LC3 stimulates autophagy flux, whereas deacetylation of SREBF1 and p65 blocks their activity.^79^ We observed repression of SIRT1 in MASLD patients, however, show its expression to be positively regulated by FGF21 (Figure S6B). Moreover, we report expression of autophagy related genes ATG5, ATG10 and ATG 12 which were strongly induced in MASLD patients with increased FGF21 expression (Figure 3D). For its role in cargo recognition, RSP27A is of critical importance in autophagy. However, it also inhibits MDM2‐mediated p53 degradation/inactivation. Given the link between MASLD and liver cancer, we speculate that FGF21 forestalls tumour growth by stimulating autophagy and p53‐mediated apoptosis. Together, diverse data supported the conclusions whereby FGF21 stimulates autophagy.

Regarding OXPHOS there is growing evidence for FGF21 to influence mitochondrial homeostasis. Importantly, FGF21‐KO mice present severely damaged mitochondria as evidenced by transmission electron microscopy and functional assays. The number of mitochondria and the mitochondrial mass is reduced, and the mitochondria contain fewer cristae.^80^ Conversely, adeno‐viral overexpression of FGF21 in cardiomyocytes reversed the mitochondrial damage. Furthermore, siRNA‐mediated gene knock down of FGF21 in cardiomyocytes resulted in significantly reduced maximal and spare respiratory capacity of mitochondria. Together, this reinforces the notion for FGF21 to be a critical player in mitochondrial respiration. Strong evidence also stems from a recent publication whereby FGF21 modulates mitochondrial stress response (mitoISR) in cardiomyocytes under mild mitochondrial dysfunction. As stated by the authors^81^ the mitochondrial integrated stress response characterises a major adaptive pathway to respiratory chain deficiency with FGF21 functioning as a critical effector of mitoISR under mild OXPHOS dysfunction. In their study the mouse models of DARS (= mitochondrial aspartyl‐tRNA synthetase) and mitochondrial matrix protease CLPP deficiency were investigated in the presence and absence of FGF21. Unlike DARS2 deficient heart mitochondria, FGF21 regulated mitoISR upon mild mitochondrial dysfunction caused by CLPP depletion. In line with the authors’ connotation, we observed significantly reduced DARS2 expression in MASLD patients with increased FGF21 expression (Figure S5), and with few exceptions, patients with increased FGF21 expression showed marked up‐regulation of genes coding for the mitochondrial respiration change (Figure 4F).

It is well established that treatment with synthetic FGF21 analogues improved insulin sensitivity, and this effect is, in part, mediated through inhibition of mTORC1 signalling.^82^ In fact, several lines of evidence support this notion as demonstrated by Gong and colleagues^82^: First, knock‐down of β‐Klotho blocked FGF21 signalling through FGFR1 and therefore impaired FGF21's ability to improve insulin sensitivity. Second, without β‐Klotho, FGF21 was unable to inhibit mTORC1 activity. Third, adenoviral overexpression of FGF21 caused marked reduction of mTORC1 activity and the phosphorylation of insulin receptor substrate 1. Forth, mTORC1 and insulin signalling are altered in FGF21‐KO mice. Fifth, FGF21 treatment ameliorated MASH in mice given an MCD diet. Moreover, studies with the liver‐specific insulin receptor KO (LIRKO) model revealed FGF21 treatment to normalise hyperglycaemia in mice by increasing energy metabolism in brown fat. However, this was independent of insulin action in hepatocytes.^83^ Corroborative evidence stems from FGF21‐KO mice, which were characterised by lower subcutaneous adipose tissue mass and greater insulin‐resistance when given a HFD.^84^ Strikingly, transplants of subcutaneous adipose tissue from wild‐type to FGF21‐KO mice improved insulin sensitivity in the recipient.^84^ Another study showed that diet induced obesity in FGF21 liver‐specific but not FGF21 adipocyte‐specific KOs led to an increased insulin resistance but decreased brown adipose tissue‐mediated glucose metabolism.^85^ Last, in a recent review, the nuanced metabolic functions of endogenous FGF21 were summarised which depended on the nature of the stimulus, tissue source and the experimental model.^86^ Together, there is compelling evidence for FGF21 to improve insulin sensitivity in MASLD patients through different mechanisms, and we observed highly significant correlations between FGF21 and genes coding for energy sensing (Figure S4A). In fact, our study findings agree with the proposed mechanisms of FGF21 mediated glucose metabolism. Together, FGF21 exerts major effects on mitochondrial respiration with OXPHOS specifically up‐regulated in patients with increased FGF21 expression (Figure 4). Thus, FGF21 based therapies will improve ATP yield and energy balance. Furthermore, FGF21 stimulated pyruvate uptake into mitochondria but repressed its metabolism by PC and PCK2 to attenuate gluconeogenesis and lipogenesis. In patients with increased FGF21 expression, we noticed marked repression of DEGs coding for glycogen storage and its glucose release. Considering the high extracellular but low intracellular glucose levels in MASLD patients, FGF21 supports glycaemic control. Furthermore, FGF21 improved insulin sensitivity, and we observed positive correlations between FGF21 and the INSR and IRS2. Although DEGs coding for the glycolytic pathway were up‐regulated, their expression was negatively influenced by FGF21. Therefore, FGF21 dampened glucose flux in MASLD.

In regards to lipid metabolism, FGF21 repressed lipid transporters (Figure 5) and therefore protected hepatocytes from lipid overload. Strikingly, key enzymes of TAG synthesis were repressed in patients with increased FGF21 expression, and we observed an inverse relationship between FGF21 and DEGs coding for hepatic lipid synthesis. Moreover, FGF21 stimulated expression of several ACSs and therefore FAO. Although most lipogenic genes are up‐regulated in MASLD patients, the correlations between FGF21 and lipogenic genes were strictly negative. Thus, FGF21 dampened lipogenesis but induced FAO by different means. The final product of FAO is acetyl‐CoA which enters the TCA cycle. Equally, pyruvate metabolism by PDHs fuels acetyl‐CoA into the TCA cycle which results in an overflow of acetyl‐CoA and eventually leads to mitochondrial dysfunction as seen in insulin‐resistance MASLD mice.^39^ In patients with increased FGF21 expression, most DEGs coding for TCA cycle were repressed to protect mitochondria from dysfunction. Together, FGF21 stabilised mitochondria and improved energy supply in MASLD. Meanwhile, FGF21 stimulated hepatic expression of the peroxisome proliferator‐activated receptor γ coactivator protein‐1α (PGC1α/PPARGC1A). This protein functions as a transcriptional coactivator and metabolic regulator.^87^ It is a critical effector of energy homeostasis by stimulating FAO. Specifically, FGF21‐KO mice failed to respond to starvation with an increased PGC1α expression, and we observed mostly repressed PGC1α expression in MASLD patients (Figure 5E). However, acetyl‐CoA carboxylase‐β (ACC2/ACACB) which catalyses the rate‐limiting step in FA synthesis, was specifically repressed in patients with increased FGF21 expression (Figure 5E). Additionally, we observed positive correlations between FGF21 and the expression of inhibitors of SREBF1 especially SIRT1 and INSIG2 (Figure S8D). Note, SREBF1 stimulates cholesterol and lipid synthesis. Further evidence for the relevance of our findings (see Figures 5, S6 and S7) can be derived from studies with diet‐induced obese (DIO) mice.^88^ Treatment of DIO mice with synthetic FGF21 analogues led to repressed hepatic gene expression in lipogenesis, notably the ACC1 and ACC2, GPAT (glycerolphosphat‐O‐acyltransferase), DGAT1/2, SCD1 (= stearoyl‐CoA desaturase) and FASN (= FA synthase). Additionally, this treatment caused repression of genes coding for cholesterol and BA metabolism, and the results are similar between FGF21 treated DIO mice and MASLD patients with increased FGF21 expression (see Figures 5F, S6 and S7). Here the correlations between FGF21 and expression of HMGCR, CYP8B1, ABCA1 and HMGCS2 were strictly negative. Together, FGF21 supports repression of key enzymes in lipogenesis though different means.

Moreover, we assessed a large set of immune cell markers, cytokines and chemokines and obtained clear evidence for FGF21 to repress major components of the complement system and to inhibit monocyte/macrophage mediated inflammation. Although FGF21 stimulated some pro‐inflammatory cytokines, FGF21 repressed the expression of the corresponding receptors, at least in part. Independent studies reported FGF21 to improve hepatic inflammation and steatosis, and in our study (Table 1), we found patients with increased FGF21 expression to be less frequently scored 3 for lobular inflammation. This score defines >4 inflammatory foci per 200× optical field. Mechanistically, there is good evidence for FGF21 to directly interact with FGF receptors of macrophages whose expression is up‐regulated during macrophage polarisation.^89^ Importantly, M2 macrophages support anti‐inflammatory responses, and experimental evidence suggests macrophage‐specific FGFR1 deletion to alleviate high‐fat‐diet‐induced liver inflammation by inhibiting MAPKs/TNF pathways.^90^ Moreover, FGFR1 KO prevented HFD‐induced liver lipid accumulation and fibrosis^90^ while disruption of FGF signalling ameliorates inflammatory response in HSCs.^91^ It is of considerable importance that FGFR3 and FGFR4 were specially repressed in patients with increased FGF21 expression. However, FGFR1 is repressed among all MASLD patients (Figure 2D). Furthermore, FGF21 signalling requires the coreceptor βklotho, which enables interaction of FGF21 with its receptors.^92^ In the liver, βklotho forms complexes with FGFR1, FGFR3 and FGFR4,^93^, ^94^ and as mentioned above, FGFR3 and FGFR4 were specifically repressed in patients with increased FGF21 expression. Nonetheless, expression of βklotho was inconsistent and insignificantly regulated among patients; yet, there are more cases with up‐regulated expression (*N* = 19) as compared with cases with repressed expression (*N* = 6, Figure S2A). Additionally, there is evidence for FGF21 to inhibit macrophage‐mediated inflammation by activating Nrf2 and suppressing the NFκB signalling pathway.^51^ Intriguingly, RELA (= p65 of NFKB) was specifically repressed in patients with increased FGF21 expression (Figure 6C), and although Nrf2/NFE2L2 expression was repressed, it did not differ between patients with increased, decreased or unchanged FGF21 expression (Figure S2A). Regarding receptors that inhibit macrophage activation,^95^ we wish to highlight CD200R1 which was significantly up‐regulated in patients with increased FGF21 expression. CD200R1 is positively correlated with FGF21 expression (Figures S9A and S10A). Although its ligand CD200 was repressed among all patients, the correlation between FGF21 and CD200 expression is positive. A further example relates to the Fc‐gamma receptor CD32b which function as a negative regulator of macrophage activation. Expression of CD32b (= FCGR2B) is significantly higher in patients with increased FGF21 expression, and the correlation between FGF21 and CD32b expression is positive as well (Figures S9C and S10C). Together, our findings highlight FGF21 anti‐inflammatory actions and are supported by the regulation of key molecules in FGFR signalling. Therefore, we consider FGFR3 and FGFR4 as bona fide targets in the treatment of MASH.

Given the interrelationship between inflammation and fibrosis, we considered different mechanisms in the scarring of the liver. This includes regulation of profibrogenic cytokines, ADAM proteases as well as other metalloproteases as critical effectors of fibrosis. We already discussed major findings in the results section, nonetheless wish to emphasise the specific repression of TGFβ1 in patients with increased FGF21 expression. Importantly, TGFβ signalling is strongly up‐regulated in fibrosis, and FGF‐21 treatment of mice attenuated hepatic fibrosis by inhibiting the expression of TGF‐β at the mRNA and protein level. In addition, FGF21 impaired phosphorylation of Smad2/3 signalling proteins.^96^ Moreover, we evaluated the regulation of > 1000 genes coding for ECM remodelling, and although the expression of COLs, laminins and ADAMs/ADAMTS increased with fibrosis grades, we obtained clear evidence for an antifibrotic function of FGF21 (Figure 7). For instance, MFAP3 was specifically repressed in patients with increased FGF21 expression, and this protein stabilises the elastic fibre formation. We also investigated the regulation of coagulation factors and markers of fenestration of liver sinusoidal cells and obtained evidence for FGF21 to repress procoagulants and to revert procoagulant imbalance in MASLD (Figures 7 and S13). Conversely, metalloproteinases, which resolve fibrotic scares, were up‐regulated (Figure 7). Together, several PGs including HSPG2 and chondroadherin were repressed which supports scar resolution in patients with increased FGF21 expression. Finally, we investigated expression of markers of fenestration of LSEC and obtained evidence for FGF21 to protect LSEC. We also observed up‐regulation of FGF2, 13 and 14 in patients with increased FGF21 expression, and this broadens the perspective of FGFs in metabolic disorders.

### 4.1 Study limitations

The following caveats need to be considered. First, MASLD is a heterogeneous population and a dynamic disease. Therefore, a single liver biopsy may not capture dynamic changes. Second, we considered patients with endogenously increased FGF21 expression and speculated that patients treated with FGF21 synthetic analogues might lead to similar changes in genomic responses. However, whether synthetic FGF21 analogues will elicit similar effects as endogenous FGF21 remains unknown, and such treatment may elicit extrahepatic effects which might lead to additional benefits. Third, it would have been desirable to confirm results based on liver biopsies from patients participating in FGF21 clinical trials. Fourth, validation of FGF21 targets by immunohistochemistry is worthwhile. However, liver biopsies carry the risk of bleeding, and the competing interests for a tiny amount of tissue are significant constrains. Fifth, molecular stratification of patients can only be one aspect in clinical trials and verification.

## 5 CONCLUSION

We report significant heterogeneity in the endogenous expression of FGF21 in MASLD and discovered a large set of validated target genes regulated by FGF21. We propose novel targets for its potential therapeutic use.

## AUTHOR CONTRIBUTIONS

*Data acquisition, analysis and interpretation; preparation of artworks; contributed to the design of the study and initial writing*: Shifang Tang. *Conception and design of the study; data analysis and interpretation; writing of the final manuscript*: Jürgen Borlak. Both authors approved the final version of the manuscript.

## CONFLICT OF INTEREST STATEMENT

The authors declare no conflicts to interest.

## ETHICS STATEMENT

Research was carried out according to the Declaration of Helsinki as revised in 2013 and ethical approval for the use of liver samples was obtained from the Ethics Committee of the Hannover Medical School (Tr/L, 2499 and Tr/L, 466 31309). A total of 22 biopsies were obtained, immediately frozen and stored at −80°C. In addition, healthy liver specimens from N = 6 patients served as controls. See also Sahini and Borlak. Translational Research, Volume 177, 2016 [PMID: 38358517].^97^

## Supporting information



Supporting Information

Supporting Information

## References

[1] Younossi ZM , Golabi P , Paik JM , Henry A , Van Dongen C , Henry L . The global epidemiology of nonalcoholic fatty liver disease (NAFLD) and nonalcoholic steatohepatitis (NASH): a systematic review. Hepatology. 2023;77(4):1335‐1347. 36626630 10.1097/HEP.0000000000000004 PMC10026948

[2] Tang S , Borlak J . Genomics of human NAFLD: lack of data reproducibility and high interpatient variability in drug target expression as major causes of drug failures. Hepatology. 2024;80(4):901‐915. 38358517 10.1097/HEP.0000000000000780 PMC11407777

[3] Chui ZSW , Shen Q , Xu A . Current status and future perspectives of FGF21 analogues in clinical trials. Trends Endocrinol Metab. 2024;35(5):371‐384. 38423900 10.1016/j.tem.2024.02.001

[4] Harrison SA , Rolph T , Knott M , Dubourg J . FGF21 agonists: an emerging therapeutic for metabolic dysfunction‐associated steatohepatitis and beyond. J Hepatol. 2024;81(3):562‐576. 38710230 10.1016/j.jhep.2024.04.034

[5] Breher‐Esch S , Sahini N , Trincone A , Wallstab C , Borlak J . Genomics of lipid‐laden human hepatocyte cultures enables drug target screening for the treatment of non‐alcoholic fatty liver disease. BMC Med Genomics. 2018;11(1):111‐117. 30547786 10.1186/s12920-018-0438-7 PMC6295111

[6] Li H , Fang Q , Gao F , et al. Fibroblast growth factor 21 levels are increased in nonalcoholic fatty liver disease patients and are correlated with hepatic triglyceride. J Hepatol. 2010;53(5):934‐940. 20675007 10.1016/j.jhep.2010.05.018

[7] Kleiner DE , Brunt EM , Van Natta M , et al. Design and validation of a histological scoring system for nonalcoholic fatty liver disease. Hepatology. 2005;41(6):1313‐1321. 15915461 10.1002/hep.20701

[8] Hughes CE , Nibbs RJB . A guide to chemokines and their receptors. FEBS J. 2018;285(16):2944‐2971. 29637711 10.1111/febs.14466 PMC6120486

[9] Cameron MJKD . Cytokines, chemokines and their receptors. Madame Curie Bioscience Database [Internet]. Landes Bioscience; 2013.

[10] Aizarani N , Saviano A , et al. A human liver cell atlas reveals heterogeneity and epithelial progenitors. Nature. 2019;572(7768):199‐204. 31292543 10.1038/s41586-019-1373-2 PMC6687507

[11] Newman AM , Liu CL , Green MR , et al. Robust enumeration of cell subsets from tissue expression profiles. Nat Methods. 2015;12(5):453‐457. 25822800 10.1038/nmeth.3337 PMC4739640

[12] Arteel GE , Naba A . The liver matrisome—looking beyond collagens. JHEP Rep. 2020;2(4):100115. 32637906 10.1016/j.jhepr.2020.100115 PMC7330160

[13] Naba A , Clauser KR , Whittaker CA , Carr SA , Tanabe KK , Hynes RO . Extracellular matrix signatures of human primary metastatic colon cancers and their metastases to liver. BMC Cancer. 2014;14:518‐518. 25037231 10.1186/1471-2407-14-518 PMC4223627

[14] Conover WJ . Practical Nonparametric Statistics. 2nd ed. New York: Wiley, 1980.

[15] Kelley K . Confidence intervals for standardized effect sizes: theory, application, and implementation. J Stat Soft. 2007;20(8):1.

[16] Wan X , Lu X , Xiao Y , et al. ATF4‐ and CHOP‐dependent induction of FGF21 through endoplasmic reticulum stress. Biomed Res Int. 2014;2014:807874. 24900988 10.1155/2014/807874 PMC4037570

[17] Erickson A , Moreau R . The regulation of FGF21 gene expression by metabolic factors and nutrients. Horm Mol Biol Clin Investig. 2016;30(1). /j/hmbci.2017.30.issue–0016. 10.1515/hmbci-2016-0016 27285327

[18] Chen M , Liu Y , Yang Y , et al. Emerging roles of activating transcription factor (ATF) family members in tumourigenesis and immunity: implications in cancer immunotherapy. Genes Dis. 2021;9(4):981‐999. 35685455 10.1016/j.gendis.2021.04.008 PMC9170601

[19] Neill G , Masson GR . A stay of execution: ATF4 regulation and potential outcomes for the integrated stress response. Front Mol Neurosci. 2023;16:1112253. 36825279 10.3389/fnmol.2023.1112253 PMC9941348

[20] Zhang Y , Fang B , Damle M , et al. HNF6 and rev‐erbα integrate hepatic lipid metabolism by overlapping and distinct transcriptional mechanisms. Genes Dev. 2016;30(14):1636‐1644. 27445394 10.1101/gad.281972.116 PMC4973293

[21] Kumar R , Mal K , Razaq MK , et al. Association of leptin with obesity and insulin resistance. Cureus. 2020;12(12):e12178. 33489589 10.7759/cureus.12178 PMC7815269

[22] Dagogo‐Jack S . Leptin and insulin sensitivity: endogenous signals of metabolic homeostasis. J Clin Endocrinol Metab. 2024;109(5):e1402‐e1403. 37943695 10.1210/clinem/dgad653

[23] Emanuelli B , Vienberg SG , Smyth G , et al. Interplay between FGF21 and insulin action in the liver regulates metabolism. J Clin Invest. 2014;124(2):515‐527. 24401271 10.1172/JCI67353 PMC3904602

[24] Peng H , Chiu T , Liang Y , et al. PRAP1 is a novel lipid‐binding protein that promotes lipid absorption by facilitating MTTP‐mediated lipid transport. J Biol Chem. 2021;296:100052. 33168624 10.1074/jbc.RA120.015002 PMC7949078

[25] Park J , Lee D , Kim D . Redefining the role of AMPK in autophagy and the energy stress response. Nat Commun. 2023;14(1):2994. 37225695 10.1038/s41467-023-38401-z PMC10209092

[26] Tsay A , Wang J . The role of PIK3R1 in metabolic function and insulin sensitivity. Int J Mol Sci. 2023;24(16):12665. doi:10.3390/ijms241612665 37628845 PMC10454413

[27] Wang RC , Wei Y , An Z , et al. Akt‐mediated regulation of autophagy and tumorigenesis through beclin 1 phosphorylation. Science. 2012;338(6109):956‐959. 23112296 10.1126/science.1225967 PMC3507442

[28] Inoki K , Li Y , Zhu T , Wu J , Guan K . TSC2 is phosphorylated and inhibited by akt and suppresses mTOR signalling. Nat Cell Biol. 2002;4(9):648‐657. 12172553 10.1038/ncb839

[29] Kim YC , Guan K . mTOR: a pharmacologic target for autophagy regulation. J Clin Invest. 2015;125(1):25‐32. 25654547 10.1172/JCI73939 PMC4382265

[30] Luo J , Zhao H , Chen L , Liu M . Multifaceted functions of RPS27a: an unconventional ribosomal protein. J Cell Physiol. 2023;238(3):485‐497. 36580426 10.1002/jcp.30941

[31] Man SM , Kanneganti T . Regulation of lysosomal dynamics and autophagy by CTSB/cathepsin B. Autophagy. 2016;12(12):2504‐2505. 27786577 10.1080/15548627.2016.1239679 PMC5173259

[32] Liang Q , Zhong L , Zhang J , et al. FGF21 maintains glucose homeostasis by mediating the cross talk between liver and brain during prolonged fasting. Diabetes. 2014;63(12):4064‐4075. 25024372 10.2337/db14-0541

[33] Ozcan L , Wong CCL , Li G , et al. Calcium signaling through CaMKII regulates hepatic glucose production in fasting and obesity. Cell Metab. 2012;15(5):739‐751. 22503562 10.1016/j.cmet.2012.03.002 PMC3348356

[34] Stark R , Pasquel F , Turcu A , et al. Phosphoenolpyruvate cycling via mitochondrial phosphoenolpyruvate carboxykinase links anaplerosis and mitochondrial GTP with insulin secretion. J Biol Chem. 2009;284(39):26578‐26590. 19635791 10.1074/jbc.M109.011775 PMC2785346

[35] Ye Q , Liu Y , Zhang G , et al. Deficiency of gluconeogenic enzyme PCK1 promotes metabolic‐associated fatty liver disease through PI3K/AKT/PDGF axis activation in male mice. Nat Commun. 2023;14(1):1402‐1403. 36918564 10.1038/s41467-023-37142-3 PMC10015095

[36] Mutel E , Abdul‐Wahed A , Ramamonjisoa N , et al. Targeted deletion of liver glucose‐6 phosphatase mimics glycogen storage disease type 1a including development of multiple adenomas. J Hepatol. 2011;54(3):529‐537. 21109326 10.1016/j.jhep.2010.08.014

[37] Choi SY , Hirata K , Ishida T , Quertermous T , Cooper AD . Endothelial lipase: a new lipase on the block. J Lipid Res. 2002;43(11):1763‐1769. 12401876 10.1194/jlr.r200011-jlr200

[38] Cao Y , Pearman AT , Zimmerman GA , McIntyre TM , Prescott SM . Intracellular unesterified arachidonic acid signals apoptosis. Proc Natl Acad Sci USA. 2000;97(21):11280‐11285. 11005842 10.1073/pnas.200367597 PMC17191

[39] Satapati S , Sunny NE , Kucejova B , et al. Elevated TCA cycle function in the pathology of diet‐induced hepatic insulin resistance and fatty liver. J Lipid Res. 2012;53(6):1080‐1092. 22493093 10.1194/jlr.M023382 PMC3351815

[40] Yang C , Ko B , Hensley CT , et al. Glutamine oxidation maintains the TCA cycle and cell survival during impaired mitochondrial pyruvate transport. Mol Cell. 2014;56(3):414‐424. 25458842 10.1016/j.molcel.2014.09.025 PMC4268166

[41] Zhu L , Luu T , Emfinger CH , et al. CETP inhibition improves HDL function but leads to fatty liver and insulin resistance in CETP‐expressing transgenic mice on a high‐fat diet. Diabetes. 2018;67(12):2494‐2506. 30213825 10.2337/db18-0474 PMC6245220

[42] Chiang JYL . Regulation of bile acid synthesis: pathways, nuclear receptors, and mechanisms. J Hepatol. 2004;40(3):539‐551. 15123373 10.1016/j.jhep.2003.11.006

[43] Nie B , Park HM , Kazantzis M , et al. Specific bile acids inhibit hepatic fatty acid uptake in mice. Hepatology. 2012;56(4):1300‐1310. 22531947 10.1002/hep.25797 PMC3445775

[44] Nimer N , Choucair I , Wang Z , et al. Bile acids profile, histopathological indices and genetic variants for non‐alcoholic fatty liver disease progression. Metabolism. 2021;116:154457. 33275980 10.1016/j.metabol.2020.154457 PMC7856026

[45] Ma C , Kesarwala AH , Eggert T , et al. NAFLD causes selective CD4(+) T lymphocyte loss and promotes hepatocarcinogenesis. Nature. 2016;531(7593):253‐257. 26934227 10.1038/nature16969 PMC4786464

[46] Amiot L , Vu N , Samson M . Biology of the immunomodulatory molecule HLA‐G in human liver diseases. J Hepatol. 2015;62(6):1430‐1437. 25772038 10.1016/j.jhep.2015.03.007

[47] Lee N , Llano M , Carretero M , et al. HLA‐E is a major ligand for the natural killer inhibitory receptor CD94/NKG2A. Proc Natl Acad Sci USA. 1998;95(9):5199‐5204. 9560253 10.1073/pnas.95.9.5199 PMC20238

[48] Li Z , Ju X , Silveira PA , et al. CD83: activation marker for antigen presenting cells and its therapeutic potential. Front Immunol. 2019;10:1312. 31231400 10.3389/fimmu.2019.01312 PMC6568190

[49] Grosche L , Knippertz I , König C , et al. The CD83 molecule—an important immune checkpoint. Front Immunol. 2020;11:721. 32362900 10.3389/fimmu.2020.00721 PMC7181454

[50] Barclay AN , Van den Berg TK . The interaction between signal regulatory protein alpha (SIRPα) and CD47: structure, function, and therapeutic target. Annu Rev Immunol. 2014;32:25‐50. 24215318 10.1146/annurev-immunol-032713-120142

[51] Yu Y , He J , Li S , et al. Fibroblast growth factor 21 (FGF21) inhibits macrophage‐mediated inflammation by activating Nrf2 and suppressing the NF‐κB signaling pathway. Int Immunopharmacol. 2016;38:144‐152. 27276443 10.1016/j.intimp.2016.05.026

[52] Fergusson JR , Fleming VM , Klenerman P . CD161‐expressing human T cells. Front Immunol. 2011;2:36. 22566826 10.3389/fimmu.2011.00036 PMC3342360

[53] Nielsen CM , White MJ , Goodier MR , Riley EM . Functional significance of CD57 expression on human NK cells and relevance to disease. Front Immunol. 2013;4:422. 24367364 10.3389/fimmu.2013.00422 PMC3856678

[54] Cheuk S , Schlums H , Gallais Sérézal I , et al. CD49a expression defines tissue‐resident CD8(+) T cells poised for cytotoxic function in human skin. Immunity. 2017;46(2):287‐300. 28214226 10.1016/j.immuni.2017.01.009 PMC5337619

[55] Loomba R , Sanyal AJ , Kowdley KV , et al. Randomized, controlled trial of the FGF21 analogue pegozafermin in NASH. N Engl J Med. 2023;389(11):998‐1008. 37356033 10.1056/NEJMoa2304286 PMC10718287

[56] Meng X , Nikolic‐Paterson DJ , Lan HY . TGF‐β: the master regulator of fibrosis. Nat Rev Nephrol. 2016;12(6):325‐338. 27108839 10.1038/nrneph.2016.48

[57] Rifkin DB , Rifkin WJ , Zilberberg L . LTBPs in biology and medicine: lTBP diseases. Matrix Biol. 2018;71–72:90‐99. 10.1016/j.matbio.2017.11.014 PMC5988920 29217273

[58] Nejjari M , Couvelard A , Mosnier JF , et al. Integrin up‐regulation in chronic liver disease: relationship with inflammation and fibrosis in chronic hepatitis C. J Pathol. 2001;195(4):473‐481. 11745680 10.1002/path.964

[59] Rahman SR , Roper JA , Grove JI , Aithal GP , Pun KT , Bennett AJ . Integrins as a drug target in liver fibrosis. Liver Int. 2022;42(3):507‐521. 35048542 10.1111/liv.15157

[60] Martin K , Pritchett J , Llewellyn J , et al. PAK proteins and YAP‐1 signalling downstream of integrin beta‐1 in myofibroblasts promote liver fibrosis. Nat Commun. 2016;7:12502. 27535340 10.1038/ncomms12502 PMC4992158

[61] Bost F , Diarra‐Mehrpour M , Martin JP . Inter‐alpha‐trypsin inhibitor proteoglycan family–a group of proteins binding and stabilizing the extracellular matrix. Eur J Biochem. 1998;252(3):339‐346. 9546647 10.1046/j.1432-1327.1998.2520339.x

[62] Schmidt‐Arras D , Rose‐John S . Regulation of fibrotic processes in the liver by ADAM proteases. Cells. 2019;8(10):1226. doi:10.3390/cells8101226 31601007 PMC6830092

[63] Schwettmann L , Wehmeier M , Jokovic D , et al. Hepatic expression of A disintegrin and metalloproteinase (ADAM) and ADAMs with thrombospondin motives (ADAM‐TS) enzymes in patients with chronic liver diseases. J Hepatol. 2008;49(2):243‐250. 18490073 10.1016/j.jhep.2008.03.020

[64] Tsutsui K , Manabe R , Yamada T , et al. ADAMTSL‐6 is a novel extracellular matrix protein that binds to fibrillin‐1 and promotes fibrillin‐1 fibril formation. J Biol Chem. 2010;285(7):4870‐4882. 19940141 10.1074/jbc.M109.076919 PMC2836092

[65] Ramos‐DeSimone N , Hahn‐Dantona E , Sipley J , Nagase H , French DL , Quigley JP . Activation of matrix metalloproteinase‐9 (MMP‐9) via a converging plasmin/stromelysin‐1 cascade enhances tumor cell invasion. J Biol Chem. 1999;274(19):13066‐13076. 10224058 10.1074/jbc.274.19.13066

[66] Alsharoh H , Ismaiel A , Leucuta D , Popa S , Dumitrascu DL . Plasminogen activator inhibitor‐1 levels in non‐alcoholic fatty liver disease: a systematic review and meta‐analysis. J Gastrointestin Liver Dis. 2022;31(2):206‐214. 35574617 10.15403/jgld-4091

[67] Tripodi A , Fracanzani AL , Primignani M , et al. Procoagulant imbalance in patients with non‐alcoholic fatty liver disease. J Hepatol. 2014;61(1):148‐154. 24657400 10.1016/j.jhep.2014.03.013

[68] Farrell GC , Teoh NC , McCuskey RS . Hepatic microcirculation in fatty liver disease. Anat Rec (Hoboken). 2008;291(6):684‐692. 18484615 10.1002/ar.20715

[69] Chen L , Gu T , Li B , et al. Delta‐like ligand 4/DLL4 regulates the capillarization of liver sinusoidal endothelial cell and liver fibrogenesis. Biochim Biophys Acta Mol Cell Res. 2019;1866(10):1663‐1675. 31233801 10.1016/j.bbamcr.2019.06.011

[70] Pan R , Xiang L , Wang P , et al. Low‐molecular‐weight fibroblast growth factor 2 attenuates hepatic fibrosis by epigenetic down‐regulation of delta‐like1. Hepatology. 2015;61(5):1708‐1720. 25501710 10.1002/hep.27649

[71] Kliewer SA , Mangelsdorf DJ . A dozen years of discovery: insights into the physiology and pharmacology of FGF21. Cell Metab. 2019;29(2):246‐253. 30726758 10.1016/j.cmet.2019.01.004 PMC6368396

[72] Byun S , Seok S , Kim Y , et al. Fasting‐induced FGF21 signaling activates hepatic autophagy and lipid degradation via JMJD3 histone demethylase. Nat Commun. 2020;11(1). 10.1038/s41467-020-14384-z PMC7010817 32042044

[73] Shen W , Yang M , Chen H , et al. FGF21‐mediated autophagy: remodeling the homeostasis in response to stress in liver diseases. Genes Dis. 2023;11(3):101027. 38292187 10.1016/j.gendis.2023.05.019 PMC10825283

[74] Laplante M , Sabatini DM . Regulation of mTORC1 and its impact on gene expression at a glance. J Cell Sci. 2013;126(8):1713‐1719. Pt. 23641065 10.1242/jcs.125773 PMC3678406

[75] Shimobayashi M , Hall MN . Making new contacts: the mTOR network in metabolism and signalling crosstalk. Nat Rev Mol Cell Biol. 2014;15(3):155‐162. 24556838 10.1038/nrm3757

[76] Wang L , Harris TE , Roth RA , Lawrence JCJ . PRAS40 regulates mTORC1 kinase activity by functioning as a direct inhibitor of substrate binding. J Biol Chem. 2007;282(27):20036‐20044. 17510057 10.1074/jbc.M702376200

[77] Kim D , Sarbassov DD , Ali SM , et al. mTOR interacts with raptor to form a nutrient‐sensitive complex that signals to the cell growth machinery. Cell. 2002;110(2):163‐175. 12150925 10.1016/s0092-8674(02)00808-5

[78] Yang H , Jiang X , Li B , et al. Mechanisms of mTORC1 activation by RHEB and inhibition by PRAS40. Nature. 2017;552(7685):368‐373. 29236692 10.1038/nature25023 PMC5750076

[79] Tian C , Huang R , Xiang M . SIRT1: harnessing multiple pathways to hinder NAFLD. Pharmacol Res. 2024;203:107155. 38527697 10.1016/j.phrs.2024.107155

[80] Yan B , Mei Z , Tang Y , et al. FGF21‐FGFR1 controls mitochondrial homeostasis in cardiomyocytes by modulating the degradation of OPA1. Cell Death Dis. 2023;14(5):311‐319. 37156793 10.1038/s41419-023-05842-9 PMC10167257

[81] Croon M , Szczepanowska K , Popovic M , et al. FGF21 modulates mitochondrial stress response in cardiomyocytes only under mild mitochondrial dysfunction. Sci Adv. 2022;8(14):eabn7105. 35385313 10.1126/sciadv.abn7105 PMC8986112

[82] Gong Q , Hu Z , Zhang F , et al. Fibroblast growth factor 21 improves hepatic insulin sensitivity by inhibiting mammalian target of rapamycin complex 1 in mice. Hepatology. 2016;64(2):425‐438. 26926384 10.1002/hep.28523 PMC5726522

[83] Emanuelli B , Vienberg SG , Smyth G , et al. Interplay between FGF21 and insulin action in the liver regulates metabolism. J Clin Invest. 2015;125(1):458. 25654556 10.1172/JCI80223 PMC4382261

[84] Li H , Wu G , Fang Q , et al. Fibroblast growth factor 21 increases insulin sensitivity through specific expansion of subcutaneous fat. Nat Commun. 2018;9(1):272‐279. 29348470 10.1038/s41467-017-02677-9 PMC5773530

[85] Markan KR , Naber MC , Ameka MK , et al. Circulating FGF21 is liver derived and enhances glucose uptake during refeeding and overfeeding. Diabetes. 2014;63(12):4057‐4063. 25008183 10.2337/db14-0595 PMC4238010

[86] Spann RA , Morrison CD , den Hartigh LJ . The nuanced metabolic functions of endogenous FGF21 depend on the nature of the stimulus, tissue source, and experimental model. Front Endocrinol (Lausanne). 2022;12:802541. 35046901 10.3389/fendo.2021.802541 PMC8761941

[87] Potthoff MJ , Inagaki T , Satapati S , et al. FGF21 induces PGC‐1alpha and regulates carbohydrate and fatty acid metabolism during the adaptive starvation response. Proc Natl Acad Sci USA. 2009;106(26):10853‐10858. 19541642 10.1073/pnas.0904187106 PMC2705613

[88] Coskun T , Bina HA , Schneider MA , et al. Fibroblast growth factor 21 corrects obesity in mice. Endocrinology. 2008;149(12):6018‐6027. 18687777 10.1210/en.2008-0816

[89] Shen L , Li Y , Zhao H . Fibroblast growth factor signaling in macrophage polarization: impact on health and diseases. Front Immunol. 2024;15:1390453. 38962005 10.3389/fimmu.2024.1390453 PMC11219802

[90] Zhao Y , Liu Z , Yan T , et al. Macrophage‐specific FGFR1 deletion alleviates high‐fat‐diet‐induced liver inflammation by inhibiting the MAPKs/TNF pathways. Acta Pharmacol Sin. 2024;45(5):988‐1001. 38279043 10.1038/s41401-024-01226-7 PMC11053141

[91] Wang C , Li Y , Li H , et al. Disruption of FGF signaling ameliorates inflammatory response in hepatic stellate cells. Front Cell Dev Biol. 2020;8:601. 32793588 10.3389/fcell.2020.00601 PMC7387415

[92] Xie Y , Su N , Yang J , et al. FGF/FGFR signaling in health and disease. Signal Transduct Target Ther. 2020;5(1):181. 32879300 10.1038/s41392-020-00222-7 PMC7468161

[93] Zhao X , Han D , Zhao C , et al. New insights into the role of klotho in inflammation and fibrosis: molecular and cellular mechanisms. Front Immunol. 2024;15:1454142. 39308872 10.3389/fimmu.2024.1454142 PMC11412887

[94] Suzuki M , Uehara Y , Motomura‐Matsuzaka K , et al. betaKlotho is required for fibroblast growth factor (FGF) 21 signaling through FGF receptor (FGFR) 1c and FGFR3c. Mol Endocrinol. 2008;22(4):1006‐1014. 18187602 10.1210/me.2007-0313 PMC5419549

[95] Ocaña‐Guzman R , Vázquez‐Bolaños L , Sada‐Ovalle I . Receptors that inhibit macrophage activation: mechanisms and signals of regulation and tolerance. J Immunol Res. 2018;2018:8695157. 29607331 10.1155/2018/8695157 PMC5828319

[96] Xu P , Zhang Y , Liu Y , et al. Fibroblast growth factor 21 attenuates hepatic fibrogenesis through TGF‐β/smad2/3 and NF‐κB signaling pathways. Toxicol Appl Pharmacol. 2016;290:43‐53. 26592322 10.1016/j.taap.2015.11.012

[97] Sahini N, , Borlak J . Genomics of human fatty liver disease reveal mechanistically linked lipid droplet-associated gene regulations in bland steatosis and nonalcoholic steatohepatitis. Transl Res. 2016;177:41‐69. 27376874 10.1016/j.trsl.2016.06.003

