# Supplementary material for: A comparative genomic study across 396 liver biopsies provides deep insight into FGF21 mode of action as a therapeutic agent in metabolic dysfunction‐associated steatotic liver disease
Source: Clin Transl Med. 2025 Feb 17;15(2):e70218. doi: 10.1002/ctm2.70218 (PMC11832436; doi:10.1002/ctm2.70218)
Supplement: Supplementary file 2 — Supporting Information [file CTM2-15-e70218-s002.pdf]

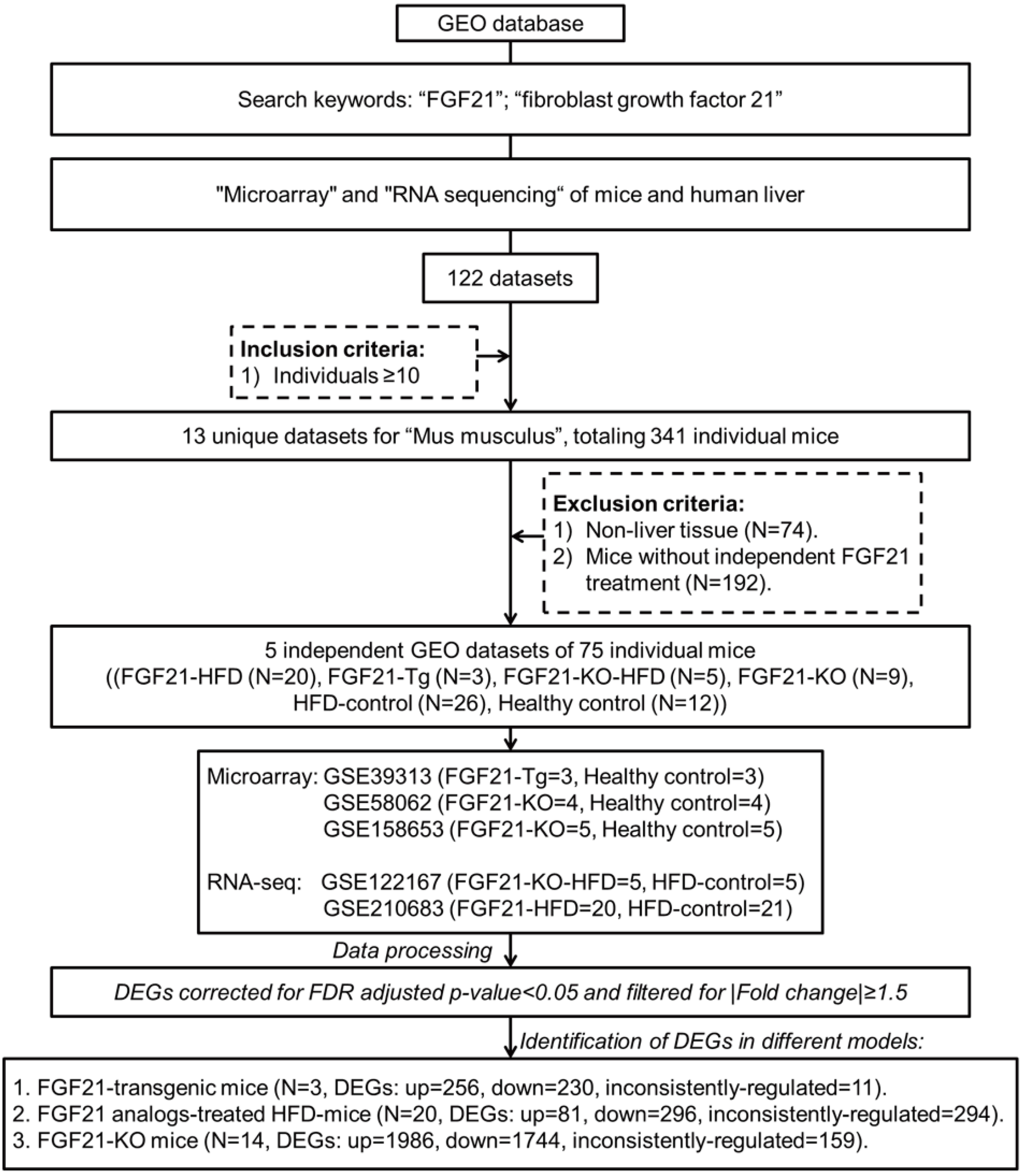
GEO database
Search keywords: “FGF21”; “fibroblast growth factor 21”
"Microarray" and "RNA sequencing“ of mice and human liver
122 datasets
Inclusion criteria:
1) Individuals ≥10
13 unique datasets for “Mus musculus”, totaling 341 individual mice
Exclusion criteria:
1) Non-liver tissue (N=74).
2) Mice without independent FGF21 treatment (N=192).
5 independent GEO datasets of 75 individual mice
((FGF21-HFD (N=20), FGF21-Tg (N=3), FGF21-KO-HFD (N=5), FGF21-KO (N=9), HFD-control (N=26), Healthy control (N=12))
Microarray: GSE39313 (FGF21-Tg=3, Healthy control=3)
GSE58062 (FGF21-KO=4, Healthy control=4)
GSE158653 (FGF21-KO=5, Healthy control=5)
RNA-seq: GSE122167 (FGF21-KO-HFD=5, HFD-control=5)
GSE210683 (FGF21-HFD=20, HFD-control=21)
Data processing
DEGs corrected for FDR adjusted p-value<0.05 and filtered for |Fold change|≥1.5
Identification of DEGs in different models:
1. FGF21-transgenic mice (N=3, DEGs: up=256, down=230, inconsistently-regulated=11).
2. FGF21 analogs-treated HFD-mice (N=20, DEGs: up=81, down=296, inconsistently-regulated=294).
3. FGF21-KO mice (N=14, DEGs: up=1986, down=1744, inconsistently-regulated=159).

Figure S1 Flowchart of independent FGF21-transgenic, -knock out or -treatment models selection.

Abbreviations: GEO, gene expression omnibus; Tg, transgenic; KO, knock out; HFD, high fat diet.

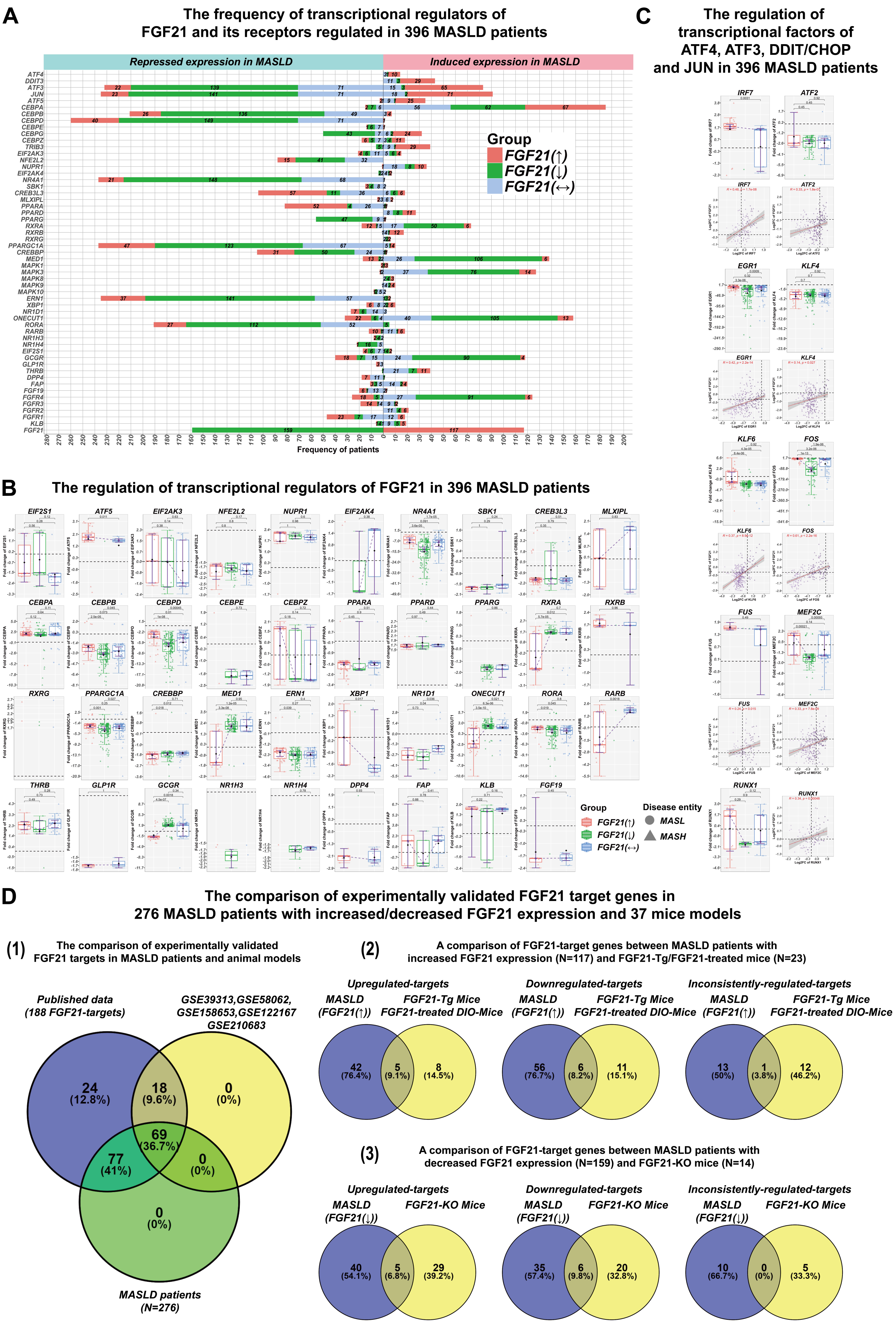
A
The frequency of transcriptional regulators of
FGF21 and its receptors regulated in 396 MASLD patients
Repressed expression in MASLD
Induced expression in MASLD
Group
FGF21(↑)
FGF21(↓)
FGF21(↔)
Frequency of patients
B
The regulation of transcriptional regulators of FGF21 in 396 MASLD patients
Group
FGF21(↑)
FGF21(↓)
FGF21(↔)
Disease entity
MASL
MASH
C
The regulation of
transcriptional factors of
ATF4, ATF3, DDIT/CHOP
and JUN in 396 MASLD patients
D
The comparison of experimentally validated FGF21 target genes in
276 MASLD patients with increased/decreased FGF21 expression and 37 mice models
(1)
The comparison of experimentally validated
FGF21 targets in MASLD patients and animal models
Published data
(188 FGF21-targets)
GSE39313,GSE58062,
GSE158653,GSE122167
GSE210683
24
(12.8%)
18
(9.6%)
0
(0%)
69
(36.7%)
77
(41%)
0
(0%)
0
(0%)
MASLD patients
(N=276)
(2)
A comparison of FGF21-target genes between MASLD patients with
increased FGF21 expression (N=117) and FGF21-Tg/FGF21-treated mice (N=23)
Upregulated-targets
MASLD
(FGF21(↑))
FGF21-Tg Mice
FGF21-treated DIO-Mice
42
(76.4%)
5
(9.1%)
8
(14.5%)
Downregulated-targets
MASLD
(FGF21(↑))
FGF21-Tg Mice
FGF21-treated DIO-Mice
56
(76.7%)
6
(8.2%)
11
(15.1%)
Inconsistently-regulated-targets
MASLD
(FGF21(↑))
FGF21-Tg Mice
FGF21-treated DIO-Mice
13
(50%)
1
(3.8%)
12
(46.2%)
(3)
A comparison of FGF21-target genes between MASLD patients with
decreased FGF21 expression (N=159) and FGF21-KO mice (N=14)
Upregulated-targets
MASLD
(FGF21(↓))
FGF21-KO Mice
40
(54.1%)
5
(6.8%)
29
(39.2%)
Downregulated-targets
MASLD
(FGF21(↓))
FGF21-KO Mice
35
(57.4%)
6
(9.8%)
20
(32.8%)
Inconsistently-regulated-targets
MASLD
(FGF21(↓))
FGF21-KO Mice
10
(66.7%)
0
(0%)
5
(33.3%)

Figure S2 Transcriptional control of FGF21 and FGF21-targets in MASLD patients.

(A) The bar charts visualize the frequency of MASLD patients with either increased, decreased or unchanged transcriptional regulators of FGF21. (B) DEGs coding for TFs of FGF21. (C) DEGs coding for upstream transcriptional regulators of TFs. The box-plots represent changes in DEGs according to the patients FGF21-expression status, i.e. increased, decreased or unchanged. Red colored box plots represent increased, green decreased and grey unchanged FGF21 expression. p-values were calculated in pair-wise comparisons with the Wilcoxon rank-sum test. Spearman correlations were computed in R and only correlation results with *p-value* <0.05 were shown. (D) The regulation of the FGF21 target genes in 276 MASLD patients and 37 mice sets with FGF21 expression significantly changed. Venn diagrams show the comparisons between the regulated FGF21 target genes in MASLD patients and mice models.

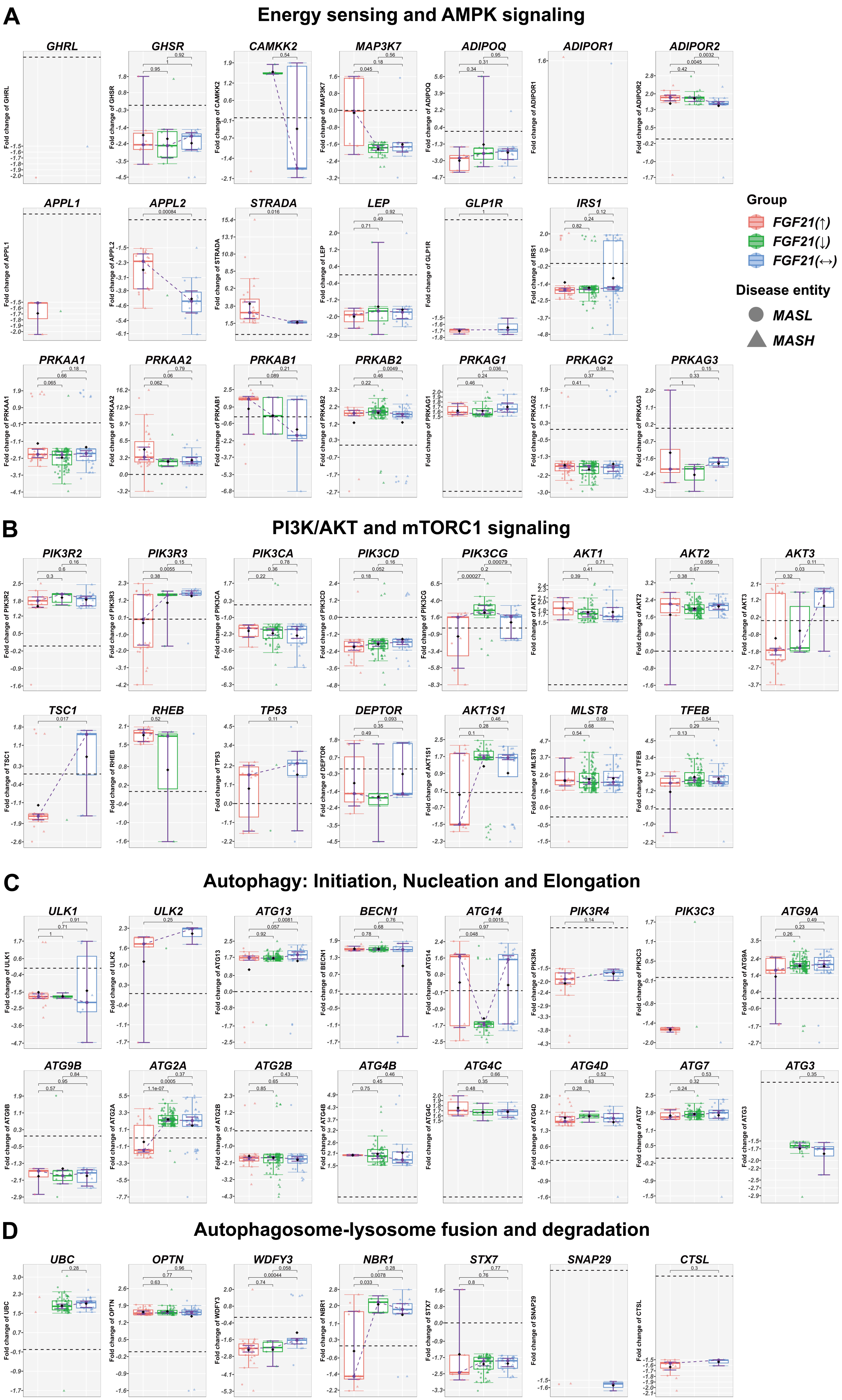
A
Energy sensing and AMPK signaling
GHRL
GHSR
CAMKK2
MAP3K7
ADIPOQ
ADIPOR1
ADIPOR2
APPL1
APPL2
STRADA
LEP
GLP1R
IRS1
PRKAA1
PRKAA2
PRKAB1
PRKAB2
PRKAG1
PRKAG2
PRKAG3
Group
FGF21(↑)
FGF21(↓)
FGF21(↔)
Disease entity
MASL
MASH
B
PI3K/AKT and mTORC1 signaling
PIK3R2
PIK3R3
PIK3CA
PIK3CD
PIK3CG
AKT1
AKT2
AKT3
TSC1
RHEB
TP53
DEPTOR
AKT1S1
MLST8
TFEB
C
Autophagy: Initiation, Nucleation and Elongation
ULK1
ULK2
ATG13
BECN1
ATG14
PIK3R4
PIK3C3
ATG9A
ATG9B
ATG2A
ATG2B
ATG4B
ATG4C
ATG4D
ATG7
ATG3
D
Autophagosome-lysosome fusion and degradation
UBC
OPTN
WDFY3
NBR1
STX7
SNAP29
CTSL

Figure S3 The box-plots represent changes in DEGs coding for energy sensing and autophagy in MASLD according to the patients FGF21-expression status.

(A) DEGs coding for energy sensing and AMPK signaling. (B) DEGs coding for PI3K/AKT and mTORC1 signaling. (C) DEGs coding for autophagy different phases. (D) DEGs coding for autophagosome-lysosome fusion and degradation. Red colored box plots represent increased, green decreased and grey unchanged FGF21 expression. p-values were calculated in pair-wise comparisons with the Wilcoxon rank-sum test.

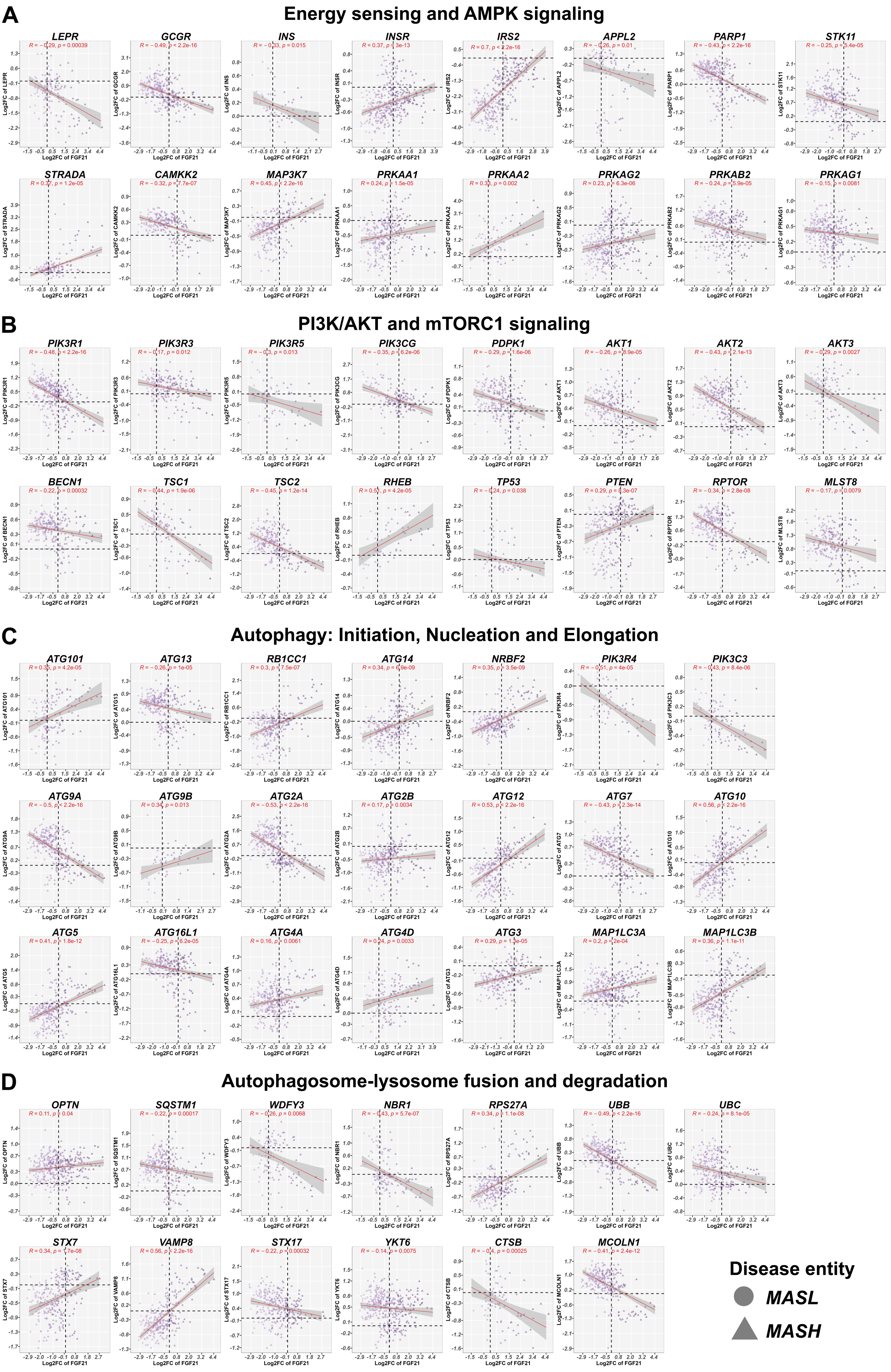
A
Energy sensing and AMPK signaling
LEPR
GCGR
INS
INSR
IRS2
APPL2
PARP1
STK11
STRADA
CAMKK2
MAP3K7
PRKAA1
PRKAA2
PRKAG2
PRKAB2
PRKAG1
B
PI3K/AKT and mTORC1 signaling
PIK3R1
PIK3R3
PIK3R5
PIK3CG
PDPK1
AKT1
AKT2
AKT3
BECN1
TSC1
TSC2
RHEB
TP53
PTEN
RPTOR
MLST8
C
Autophagy: Initiation, Nucleation and Elongation
ATG101
ATG13
RB1CC1
ATG14
NRBF2
PIK3R4
PIK3C3
ATG9A
ATG9B
ATG2A
ATG2B
ATG12
ATG7
ATG10
ATG5
ATG16L1
ATG4A
ATG4D
ATG3
MAP1LC3A
MAP1LC3B
D
Autophagosome-lysosome fusion and degradation
OPTN
SQSTM1
WDFY3
NBR1
RPS27A
UBB
UBC
STX7
VAMP8
STX17
YKT6
CTSB
MCOLN1
Log2FC of FGF21
Disease entity
MASL
MASH

Figure S4 The correlations highlight DEGs coding for energy sensing and autophagy linked to FGF21 expression in MASLD patients.

(A) DEGs coding for energy sensing and AMPK signaling. (B) DEGs coding for PI3K/AKT and mTORC1 signaling. (C) DEGs coding for autophagy different phases. (D) DEGs coding for autophagosome-lysosome fusion and degradation. Spearman correlations were computed in R and only correlation results with p-value <0.05 were shown.

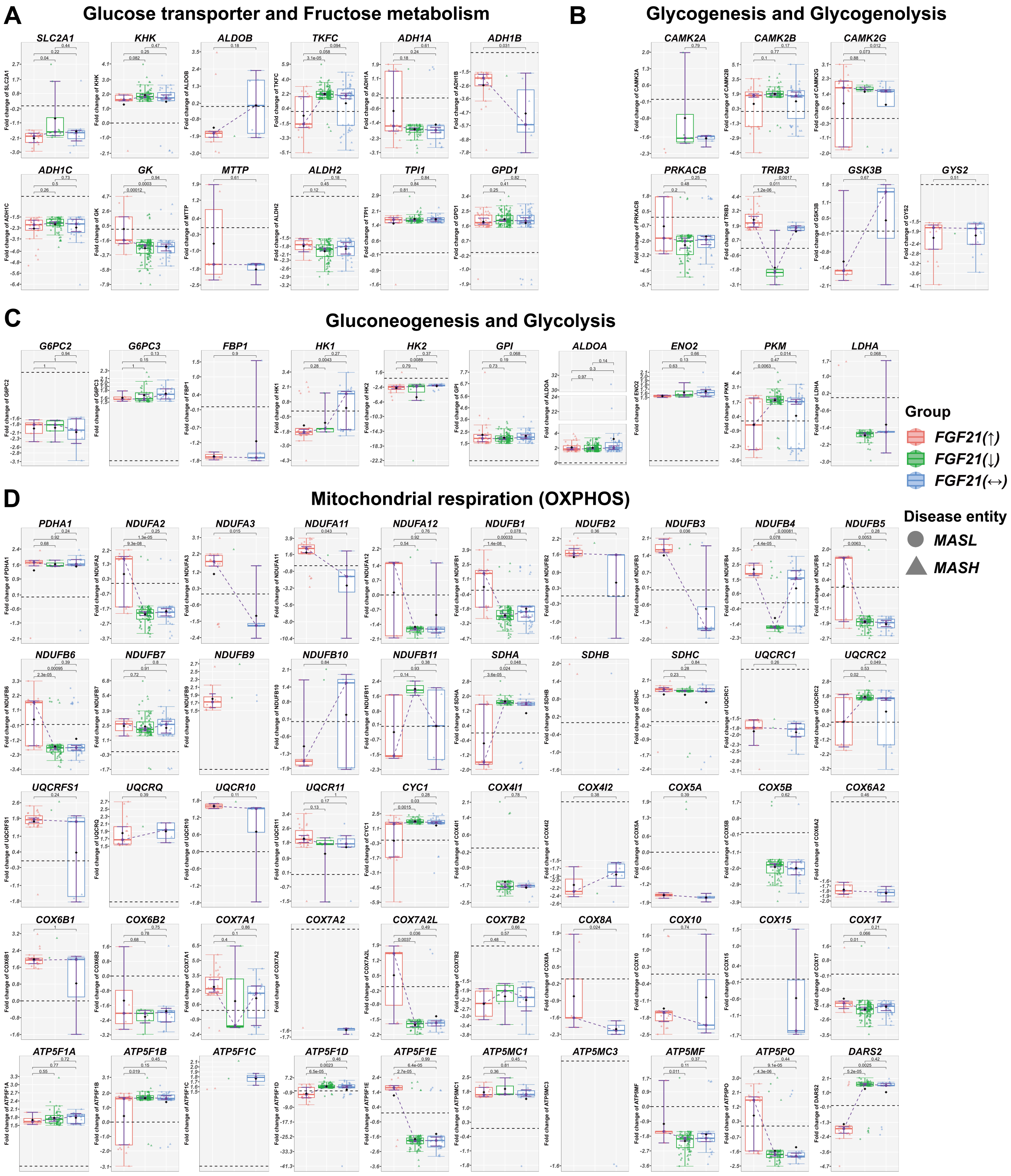
A
Glucose transporter and Fructose metabolism
SLC2A1
KHK
ALDOB
TKFC
ADH1A
ADH1B
ADH1C
GK
MTTP
ALDH2
TPI1
GPD1
B
Glycogenesis and Glycogenolysis
CAMK2A
CAMK2B
CAMK2G
PRKACB
TRIB3
GSK3B
GYS2
C
Gluconeogenesis and Glycolysis
G6PC2
G6PC3
FBP1
HK1
HK2
GPI
ALDOA
ENO2
PKM
LDHA
Group
FGF21(↑)
FGF21(↓)
FGF21(↔)
Disease entity
MASL
MASH
D
Mitochondrial respiration (OXPHOS)
PDHA1
NDUFA2
NDUFA3
NDUFA11
NDUFA12
NDUFB1
NDUFB2
NDUFB3
NDUFB4
NDUFB5
NDUFB6
NDUFB7
NDUFB9
NDUFB10
NDUFB11
SDHA
SDHB
SDHC
UQCRC1
UQCRC2
UQCRFS1
UQCRQ
UQCR10
UQCR11
CYC1
COX4I1
COX4I2
COX5A
COX5B
COX6A2
COX6B1
COX6B2
COX7A1
COX7A2
COX7A2L
COX7B2
COX8A
COX10
COX15
COX17
ATP5F1A
ATP5F1B
ATP5F1C
ATP5F1D
ATP5F1E
ATP5MC1
ATP5MC3
ATP5MF
ATP5PO
DARS2

Figure S5 The box-plots represent changes in DEGs coding for glucose metabolism and mitochondrial respiration in MASLD according to the patients FGF21-expression status.

(A) DEGs coding for glucose/fructose metabolism. (B) DEGs coding for glycogenesis and glycogenolysis. (C) DEGs coding for gluconeogenesis and glycolysis. (D) DEGs coding for mitochondrial respiration (OXPHOS). Red colored box plots represent increased, green decreased and grey unchanged FGF21 expression. p-values were calculated in pair-wise comparisons with the Wilcoxon rank-sum test.

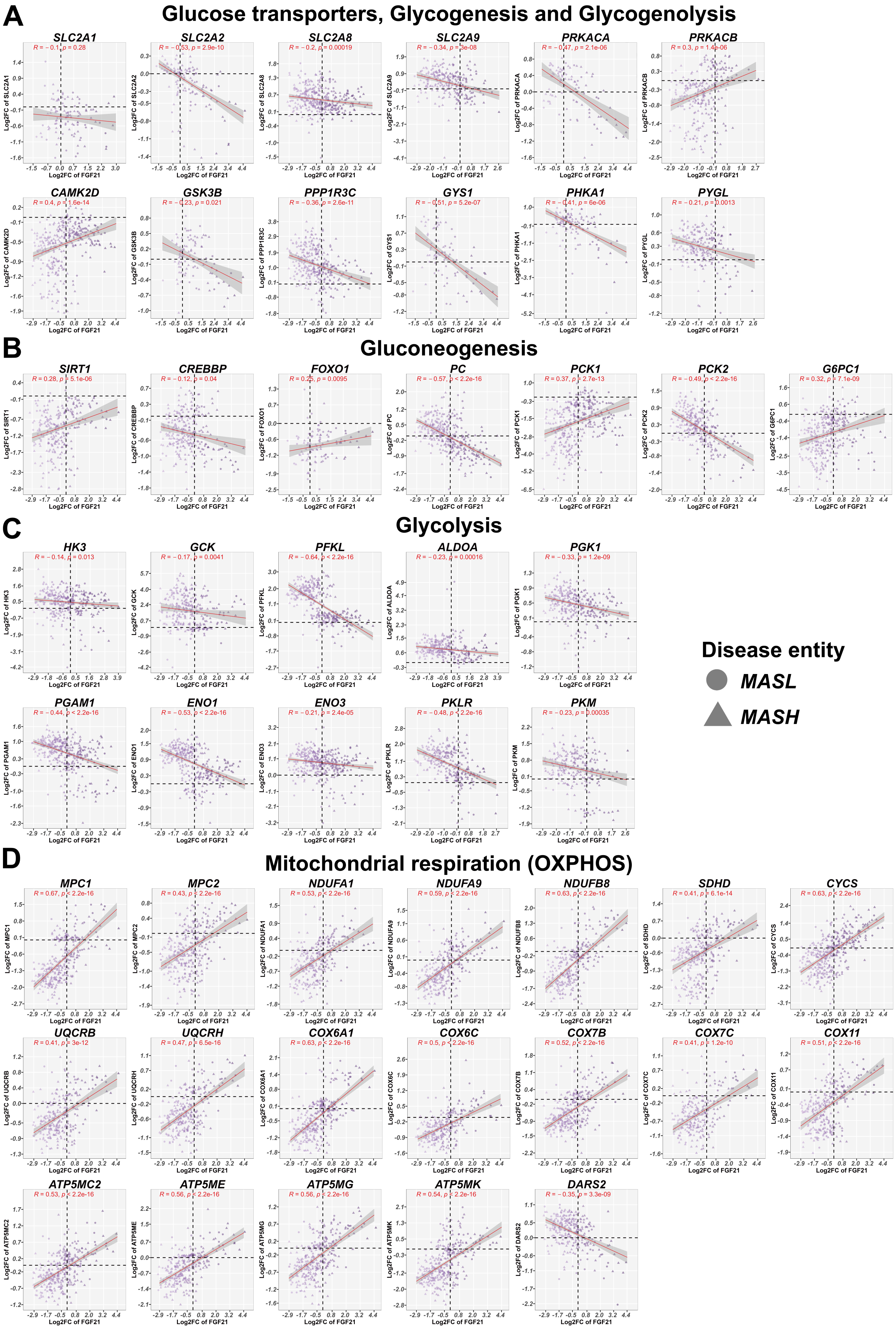

A
Glucose transporters, Glycogenesis and Glycogenolysis
SLC2A1
R = − 0.1, p = 0.28
SLC2A2
R = − 0.53, p = 2.9e-10
SLC2A8
R = − 0.2, p = 0.00019
SLC2A9
R = − 0.34, p = 3e-08
PRKACA
R = − 0.47, p = 2.1e-06
PRKACB
R = 0.3, p = 1.4e-06
CAMK2D
R = 0.4, p = 1.6e-14
GSK3B
R = − 0.23, p = 0.021
PPP1R3C
R = − 0.36, p = 2.6e-11
GYS1
R = − 0.51, p = 5.2e-07
PHKA1
R = − 0.41, p = 6e-06
PYGL
R = − 0.21, p = 0.0013
B
Gluconeogenesis
SIRT1
R = 0.28, p = 5.1e-06
CREBBP
R = − 0.12, p = 0.04
FOXO1
R = 0.25, p = 0.0095
PC
R = − 0.57, p < 2.2e-16
PCK1
R = 0.37, p = 2.7e-13
PCK2
R = − 0.49, p < 2.2e-16
G6PC1
R = 0.32, p = 7.1e-09
C
Glycolysis
HK3
R = − 0.14, p = 0.013
GCK
R = − 0.17, p = 0.0041
PFKL
R = − 0.64, p < 2.2e-16
ALDOA
R = − 0.23, p = 0.00016
PGK1
R = − 0.33, p = 1.2e-09
PGAM1
R = − 0.44, p < 2.2e-16
ENO1
R = − 0.53, p < 2.2e-16
ENO3
R = − 0.21, p = 2.4e-05
PKLR
R = − 0.48, p < 2.2e-16
PKM
R = − 0.23, p = 0.00035
Disease entity
MASL
MASH
D
Mitochondrial respiration (OXPHOS)
MPC1
R = 0.67, p < 2.2e-16
MPC2
R = 0.43, p < 2.2e-16
NDUFA1
R = 0.53, p < 2.2e-16
NDUFA9
R = 0.59, p < 2.2e-16
NDUFB8
R = 0.63, p < 2.2e-16
SDHD
R = 0.41, p = 6.1e-14
CYCS
R = 0.63, p < 2.2e-16
UQCRB
R = 0.41, p = 3e-12
UQCRH
R = 0.47, p = 6.5e-16
COX6A1
R = 0.63, p < 2.2e-16
COX6C
R = 0.5, p < 2.2e-16
COX7B
R = 0.52, p < 2.2e-16
COX7C
R = 0.41, p = 1.2e-10
COX11
R = 0.51, p < 2.2e-16
ATP5MC2
R = 0.53, p < 2.2e-16
ATP5ME
R = 0.56, p < 2.2e-16
ATP5MG
R = 0.56, p < 2.2e-16
ATP5MK
R = 0.54, p < 2.2e-16
DARS2
R = − 0.35, p = 3.3e-09
Log2FC of FGF21

Figure S6 The correlations highlight DEGs coding for glucose metabolism and mitochondrial respiration linked to FGF21 expression in MASLD patients.

(A) DEGs coding for glucose transporters, glycogenesis and glycogenolysis. (B) DEGs coding for gluconeogenesis. (C) DEGs coding for glycolysis. (D) DEGs coding for mitochondrial respiration (OXPHOS). Spearman correlations were computed in R and only correlation results with p-value <0.05 were shown.

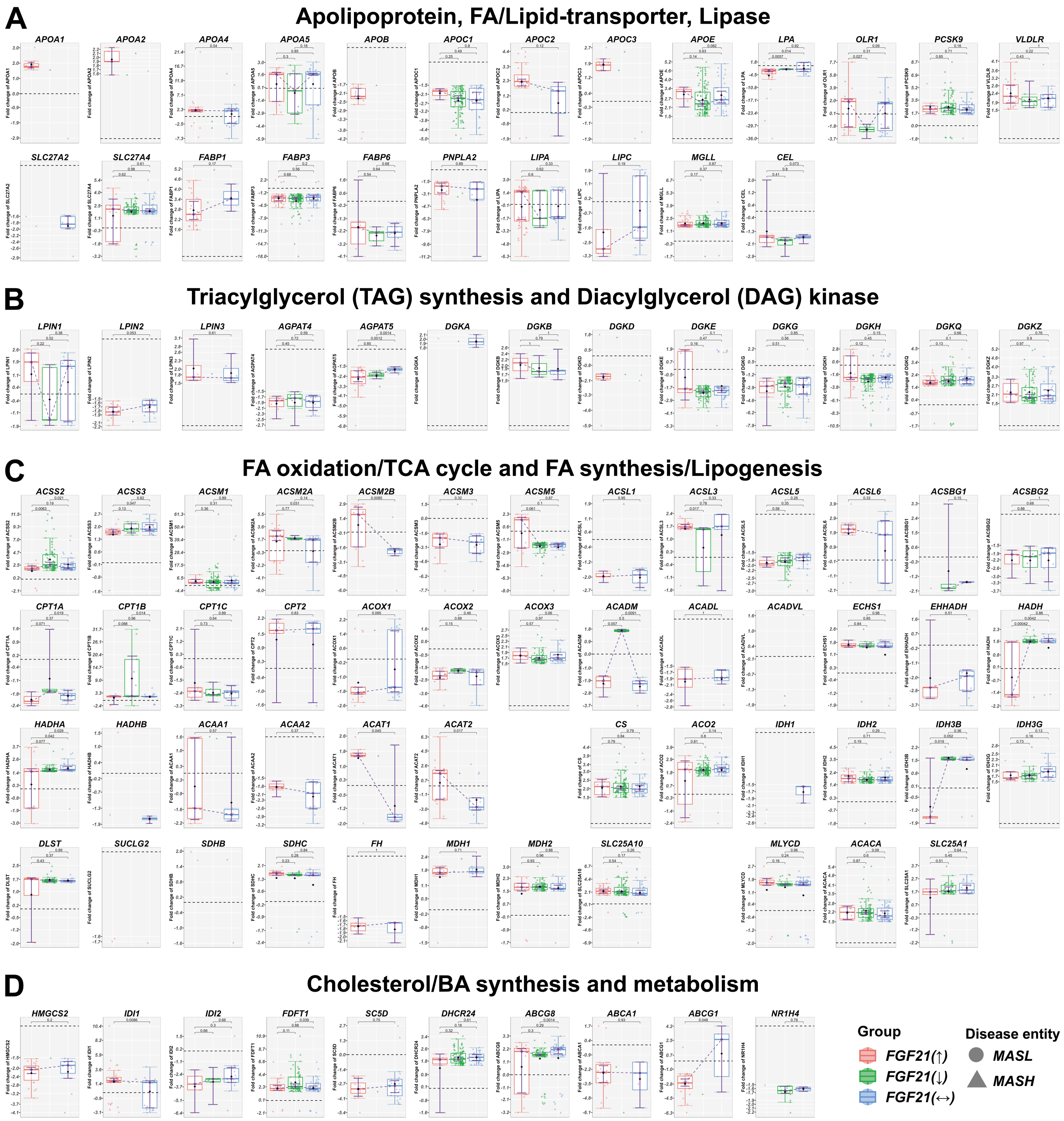
A
Apolipoprotein, FA/Lipid-transporter, Lipase
APOA1
APOA2
APOA4
APOA5
APOB
APOC1
APOC2
APOC3
APOE
LPA
OLR1
PCSK9
VLDLR
SLC27A2
SLC27A4
FABP1
FABP3
FABP6
PNPLA2
LIPA
LIPC
MGLL
CEL
B
Triacylglycerol (TAG) synthesis and Diacylglycerol (DAG) kinase
LPIN1
LPIN2
LPIN3
AGPAT4
AGPAT5
DGKA
DGKB
DGKD
DGKE
DGKG
DGKH
DGKQ
DGKZ
C
FA oxidation/TCA cycle and FA synthesis/Lipogenesis
ACSS2
ACSS3
ACSM1
ACSM2A
ACSM2B
ACSM3
ACSM5
ACSL1
ACSL3
ACSL5
ACSL6
ACSBG1
ACSBG2
CPT1A
CPT1B
CPT1C
CPT2
ACOX1
ACOX2
ACOX3
ACADM
ACADL
ACADVL
ECHS1
EHHADH
HADH
HADHA
HADHB
ACAA1
ACAA2
ACAT1
ACAT2
CS
ACO2
IDH1
IDH2
IDH3B
IDH3G
DLST
SUCLG2
SDHB
SDHC
FH
MDH1
MDH2
SLC25A10
MLYCD
ACACA
SLC25A1
D
Cholesterol/BA synthesis and metabolism
HMGCS2
IDI1
IDI2
FDFT1
SC5D
DHCR24
ABCG8
ABCA1
ABCG1
NR1H4
Group
FGF21(↑)
FGF21(↓)
FGF21(↔)
Disease entity
MASL
MASH

Figure S7 The box-plots represent changes in DEGs coding for lipid and cholesterol/bile acid metabolism in MASLD according to the patients FGF21-expression status.

(A) DEGs coding for apolipoprotein, lipid transport and lipase. (B) DEGs coding for triacylglycerol synthesis. (C) DEGs coding for fatty acid β-oxidation (FAO)/TCA cycle and lipogenesis. (D) DEGs coding for cholesterol/bile acid metabolism. Red colored box plots represent increased, green decreased and grey unchanged FGF21 expression. p-values were calculated in pair-wise comparisons with the Wilcoxon rank-sum test.

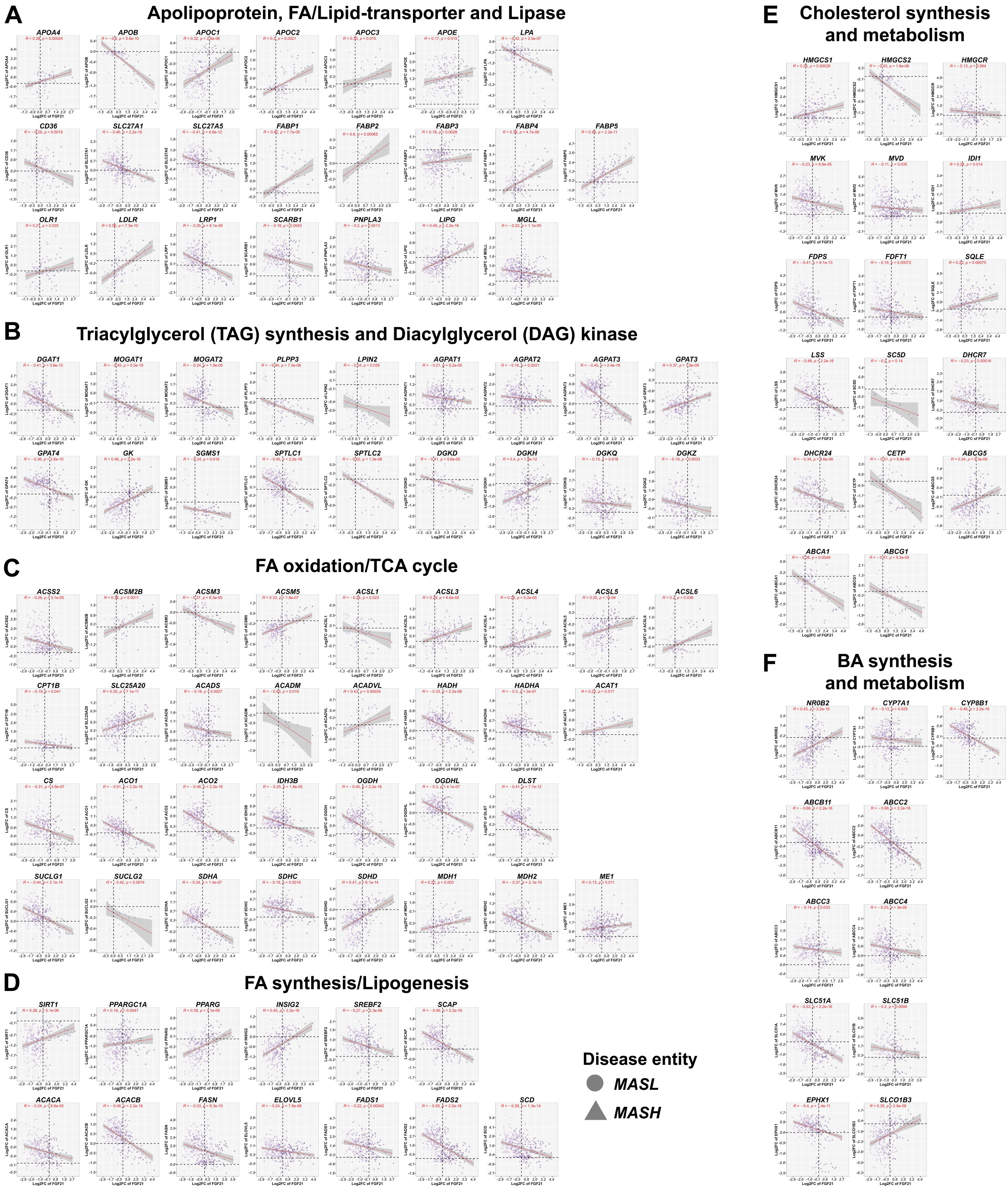

A
Apolipoprotein, FA/Lipid-transporter and Lipase
APOA4
APOB
APOC1
APOC2
APOC3
APOE
LPA
CD36
SLC27A1
SLC27A5
FABP1
FABP2
FABP3
FABP4
FABP5
OLR1
LDLR
LRP1
SCARB1
PNPLA3
LIPG
MGLL
B
Triacylglycerol (TAG) synthesis and Diacylglycerol (DAG) kinase
DGAT1
MOGAT1
MOGAT2
PLPP3
LPIN2
AGPAT1
AGPAT2
AGPAT3
GPAT3
GPAT4
GK
SGMS1
SPTLC1
SPTLC2
DGKD
DGKH
DGKQ
DGKZ
C
FA oxidation/TCA cycle
ACSS2
ACSM2B
ACSM3
ACSM5
ACSL1
ACSL3
ACSL4
ACSL5
ACSL6
CPT1B
SLC25A20
ACADS
ACADM
ACADVL
HADH
HADHA
ACAT1
CS
ACO1
ACO2
IDH3B
OGDH
OGDHL
DLST
SUCLG1
SUCLG2
SDHA
SDHC
SDHD
MDH1
MDH2
ME1
D
FA synthesis/Lipogenesis
SIRT1
PPARGC1A
PPARG
INSIG2
SREBF2
SCAP
ACACA
ACACB
FASN
ELOVL5
FADS1
FADS2
SCD
Disease entity
MASL
MASH
E
Cholesterol synthesis and metabolism
HMGCS1
HMGCS2
HMGCR
MVK
MVD
IDI1
FDPS
FDFT1
SQLE
LSS
SC5D
DHCR7
DHCR24
CETP
ABCG5
ABCA1
ABCG1
F
BA synthesis and metabolism
NR0B2
CYP7A1
CYP8B1
ABCB11
ABCC2
ABCC3
ABCC4
SLC51A
SLC51B
EPHX1
SLCO1B3
Log2FC of FGF21

Figure S8 The correlations highlight DEGs coding for lipid and cholesterol/bile acid metabolism linked to FGF21 expression in MASLD patients.

(A) DEGs coding for apolipoprotein, lipid transport and lipase. (B) DEGs coding for triacylglycerol synthesis. (C) DEGs coding for fatty acid β-oxidation (FAO)/TCA cycle. (D) DEGs coding for lipogenesis. (E) DEGs coding for cholesterol synthesis and metabolism. (F) DEGs coding for bile acid synthesis and metabolism. Spearman correlations were computed in R and only correlation results with p-value <0.05 were shown.

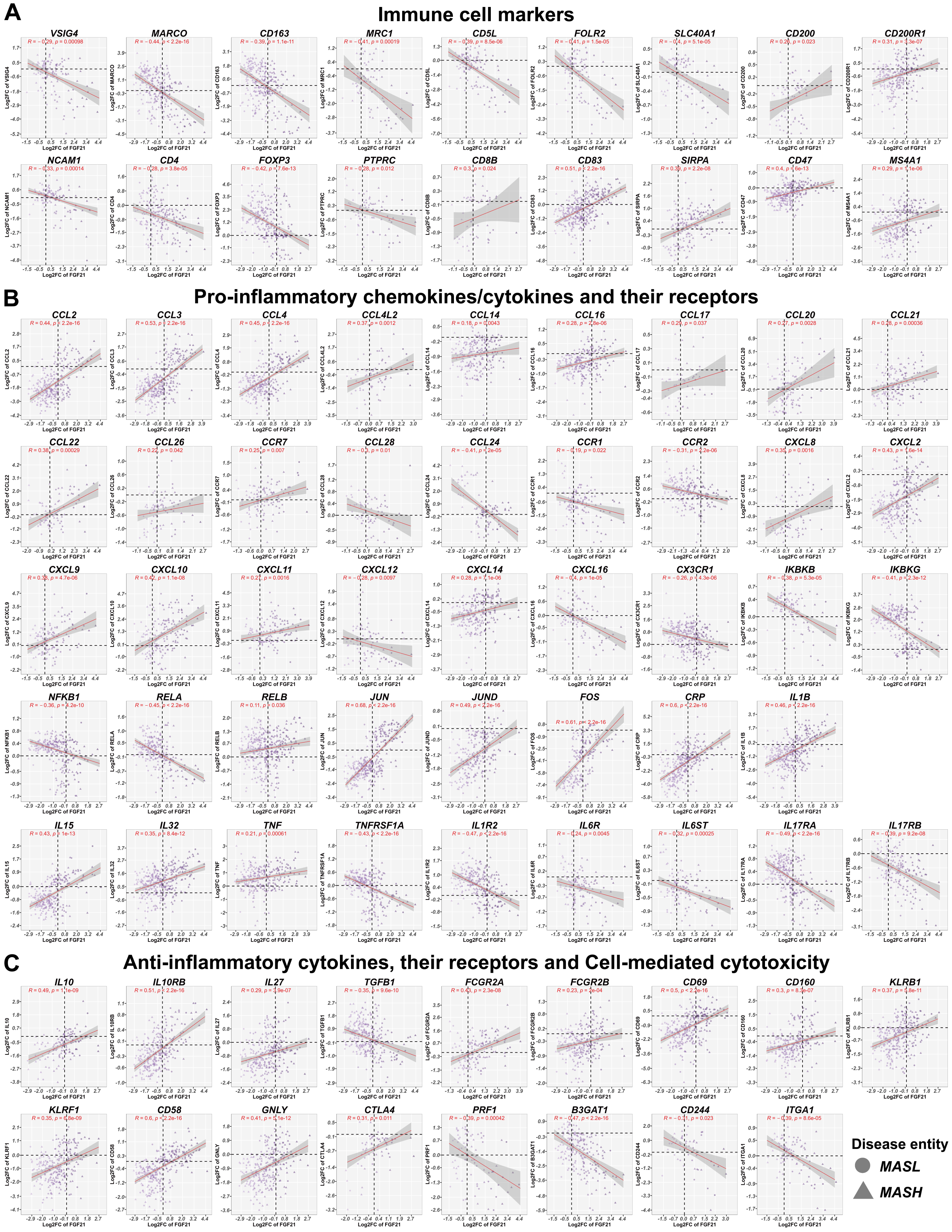
A
Immune cell markers
VSIG4
MARCO
CD163
MRC1
CD5L
FOLR2
SLC40A1
CD200
CD200R1
NCAM1
CD4
FOXP3
PTPRC
CD8B
CD83
SIRPA
CD47
MS4A1
B
Pro-inflammatory chemokines/cytokines and their receptors
CCL2
CCL3
CCL4
CCL4L2
CCL14
CCL16
CCL17
CCL20
CCL21
CCL22
CCL26
CCR7
CCL28
CCL24
CCR1
CCR2
CXCL8
CXCL2
CXCL9
CXCL10
CXCL11
CXCL12
CXCL14
CXCL16
CX3CR1
IKBKB
IKBKG
NFKB1
RELA
RELB
JUN
JUND
FOS
CRP
IL1B
IL15
IL32
TNF
TNFRSF1A
IL1R2
IL6R
IL6ST
IL17RA
IL17RB
C
Anti-inflammatory cytokines, their receptors and Cell-mediated cytotoxicity
IL10
IL10RB
IL27
TGFB1
FCGR2A
FCGR2B
CD69
CD160
KLRB1
KLRF1
CD58
GNLY
CTLA4
PRF1
B3GAT1
CD244
ITGA1
Log2FC of FGF21
Disease entity
MASL
MASH

Figure S9 The correlations highlight DEGs coding for pro- and anti-inflammatory responses linked to FGF21 expression in MASLD patients.

(A) DEGs coding for immune cell markers. (B) DEGs coding for pro-inflammatory chemokines/cytokines and their receptors. (C) DEGs coding for anti-inflammatory cytokines, their receptors and cell mediated cytotoxicity. Spearman correlations were computed in R and only correlation results with p-value <0.05 were shown.

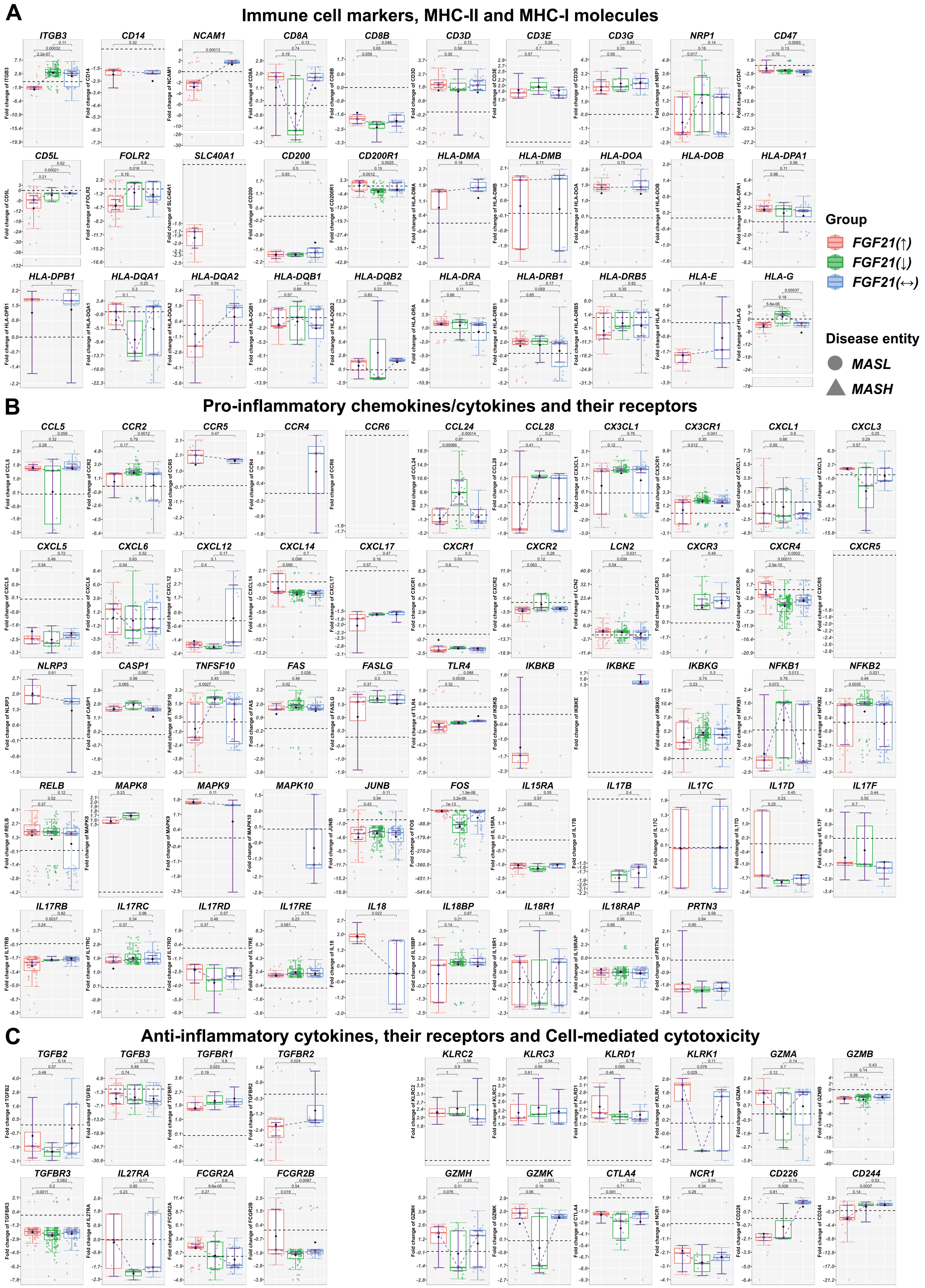
A
Immune cell markers, MHC-II and MHC-I molecules
ITGB3
CD14
NCAM1
CD8A
CD8B
CD3D
CD3E
CD3G
NRP1
CD47
CD5L
FOLR2
SLC40A1
CD200
CD200R1
HLA-DMA
HLA-DMB
HLA-DOA
HLA-DOB
HLA-DPA1
HLA-DPB1
HLA-DQA1
HLA-DQA2
HLA-DQB1
HLA-DQB2
HLA-DRA
HLA-DRB1
HLA-DRB5
HLA-E
HLA-G
Group
FGF21(↑)
FGF21(↓)
FGF21(↔)
Disease entity
MASL
MASH
B
Pro-inflammatory chemokines/cytokines and their receptors
CCL5
CCR2
CCR5
CCR4
CCR6
CCL24
CCL28
CX3CL1
CX3CR1
CXCL1
CXCL3
CXCL5
CXCL6
CXCL12
CXCL14
CXCL17
CXCR1
CXCR2
LCN2
CXCR3
CXCR4
CXCR5
NLRP3
CASP1
TNFSF10
FAS
FASLG
TLR4
IKBKB
IKBKE
IKBKG
NFKB1
NFKB2
RELB
MAPK8
MAPK9
MAPK10
JUNB
FOS
IL15RA
IL17B
IL17C
IL17D
IL17F
IL17RB
IL17RC
IL17RD
IL17RE
IL18
IL18BP
IL18R1
IL18RAP
PRTN3
C
Anti-inflammatory cytokines, their receptors and Cell-mediated cytotoxicity
TGFB2
TGFB3
TGFBR1
TGFBR2
KLRC2
KLRC3
KLRD1
KLRK1
GZMA
GZMB
TGFBR3
IL27RA
FCGR2A
FCGR2B
GZMH
GZMK
CTLA4
NCR1
CD226
CD244

Figure S10 The box-plots represent changes in DEGs coding for pro- and anti-inflammatory responses in MASLD according to the patients FGF21-expression status.

(A) DEGs coding for immune cell markers and MHC-II/I molecules. (B) DEGs coding for pro-inflammatory chemokines/cytokines and their receptors. (C) DEGs coding for anti-inflammatory cytokines, their receptors and cell mediated cytotoxicity. Red colored box plots represent increased, green decreased and grey unchanged FGF21 expression. p-values were calculated in pair-wise comparisons with the Wilcoxon rank-sum test.

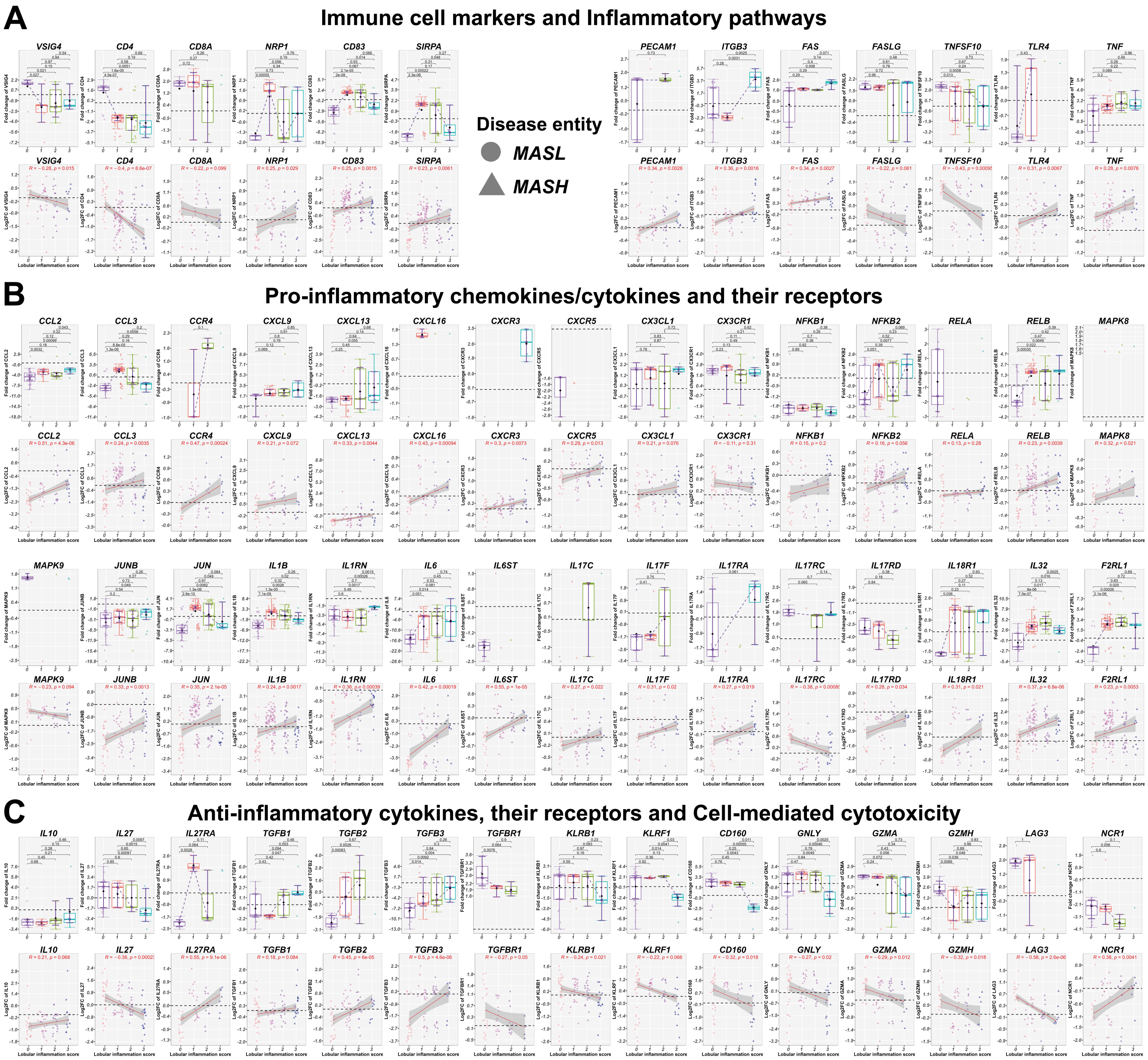
A
Immune cell markers and Inflammatory pathways
VSIG4 CD4 CD8A NRP1 CD83 SIRPA
PECAM1 ITGB3 FAS FASLG TNFSF10 TLR4 TNF
Disease entity
MASL
MASH
Lobular inflammation score
B
Pro-inflammatory chemokines/cytokines and their receptors
CCL2 CCL3 CCR4 CXCL9 CXCL13 CXCL16 CXCR3 CXCR5 CX3CL1 CX3CR1 NFKB1 NFKB2 RELA RELB MAPK8
MAPK9 JUNB JUN IL1B IL1RN IL6 IL6ST IL17C IL17F IL17RA IL17RC IL17RD IL18R1 IL32 F2RL1
C
Anti-inflammatory cytokines, their receptors and Cell-mediated cytotoxicity
IL10 IL27 IL27RA TGFB1 TGFB2 TGFB3 TGFBR1 KLRB1 KLRF1 CD160 GNLY GZMA GZMH LAG3 NCR1

Figure S11 DEGs coding for pro- and anti-inflammatory responses linked to lobular inflammation scores (=0-3) in MASLD patients.

(A) DEGs coding for immune cell markers and inflammatory pathways. (B) DEGs coding for pro-inflammatory chemokines/cytokines and their receptors. (C) DEGs coding for anti-inflammatory cytokines, their receptors and cell mediated cytotoxicity. The box-plots represent changes in DEGs according to different lobular inflammation scores. p-values were calculated in pair-wise comparisons with the Wilcoxon rank-sum test. Spearman correlations were computed in R.

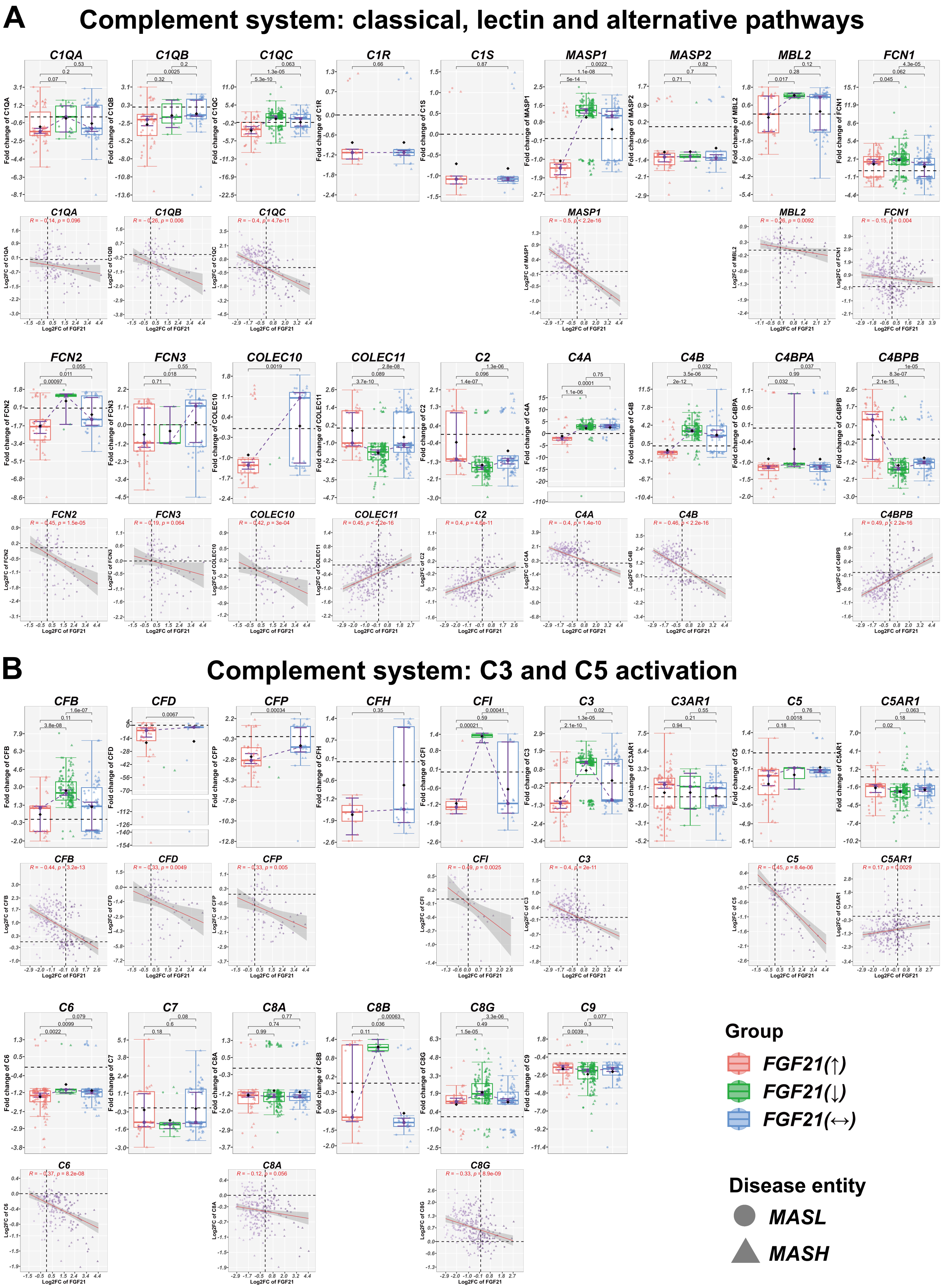

A
Complement system: classical, lectin and alternative pathways
C1QA
C1QB
C1QC
C1R
C1S
MASP1
MASP2
MBL2
FCN1
FCN2
FCN3
COLEC10
COLEC11
C2
C4A
C4B
C4BPA
C4BPB
Log2FC of FGF21
B
Complement system: C3 and C5 activation
CFB
CFD
CFP
CFH
CFI
C3
C3AR1
C5
C5AR1
C6
C7
C8A
C8B
C8G
C9
Group
FGF21(↑)
FGF21(↓)
FGF21(↔)
Disease entity
MASL
MASH

Figure S12 Complement factors in MASLD according to the patients FGF21-expression status.

(A) DEGs coding for classical, lectin and alternative complement pathways. (B) DEGs coding for pathways of C3 and C5 activation. The box-plots represent changes in DEGs according to the patients FGF21-expression status, i.e. increased, decreased or unchanged. Red colored box plots represent increased, green decreased and grey unchanged FGF21 expression. p-values were calculated in pair-wise comparisons with the Wilcoxon rank-sum test. Spearman correlations were computed in R and only correlation results with p-value <0.05 were shown.

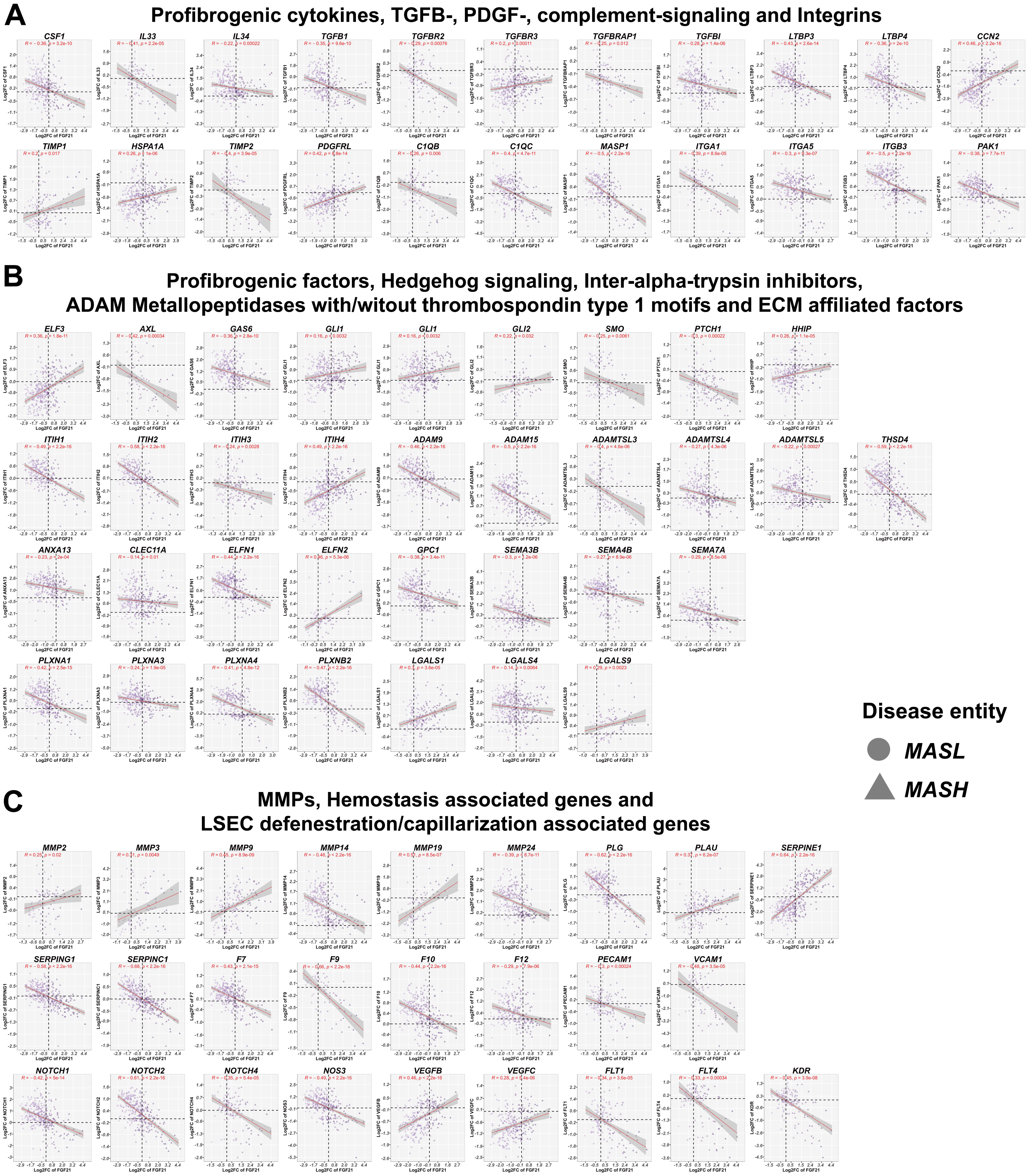
A
Profibrogenic cytokines, TGFB-, PDGF-, complement-signaling and Integrins
CSF1
IL33
IL34
TGFB1
TGFBR2
TGFBR3
TGFBRAP1
TGFBI
LTBP3
LTBP4
CCN2
TIMP1
HSPA1A
TIMP2
PDGFRL
C1QB
C1QC
MASP1
ITGA1
ITGA5
ITGB3
PAK1
B
Profibrogenic factors, Hedgehog signaling, Inter-alpha-trypsin inhibitors,
ADAM Metallopeptidases with/witout thrombospondin type 1 motifs and ECM affiliated factors
ELF3
AXL
GAS6
GLI1
GLI1
GLI2
SMO
PTCH1
HHIP
ITIH1
ITIH2
ITIH3
ITIH4
ADAM9
ADAM15
ADAMTSL3
ADAMTSL4
ADAMTSL5
THSD4
ANXA13
CLEC11A
ELFN1
ELFN2
GPC1
SEMA3B
SEMA4B
SEMA7A
PLXNA1
PLXNA3
PLXNA4
PLXNB2
LGALS1
LGALS4
LGALS9
Disease entity
MASL
MASH
C
MMPs, Hemostasis associated genes and
LSEC defenestration/capillarization associated genes
MMP2
MMP3
MMP9
MMP14
MMP19
MMP24
PLG
PLAU
SERPINE1
SERPING1
SERPINC1
F7
F9
F10
F12
PECAM1
VCAM1
NOTCH1
NOTCH2
NOTCH4
NOS3
VEGFB
VEGFC
FLT1
FLT4
KDR
Log2FC of FGF21

Figure S13 The correlations highlight DEGs coding for fibrogenesis and fibrosis resolution linked to FGF21 expression in MASLD patients.

(A) DEGs coding for pro-fibrogenic cytokines and TGFβ-, PDGF-, complement- and integrins-signaling. (B) DEGs coding for Hedgehog-signaling, inter-alpha-trypsin-inhibitors, ADAMs/ADAMTSs and ECM-affiliated factors. (C) DEGs coding for MMPs, hemostasis and LSEC defenestration. Spearman correlations were computed in R and only correlation results with p-value <0.05 were shown.

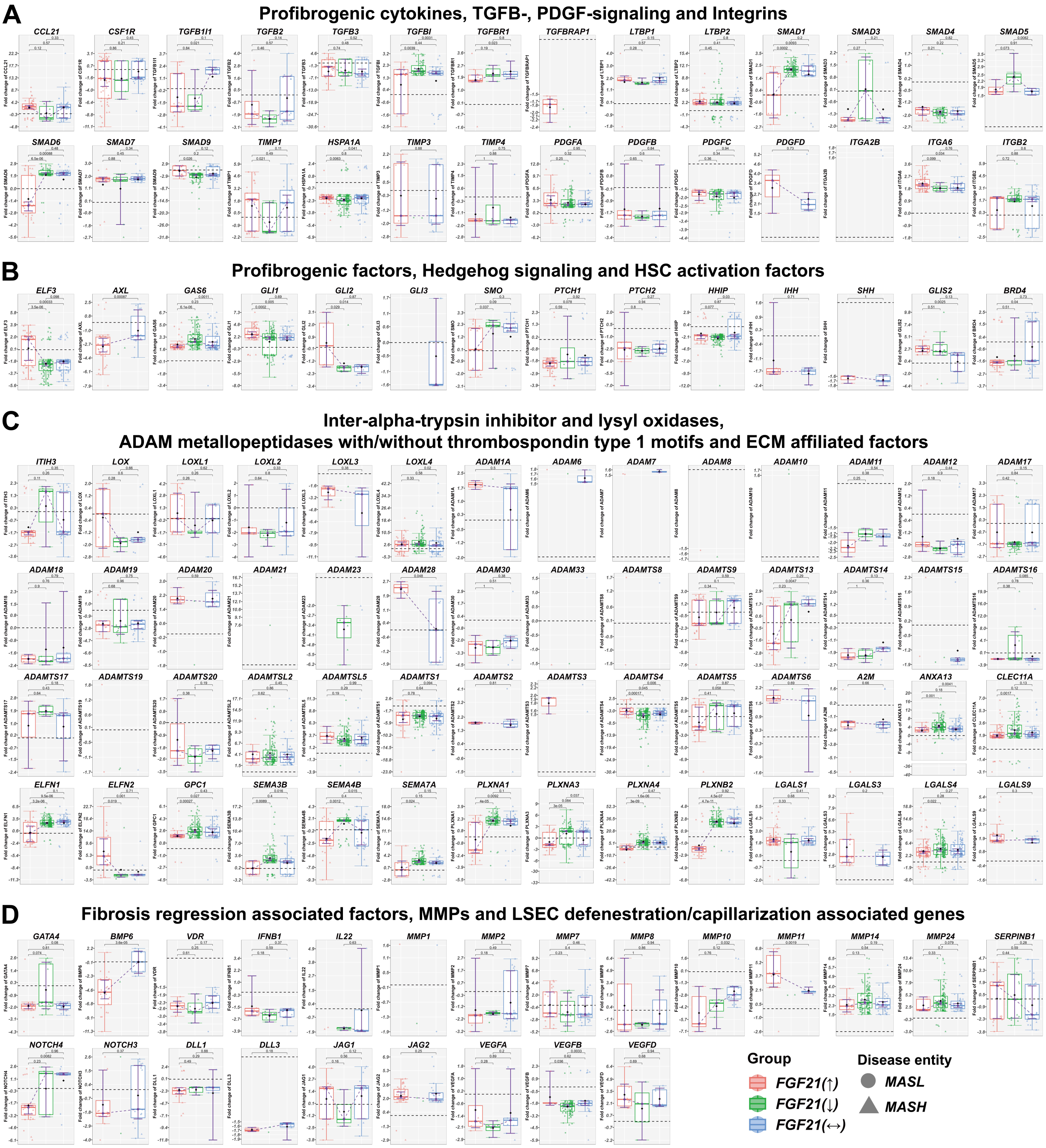
A
Profibrogenic cytokines, TGFB-, PDGF-signaling and Integrins
CCL21 CSF1R TGFB1I1 TGFB2 TGFB3 TGFBI TGFBR1 TGFBRAP1 LTBP1 LTBP2 SMAD1 SMAD3 SMAD4 SMAD5
SMAD6 SMAD7 SMAD9 TIMP1 HSPA1A TIMP3 TIMP4 PDGFA PDGFB PDGFC PDGFD ITGA2B ITGA6 ITGB2
B
Profibrogenic factors, Hedgehog signaling and HSC activation factors
ELF3 AXL GAS6 GLI1 GLI2 GLI3 SMO PTCH1 PTCH2 HHIP IHH SHH GLIS2 BRD4
C
Inter-alpha-trypsin inhibitor and lysyl oxidases,
ADAM metallopeptidases with/without thrombospondin type 1 motifs and ECM affiliated factors
ITIH3 LOX LOXL1 LOXL2 LOXL3 LOXL4 ADAM1A ADAM6 ADAM7 ADAM8 ADAM10 ADAM11 ADAM12 ADAM17
ADAM18 ADAM19 ADAM20 ADAM21 ADAM23 ADAM28 ADAM30 ADAM33 ADAMTS8 ADAMTS9 ADAMTS13 ADAMTS14 ADAMTS15 ADAMTS16
ADAMTS17 ADAMTS19 ADAMTS20 ADAMTSL2 ADAMTSL5 ADAMTS1 ADAMTS2 ADAMTS3 ADAMTS4 ADAMTS5 ADAMTS6 A2M ANXA13 CLEC11A
ELFN1 ELFN2 GPC1 SEMA3B SEMA4B SEMA7A PLXNA1 PLXNA3 PLXNA4 PLXNB2 LGALS1 LGALS3 LGALS4 LGALS9
D
Fibrosis regression associated factors, MMPs and LSEC defenestration/capillarization associated genes
GATA4 BMP6 VDR IFNB1 IL22 MMP1 MMP2 MMP7 MMP8 MMP10 MMP11 MMP14 MMP24 SERPINB1
NOTCH4 NOTCH3 DLL1 DLL3 JAG1 JAG2 VEGFA VEGFB VEGFD
Group
FGF21(↑)
FGF21(↓)
FGF21(↔)
Disease entity
MASL
MASH

Figure S14 The box-plots represent changes in DEGs coding for fibrogenesis and fibrosis resolution in MASLD according to the patients FGF21-expression status.

(A) DEGs coding for pro-fibrogenic cytokines and TGFβ-, PDGF-, and integrins-signaling. (B) DEGs coding for pro-fibrogenic factors and Hedgehog-signaling. (C) DEGs coding for inter-alpha-trypsin-inhibitors, ADAMs/ADAMTSs and ECM-affiliated factors. (D) DEGs coding for fibrosis regression factors, MMPs, hemostasis and LSEC defenestration. Red colored box plots represent increased, green decreased and grey unchanged FGF21 expression. p-values were calculated in pair-wise comparisons with the Wilcoxon rank-sum test.

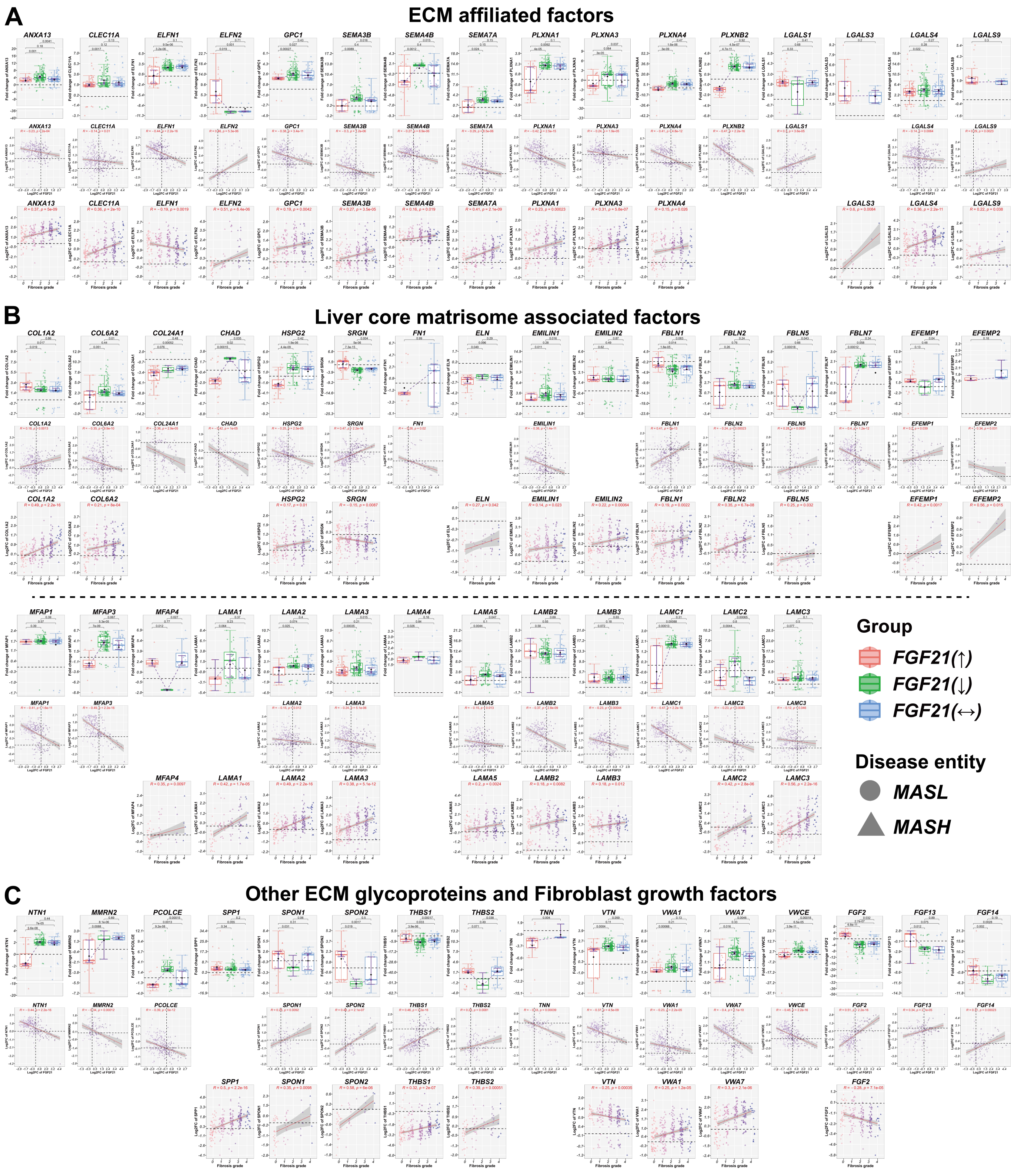
A
ECM affiliated factors
ANXA13
CLEC11A
ELFN1
ELFN2
GPC1
SEMA3B
SEMA4B
SEMA7A
PLXNA1
PLXNA3
PLXNA4
PLXNB2
LGALS1
LGALS3
LGALS4
LGALS9
Log2FC of FGF21
Fibrosis grade
B
Liver core matrisome associated factors
COL1A2
COL6A2
COL24A1
CHAD
HSPG2
SRGN
FN1
ELN
EMILIN1
EMILIN2
FBLN1
FBLN2
FBLN5
FBLN7
EFEMP1
EFEMP2
MFAP1
MFAP3
MFAP4
LAMA1
LAMA2
LAMA3
LAMA4
LAMA5
LAMB2
LAMB3
LAMC1
LAMC2
LAMC3
Group
FGF21(↑)
FGF21(↓)
FGF21(↔)
Disease entity
MASL
MASH
C
Other ECM glycoproteins and Fibroblast growth factors
NTN1
MMRN2
PCOLCE
SPP1
SPON1
SPON2
THBS1
THBS2
TNN
VTN
VWA1
VWA7
VWCE
FGF2
FGF13
FGF14

Figure S15 Liver core matrisome and other fibroblast growth factors in MASLD patients according to the fibrosis grades and hepatic FGF21 expression status.

(A) The correlations highlight DEGs coding for liver core matrisome in MASLD linked to patients' lobular inflammation scores. (B) DEGs coding for liver core matrisome in MASLD according to the patients FGF21-expression status. (C) DEGs coding for other fibroblast growth factors in MASLD patients. The box-plots represent changes in DEGs according to the patients FGF21-expression status, i.e. increased, decreased or unchanged. Red colored box plots represent increased, green decreased and grey unchanged FGF21 expression. p-values were calculated in pair-wise comparisons with the Wilcoxon rank-sum test. Spearman correlations were computed in R.

Up/down/inconsistently-regulated FGF21-targets in ≥10 MASLD patients with increased (FGF21(↑)) or decreased (FGF21(↓)) FGF21 expression

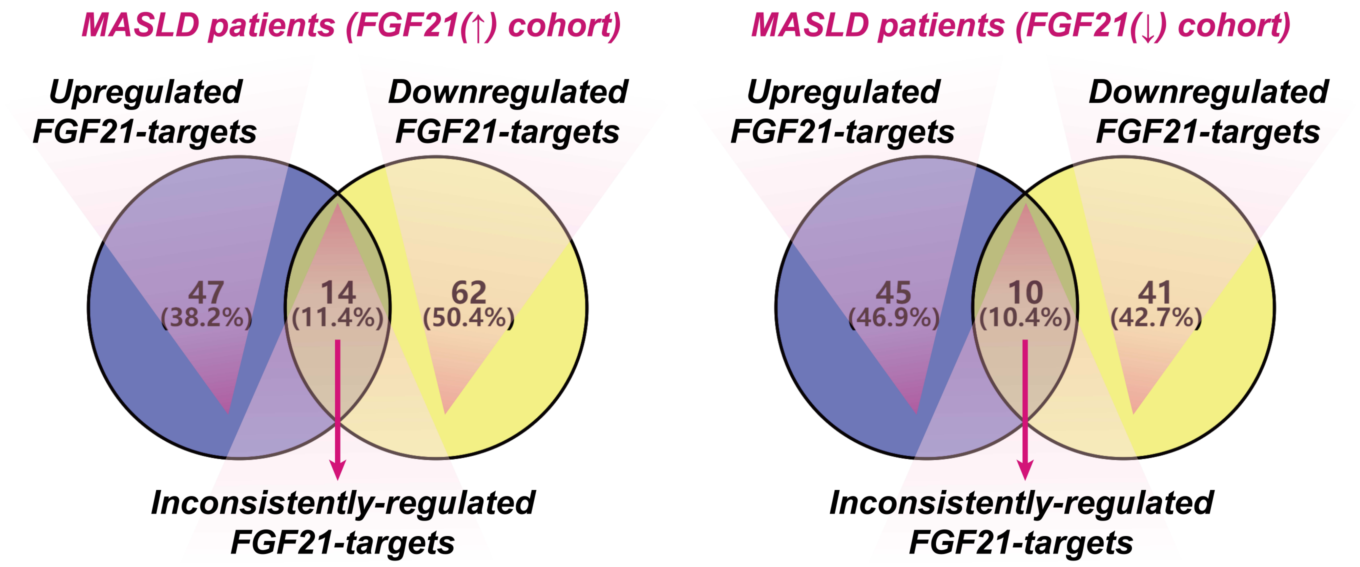

Figure S16 The regulation of FGF21-validated-targets (Table S4) in MASLD patients. Venn diagrams represent upregulated, downregulated or inconsistently-regulated FGF21-targets in MASLD patients with increased or decreased FGF21 expression.
